# Apolipoprotein E4 Suppresses Neuronal-Specific Gene Expression in Maturing Neuronal Progenitor Cells Exposed to HIV

**DOI:** 10.1007/s11481-017-9734-9

**Published:** 2017-03-20

**Authors:** Rebeca Geffin, Ricardo Martinez, Alicia de las Pozas, Biju Issac, Micheline McCarthy

**Affiliations:** 1 Bruce W. Carter Veterans Affairs Medical Center, 1201 NW 16th Street, Miami, FL 33125 USA; 2 Department of Neurology, Miller School of Medicine, University of Miami, 1120 NW 14th St, Miami, FL 33136 USA; 3 Biostatistics and Bioinformatics Core/Division of Bioinformatics, Clinical Research Building, University of Miami/Sylvester Comprehensive Cancer Center, 1120 NW 14th Street, 6th Floor, Room 610L, Miami, FL 33136 USA

**Keywords:** Human Immunodeficiency Virus, Neuron, Apolipoprotein E, Gene expression microarray, Neurogenesis

## Abstract

The apolipoprotein ε4 gene allele and the apolipoprotein E4 protein (ApoE4) are important host susceptibility factors linked to neurocognitive disorders associated with HIV infection or Alzheimer’s disease. Our previous studies showed differential effects of the two most common human ApoE genotypes, APOE3/3 and APOE3/4, on gene expression by differentiating human neuroepithelial progenitor cells continuously exposed to HIV. To investigate the effects of ApoE3 versus ApoE4 isoforms specifically on maturing neurons, we adapted a human neuronal progenitor cell line, hNP1, with ApoE genotype APOE3/3. Differentiating hNP1 cells were exposed for 16 days to HIV- or mock-infected supernatants and to added recombinant ApoE isoforms rApoE3 or rApoE4 to modulate the ApoE phenotype of the cells. Gene expression was investigated using microarray and functional genomics analyses. Added rApoE3 differentially affected 36 genes. Added rApoE4 differentially affected 85 genes; 41 of which were differentially expressed only in HIV or mock-supernatant treated cells, and 80% of which were downregulated. Genes differentially downregulated only by rApoE4 represented multiple neuronal functions related to neurogenesis. Approximately five times more genes were differentially enriched by rApoE4 versus rApoE3 in the Gene Ontology (GO) cellular process analysis, with 4 orders of magnitude greater significance. Half of the top 10 GO processes affected by rApoE4 treatment were neurogenesis-related. The largest differences in gene expression between the two isoforms were observed within the HIV-exposed cultures, suggesting that HIV exposure magnifies ApoE4’s suppressive effect on neuronal gene expression. This study provides evidence for neuronal-specific responses to ApoE4 that could affect neurogenesis and neuronal survival.

**Electronic supplementary material:**

The online version of this article (doi:10.1007/s11481-017-9734-9) contains supplementary material, which is available to authorized users.

## Introduction

Apolipoprotein E (ApoE) is increasingly recognized as an influential if not determining host factor in neuroinflammatory and neurodegenerative disease (Bu 2009). ApoE is a 299 amino acid protein found in liver and brain that is important for the transfer of cholesterol and phospholipids to cells (Mahley 1988). In the central nervous system (CNS), ApoE is synthesized mainly by astrocytes (Boyles et al. 1985; Pitas et al. 1987) and to a much lesser extent by neurons (Xu et al. 1999). ApoE is thought to exert its neuronal effects by binding to cell surface receptors on the surface of neurons (Bu 2009), such that the effects of ApoE on neurons would be largely mediated by exogenous apolipoprotein E secreted by astrocytes.

Different protein isoforms of ApoE exist in the human population, derived from transcription of three different alleles, ε2, ε3, and ε4, which contribute to the genotype (designated APOE), and have a distribution of 8%, 77%, and 15% respectively (Mahley 1988). ApoE protein isoforms differ only by the presence or absence of cysteine residues in positions 112 and 158 of the protein. These seemingly small changes nevertheless result in significant differences in the conformation, physical properties, and biological characteristics of the proteins (Hauser et al. 2011). Notably, the presence of the APOE4 allele has been associated with a higher risk of Alzheimer’s disease (Corder et al. 1993; Poirier et al. 1993), with cerebrovascular disease (Frank et al. 2002), with neuroinflammatory and neurodegenerative disease (Bu 2009), with infectious diseases such as hepatitis C and herpes simplex (Kuhlmann et al. 2010), and with neurocognitive and behavioral abnormalities (HAND) associated with Human Immunodeficiency Virus (HIV) infection (Antinori et al. 2007; Clifford and Ances 2013; Saylor et al. 2016). In a cross-sectional, observational study of 466 participants in the CHARTER cohort (CNS HIV Antiretroviral Therapy Effects Research), logistic regression analyses revealed no association between APOE4 carrier status and HAND (Morgan et al. 2013). However, a clinico-pathological correlative study of 160 HIV-infected adults who received detailed clinical, neuropsychological, and laboratory assessments concluded that APOE4 and older age increased the likelihood of cerebral amyloid-beta (Aβ) plaque deposition in HIV-infected adults (Soontornniyomkij et al. 2012). While Aβ plaques in HIV brains were immunohistologically different from those in symptomatic Alzheimer’s disease brains, Aβ plaques were associated with HAND among APOE4 carriers.

We have observed that when human neuroepithelial progenitor cells (NEP) are allowed to differentiate in vitro into a mixed population of astrocytes and neurons in the presence of HIV, neurons “fail to thrive” (McCarthy et al. 2006; Martinez et al. 2012; Geffin et al. 2013). There are lower total neurite lengths per cell, and moderately decreased amounts of the neurofilament light protein (NF-L) in HIV-exposed neurons with the APOE3/4 genotype compared to those with the APOE3/3 genotype (Martinez et al. 2012). These effects occurred even with no apparent evidence of productive viral infection (McCarthy et al. 2006). Changes in gene expression in differentiated NEP cultures also occur with HIV exposure, and this, too, is influenced by the APOE genotype of the cells. Our previous study using gene expression microarray analysis (Geffin et al. 2013) identified a group of genes in differentiated NEP cultures that are specifically up-regulated by exposure to HIV virus and that are strongly related to interferon-induced responses and antigen presentation. Differentiating NEP harboring the APOE3/3 genotype displayed a more robust upregulation of innate immune response genes than cells with the APOE3/4 genotype.

While our previous study used a mixed glial and neuronal cell population differentiated from human fetal NEP, for the present study we have used a human neural progenitor cell line, hNP1, to further investigate how neural cell responses to HIV exposure are influenced by the two most common ApoE isoforms E3 and E4, now focusing on neuronal-specific gene expression. Although neurons are not widely productively infected by HIV, exposure to HIV proteins can cause neuronal toxicity and even neuronal cell death (Hesselgesser et al. 1998; Maragos et al. 2003; van Marle et al. 2004). The hNP1 cell line, derived from the NIH-registered human embryonic stem cell line WA09, has been established as a neural progenitor line that can be differentiated into an enriched population of human post-mitotic neurons with functional properties such as ion channels and ionotropic receptors (Dhara et al. 2008; Young et al. 2011; Guo et al. 2013). In the present study, hNP1 cells were exposed to HIV_SF2 MC_ or mock-infected culture supernatants during differentiation in the presence of the E3 or E4 isoforms of recombinant human apolipoprotein E protein (rApoE). Addition of rApoE to the neuronal cultures mimics the in vivo environment wherein astrocytes synthesize and secrete ApoE protein, which binds to specific receptors on the neurons. Exogenous rApoE interacting with neurons can have distinct isoform-specific effects. In particular, rApoE4 can downregulate numerous neuronal genes with a wide spectrum of cell functions.

## Materials and Methods

### Culture and Maintenance of hNP1 Cells

#### Proliferation

hNP1 cells were obtained from Aruna Biomedical (Athens, GA). Undifferentiated hNP1 cells were proliferated in monolayer cultures at a density of 4.0X10^4^ cells/cm^2^ in “progenitor medium” consisting of AB2™ Basal Neural Medium supplemented with 2% AB2 supplement (Aruna Biomedical, Athens, GA), 1% glutamine (Gibco ThermoFisher Scientific, Waltham, MA), and 1% penicillin-streptomycin (GibcoThermoFisher Scientific, Waltham, MA). Cultures were fed every 2 days and passaged when 100% confluency was reached, or approximately once per week.

#### Differentiation

For differentiation into neurons, hNP1 cells were seeded onto poly-D,L-ornithine plus fibronectin-coated (PON-FN) coated tissue culture wells at 8.0X10^3^ cells/cm^2^. They were cultured in progenitor medium for 2 days, then switched to sB16 differentiation medium (67% DMEM, 23% F12, 1%(*w*/*v*) bovine serum albumin, 1% penicillin-streptomycin, 0.276%(*w*/*v*) HEPES, 1% N2 supplement, 1% glutamine). Cultures were continued in differentiation medium for up to 21 days. Real time, reverse transcription polymerase chain reaction (RT-PCR) detected neuronal-specific mRNA, coding for neurofilament or microtubular proteins that peaked by day 15 but continued elevated through day 21. Immunoblotting detected full expression of the neurofilament or microtubular neuronal proteins corresponding to the neuronal mRNA at days 15 or 21 (data not shown). Neither mRNA nor protein for the major astrocyte intermediate filament protein, GFAP (glial fibrillary acidic protein) was detected in the lysates from differentiating hNP1 cultures at any timepoint.

### Determination of Apolipoprotein E Genotype of hNP1 cells

ApoE genotyping was performed essentially as described previously (Hixson and Vernier 1990) and adapted for these hNP1 cultures. Genotyping is based on amplification of APOE cellular DNA corresponding to amino acids 112 through 158. Restriction mapping of the amplified DNA with specific enzymes allows the discrimination of the different isoforms (Martinez et al. 2012).

### Differentiation and Culture Treatment

Twenty-four hours after the hNP1 cultures were changed to differentiation medium, which was designated experimental day 0, the differentiating cultures were sorted into 3 groups of triplicate samples for three culture treatments: HIV-exposed, mock-exposed, or untreated. Each culture treatment group was further divided into 3 groups of triplicate samples for incubation with added rApoE (E3 or E4) or no added rApoE. Thus, each culture condition was tested in triplicate. For all culture treatments, with and without rApoE, culture media with additives were fully replenished every 3 days.

#### “HIV-Exposed”

HIV-1 containing supernatants were obtained from human peripheral blood mononuclear cells (PBMC) that were stimulated with mitogens phytohemagglutinin (PHA, Sigma-Aldrich, St Lois, MO) and recombinant human interleukin-2 (IL-2, Roche Diagnostics, Indianapolis, IN), then infected with HIV-1 as described previously (McCarthy et al. 1998). When viral concentration in the infected PBMC supernatants was at least 100 ng/ml, the supernatants were harvested, centrifuged at 400×g for 10 min to eliminate cellular debris, aliquoted, and frozen at -140 °C until needed. Differentiating hNP1 cultures were exposed to HIV by aliquoting human PBMC-derived stock virus into differentiation culture medium at 20 ng p24 per 7.5X10^4^ hNP1 cells. The HIV-1 strain used throughout this study was obtained through the NIH AIDS Reagent Program, Division of AIDS, NIAID, NIH: HIV-1_SF2 MC_, a dual tropic strain, from Dr. Jay Levy. This virus strain recognizes both CXCR4 and CCR5 co-receptors for HIV binding (Trkola et al. 1998).

#### “Mock-Exposed” (control)

Mock-exposed hNP1 cultures were used to control for the effects of non-viral inflammatory factors present in mitogen-stimulated PBMC supernatants (Martinez et al. 2012). Mock-infected PBMC supernatants were derived from PBMC stimulated with PHA and IL-2, prepared at the same time and using the same PBMC donor cells that were used to culture the HIV-1_SF2 MC_ stock virus, but never infected with HIV. Then aliquots of mock-infected PBMC supernatant, equal in volume to the aliquots used for HIV-1_SF2 MC_-infected PBMC supernatants, were added to parallel differentiating hNP1 cultures.

#### “Untreated”

hNP1 cultures contained only differentiation medium with added aliquots of PBMC growth medium (RPMI +20% (*v*/v) fetal bovine serum) in the same volume as mock-infected or HIV-infected PBMC supernatants.

### Apolipoprotein E Treatment

hNP1 cells were incubated with recombinant, unlipidated human apolipoprotein E3 or E4 (Biovision Inc., Milpitas, CA) that was diluted into the culture differentiation medium to a final rApoE concentration of 3 μg/ml, which is the approximate concentration in the cerebrospinal fluid of normal subjects (Miida et al. 1999). Both recombinant proteins were produced in *E. coli*, provided as lyophilized powders, and resuspended in water to the desired concentration, then aliquoted and stored at -20 °C. The rApoE was replenished every three days, simultaneously with change of culture medium and the replenishment of HIV or mock supernatant aliquots.

### Apolipoprotein E Immunoblotting

The total protein content of whole cell lysates from cell cultures was determined by BCA protein assay (Pierce, Rockford, IL) of culture lysates; between 5 and 10 μg total protein was loaded onto 10% polyacrylamide gels. Quantitative immunoblotting (McCarthy et al. 2006) with chemiluminescent signal detection was used to assess ApoE expression. ApoE was detected with a 1:200 dilution of mouse monoclonal antibody (Abcam, Cambridge MA) and a secondary sheep anti mouse IgG diluted to 1: 2000. β actin was used as an internal control for potential loading errors in each lane; it was detected with an anti-β actin mouse monoclonal antibody (Sigma St. Louis, MO). Immunoblots were developed with SuperSignal West Femto Maximum Sensitivity Substrate (Thermo Scientific, Rockford, IL). Signals were viewed and quantitated using a Bio-Rad Chemidoc XRS+ Molecular Imager; the ApoE signal density was normalized to beta-actin. Immunoblotting detected endogenous ApoE in relatively small but stable quantities at any time along the culture timeline. Added rApoE was detected in whole cell lysates as soon as 1 h after addition, and increased through 6 h after addition. Retention of rApoE stabilized at 24 h after addition, and was still detected at 72 h (data not shown). Based on this time course, 24 h was chosen as the time lapse between the last replenishment of rApoE (day 15 in differentiation medium) and the harvest of cells for mRNA (day 16 in differentiation medium).

### RNA preparation and Gene Expression Microarray Generation

hNP1 cultures were incubated in the specified culture conditions for 16 days, with replenishment of culture medium and additives at day 15. At day 16, the incubations were terminated and cultures were lysed for preparation of RNA for gene expression determinations. RNA was extracted using the Illustra RNAspin Mini kit from GE Healthcare (Pittsburgh, PA). RNA analyses and microarray data generation were performed by the technical staff of the Oncogenomics Core Laboratory, Sylvester Comprehensive Cancer Center (SCCC), Miller School of Medicine, and the University of Miami.

Total RNA was quantified with a Nanodrop 8000 Spectrophotometer (Thermo Scientific, Wilmington), and its quality was examined with a Bioanalyzer 2100 using the RNA 6000 Nano kit (Agilent, Santa Clara, CA). Biotinylated cRNA was prepared using the Illumina TotalPrep RNA Amplification Kit (Ambion, Inc., Austin, TX) according to the manufacturer’s instructions starting with 400 ng total RNA. Successful cRNA generation was checked using the Bioanalyzer 2100. Samples were added to the Beadchip after randomization using the randomized block design to reduce batch effects. Hybridization to the Sentrix Human-HT12 Expression BeadChip (Illumina, Inc., San Diego, CA), washing and scanning were performed according to the Illumina BeadStation 500 manual (revision C). The resulting microarray data was analyzed using Illumina GenomeStudio software.

### Gene Expression Microarray Analysis

Expression data from the HumanHT-12 V4_0_R1 platform array (Illumina) were loaded on GeneSpring 12.5 GX (Agilent Technologies, Santa Clara, CA, USA). The raw data was normalized using the Quantile normalization method and log2 transformed and scaled to median of 0 for all samples. An unpaired Student’s t-test (for two class comparisons) or one-way ANOVA (for multiclass comparisons) was used to identify the significantly differentially expressed genes between the tested conditions. The nominal *p*-values obtained did not pass multiple testing corrections. Genes are considered significant if the absolute fold change differences between the conditions were ≥1.5 and *p*-value (nominal) ≤ 0.05. For ANOVA, an additional post-hoc analysis step using the tukeyHSD method was performed to derive the most significant differentially expressed genes. Functional enrichment analyses of differential gene expression were performed using the MetaCore on GeneGo program from Thompson Reuters.

### Gene Expression Validation by Quantitative RT-PCR

hNP1 monolayers were incubated in differentiation medium for 16 days, then washed once with phosphate buffered saline (PBS), and lysed using the Illustra RNAspin Mini kit from GE Healthcare (Pittsburgh, PA) for RNA extraction. RNA concentrations, 260/280, and 260/230 absorbance ratios were determined using a Nanodrop from Thermo Scientific (Wilmington, DE). Samples were diluted to 10 ng/μl in nuclease free water. One-step RT-PCR reactions were carried out using a iTaq Universal SYBR Green one step kit quantitative (qRT-PCR) from Bio-Rad laboratories (Hercules, CA) with 50 ng of RNA per 50 μl reaction and 400 nM of oligonucleotide primers. All primers were synthesized by Life Technologies (Carlsbad, CA). The qRT-PCR samples were run in a Stratagene Mx3005P thermocycler (Agilent technologies, Santa Clara, CA) with the following amplification conditions: 51 °C for 20 min for reverse transcription, an initial denaturing step at 95 °C for 10 min, then 40 cycles of 95 °C for 10 s and 57 °C for 30 s for annealing and elongation. Melting curves were performed with an initial denaturing step at 95 °C for 1 min and annealing step at 55 °C for 1 min, then increased to 95 °C for 30 s. Data was analyzed using the MxPro software (Agilent Technologies, Santa Clara, CA).

Target gene expression was normalized to the housekeeping control gene glyceraldehyde-3-phosphate dehydrogenase (GAPDH) amplified from the same sample. Gene expression was calculated using the ddCT method (Vandesompele et al. 2002). Three replicates of each sample were assayed, and three independent experimental replicates of each condition were used to estimate the expression of each gene. The mean of normalized expression for each gene was calculated from the three replicates. These values were then used to calculate the mean of values for each independent experimental replicate. Fold change in gene expression (rApoE added vs no ApoE, or mock vs Unt, or HIV vs Unt) was calculated as a direct ratio of mean gene expression values.

Representative genes chosen for validation by quantitative RT-PCR were those with significant changes in gene expression when comparing added rApoE vs no rApoE or when comparing culture treatments (e.g. mock-exposed vs untreated). After validation of the oligonucleotide primers designed for each representative gene, quantitative RT-PCR was performed using the same cellular RNA specimens that were used in the microarray analyses. Oligonucleotide primers were designed to include exon/exon boundaries on the RNA.

Oligonucleotide primers used for this study had the following sequences:

- ALCAM: Forward 5’GATGAGGCAGACGAGATAAG3’ Reverse 5’TCTTCCATATTACCGAGGTC3’
- TRIM48: Forward 5’CTGGAAGGCTTTTGGAGA3’ Reverse 5’GATATGACTGTTGGCTTCAT3’
- NEFM: Forward 5’CAAGATGGCTCTGGATAT3’ Reverse 5′ AATGTGCTAAATCTAGTCTC3’
- YWHAE: Forward 5’GCTTGAGATGGTTAAAGGTGG3’ Reverse 5’AAAGTAGGCAAGAATGAGCA3’
- PABPC1: Forward 5’GAACTTCTTCATATGCTCGA3’ Reverse 5’GCACAAGTTTCTTTTCATGG3’
- B2M: Forward 5’AGATAGTTAAGTGGGATCG3’ Reverse 5′ AAAGTGTAAGTGTATAAGCAT3’
- STAT1: Forward 5’CAGTAAAGTCAGAAATGTG3’ Reverse 5′ TTCATCTTGTAAATCTTCCA3’

## Results

### Characterization of hNP1 Cultures

**Fig. 1:**
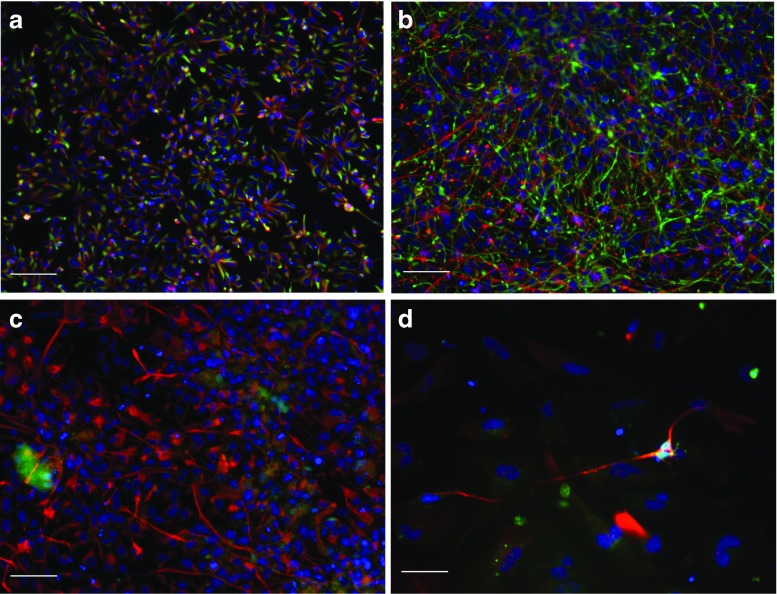
a) hNP1 cells in proliferation media for 4 days and immunostained with rabbit anti-beta-3-tubulin (1:1000) (*red*) for the neuronal microtubule antigen, and mouse anti-nestin (1:200) (*green*), for the neuroepithelial progenitor intermediate filament protein. Scale 100 μM. b) hNP1 cells in differentiation media for 12 days immunostained for rabbit anti-beta-III-tubulin (1:1000) (red) and mouse anti-nestin (1:200) (*green*). Scale 100 μM. hNP1 in differentiation media for 7 days immunostained with c) rabbit anti-NFL (1:200) (*red*) for the post-mitotic neurofilament light protein (Scale 100 μM) and d) dually stained for rabbit anti-NFL (1:200) (*red*) and mouse anti-Hu (1:200) (*green*), for the nuclear neuronal protein Hu. Scale 50 μM

Human hNP1 cells are a human neural progenitor population, derived from human embryonic stem cell line WA09. We determined their APOE genotype to be APOE3/3 (Materials and Methods). Undifferentiated hNP1 cells grown in progenitor medium expressed markers of neuronal progenitor cells, including nestin and β-III-tubulin (Fig. 1a). With directed differentiation using defined culture medium, the hNP1 cells generated a mixed population of post-mitotic neurons and neuronal lineage progenitors (Ji et al. 1998; Dhara et al. 2008). Cells differentiated for 12 or more days in differentiation medium expressed post-mitotic neurofilament antigens (Fig. 1c), Hu neuronal nuclear antigen (Fig. 1d), and β-III-tubulin along with some nestin (Fig. 1b). The principal intermediate filament protein of astrocytes, glial fibrillary acidic protein (GFAP), was not detected, either as mRNA or protein, in either undifferentiated or differentiated cells.

### Apolipoprotein E Uptake by hNP1 cells

ApoE was added to the cultures as a recombinant, unlipidated form of the protein (rApoE). This approach was preferable to other possible methods of introducing ApoE into hNP1 cells, e.g. transfection or transduction with an ApoE-coding viral vector. In vivo ApoE is not produced significantly by neurons, but by astrocytes; thus addition of exogenous rApoE better mimics the conditions found in the CNS. Introduction of ApoE-expressing vectors during HIV exposure might change the expression of viral genes unrelated to ApoE effects. Moreover, unlipidated ApoE binds to Low Density Lipoprotein Receptor-Related Protein 1 (LRP1) with higher avidity than Low Density Lipoprotein Receptor (LDLR), which is more prominently expressed in glia (Rebeck et al. 1993; Narita et al. 2002; Rapp et al. 2006), while LRP1 is expressed in neurons more than LDLR.

**Fig. 2:**
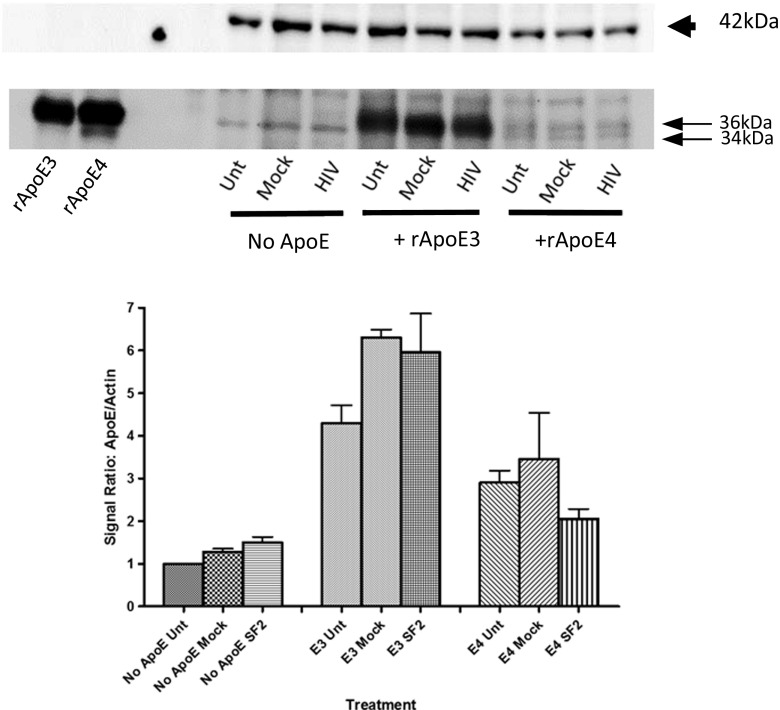
Detection of ApoE isoforms in differentiating hNP1 cells. Differentiating hNP1 cells were exposed to medium alone (Unt), mock-infected PBMC supernatants (Mock), and HIV-infected PBMC supernatants (HIV), and to rApoE3 or rApoE4 at 3 μg/ml, as described in Materials and Methods. At day 16 of differentiation, and 24 h after replenishment of rApoE in the cultures, cells were lysed for quantitative immunoblotting to detect ApoE. Lower blot depicts representative endogenous ApoE detected in cell culture lysates with molecular weight of 34 kDa, and rApoE with molecular weight of 36 kDa. Upper blot corresponds to beta-actin used as an internal control. For comparison, recombinant ApoE3 and ApoE4 were loaded directly on the gel at a 3 ng per lane, as shown. Bar graphs present ApoE signal normalized to beta-actin; each bar represents the mean of two experiments

The hNP1-derived neurons expressed endogenous ApoE, detected as a single 34 kDa band on immunoblots prepared from untreated or supernatant-exposed cultures harvested after 16 days in differentiation medium (Fig. 2). On the same immunoblots, each of the recombinant ApoE isoform preparations migrated as a predominant 36 kDa band and secondary 34 kDa band, best distinguished with rApoE4 (Fig. 2), and co-migrating with the endogenous ApoE band detected in the hNP1 cells. When hNP1-derived neurons were differentiated with added rApoE3 or rApoE4 isoforms, immunoblotting detected 36 kDa and 34 kDa bands corresponding to both rApoE and endogenous ApoE polypeptides, with a much greater ApoE signal in immunoblots from cells treated with rApoE3 compared to cells treated with rApoE4 or no added rApoE. This differential accumulation of rApoE3 in hNP1-derived neurons is consistent with previous studies showing differential retention of ApoE3 in neurons, which was attributed to effects mediated by cell surface heparin sulfate proteoglycans (Nathan et al. 1995; Ji et al. 1998).

Immunoblotting demonstrated that rApoE3 and rApoE4 isoforms are bound and retained by the differentiating hNP1 neurons. Uptake of exogenous rApoE isoform E4 would effectively change the apolipoprotein E “phenotype” of the hNP1 cells from the inherent ApoE3 to mixed ApoE3 and ApoE4. Thus, this neuronal culture model can address: 1) the effect of ApoE4 vs ApoE3 on maturing neuronal gene expression, which for the purpose of this study will be referred as the ApoE phenotype effect; and 2) the differential effect of mock- or HIV-exposure on gene expression in these maturing neurons within a given ApoE phenotype (Supplementary Fig. 1).

### Gene Expression Profiles of Differentiating hNP1 Cells Exposed to rApoE: ANOVA Analysis

Nearly 48,000 probes were tested in the Illumina HT12 microarray chip. One-way ANOVA was used for multi-class comparisons. The first one-way ANOVA comparison (Table 1) showed that the ApoE phenotype differentially affected a similar number of total genes with any culture treatment (3904 for Unt, 4078 for Mock and 4070 for HIV). When tukeyHSD post-hoc pairwise comparisons were included in the ANOVA (Table 1), the highest numbers of genes were differentially expressed with rApo4 treatment rather than rApoE3 when each is referenced to no added rApoE, or when rApoE3 is compared to rApoE4. In the pairwise comparison of rApoE3 vs rApoE4, there was an increase in the number of genes differentially expressed with mock-exposed or HIV-exposed culture treatments (1158 for Unt, 1514 for Mock and 1532 for HIV).

**Table 1:** Number of genes differentially affected by rApoE treatment (ANOVA, *p* ≤ 0.05)

Culture treatment	tukeyHSD post-hoc pairwise comparison			
	Total genes	E3 vs E4	E3 vs no rApoE	E4 vs no rApoE
Unt	3904	1158	1741	2218
Mock	4078	1514	1754	2170
HIV	4070	1532	1558	2182

One-way ANOVA was used to assess the differential expression of Illumina HT12 gene probes as a function of rApoE treatment. Total genes represent the number of gene probes that are differentially affected among the three rApoE treatments (rApoE3, rApoE4, no added rApoE). This analysis was conducted for each culture treatment. TukeyHSD post-hoc pairwise comparisons of the number of genes differentially expressed between pairs of treatments are listed; there was little difference in the number of genes differentially expressed between rApoE4 and no added rApoE among the three culture treatments

The second one-way ANOVA (Table 2) showed that about 25% fewer total genes were differentially affected by culture treatment in the presence of either rApoE compared to no added rApoE. However, there was no significant difference in the total genes when comparing rApoE3 vs rApoE4. The tukeyHSD post-hoc pairwise comparisons showed the highest number of genes in the comparisons between mock-exposed vs untreated or HIV-exposed vs untreated cultures.

**Table 2:** Number of genes differentially affected by culture treatment (ANOVA, *p* ≤ 0.05)

rApoE treatment	tukeyHSD post-hoc pairwise comparisons			
	Total genes	Mock vs Unt	Mock vs HIV	HIV vs Unt
No added rApoE	4226	2660	1260	1854
rApoE3	3112	1477	1260	1272
rApoE4	3198	1536	1176	1497

One-way ANOVA was used to assess the differential expression of Illumina HT12 gene probes as a function of culture treatments (untreated, mock-exposed, HIV-exposed). Total genes represent the number of gene probes that are differentially affected among the three culture treatments in cultures with no rApoE added, or when rApoE3 or rApoE4 were added. TukeyHSD post-hoc pairwise comparisons of the number of genes differentially expressed between pairs of culture treatments are listed. A similar number of genes are differentially expressed in all the ApoE phenotypes when comparing mock - vs HIV-exposed cultures

### Gene expression Profiles of Differentiating hNP1 Cells exposed to rApoE: Two-class comparisons and Fold Changes

To generate differentially expressed gene profiles for each of the rApoE isoforms in differentiating hNP1 cultures, fold change in gene expression was calculated with added rApoE vs no added rApoE for each of the three culture treatments. Significantly differentially expressed genes and their fold differences were determined by performing a 2-class comparison between cultures with vs without rApoE (Materials and Methods).

#### Effect of rApoE3

**Fig. 3:**
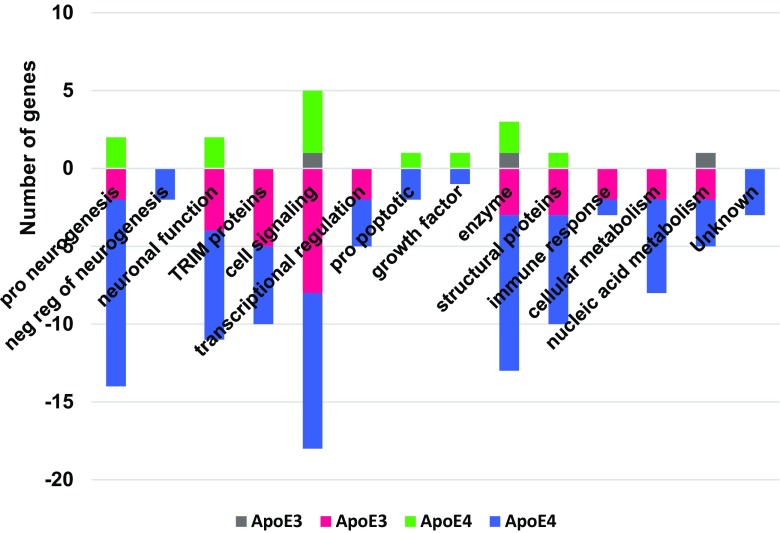
Function profiles of differentially expressed genes in hNP1 cultures as a function of added rApoE isoform. Differentially expressed genes (added rApoE3 or E4 compared to no added rApoE with an absolute FC value of ≥1.5 and *p* ≤ 0.05) were plotted according to their known biological function. Included are genes with differential expression in any culture treatment (unt or mock or HIV)

When rApoE3 was added to differentiating hNP1 cultures, a total of 36 genes were differentially expressed (rApoE3 vs no added rApoE) when including any culture treatment (Table 3). Only one gene, TRIM51, was differentially expressed in common for all three culture treatments (untreated, mock-exposed, and HIV-exposed). TRIM51, which was downregulated, belongs to the TRIM (TRIpartite interaction motif) family of genes whose products are associated with diverse cellular functions, from innate immune regulation (Versteeg et al. 2014) to autophagy (Kimura et al. 2016). The gene for Stathmin 2 (STMN2), a nervous system protein that is upregulated during differentiation (reviewed by (Chauvin and Sobel 2015)) was the only gene that was differentially expressed in common between mock- and HIV-exposed cultures. The majority of genes were differentially expressed only in mock-exposed cultures (*n* = 10) or only in untreated cultures (*n* = 20). When genes were differentially expressed in more than one culture treatment, the direction of the change was the same, i.e. upregulated or downregulated, the latter being the direction for the majority (32) of differentially expressed genes affected by rApoE3. The genes differentially expressed by rApoE3 varied in their function (Fig. 3). Prominent were cell signaling genes (e.g. YWHAE (the only upregulated gene with this function), SOCS2, TNFRSF12A), and seven downregulated genes associated with immune response or immune modulation. These included five TRIM gene family members (TRIM51, TRIM 48, TRIM49D1, TRIM49C, TRIM49D2), and two other genes associated with immunity (SPP1 and CLEC2D). The complete list of genes differentially expressed in the presence of rApoE3, along with their specific functions, is in Supplementary Table 1.

**Table 3:** Genes differentially regulated by rApoE3, pairwise comparisons

Genes differentially regulated in common between [HIV + rAPOE3 vs HIV], [Mock + rApoE3 VS Mock] and [Unt + rApoE3 vs Unt] *n* = 1					
Gene ID	Symbol	Gene Name	FC HIV + E3 vs HIV	FC Mock + E3 vs Mock	FC Unt + E3 vs Unt
84767	SPRYD5 (AKA TRIM51)	Tripartite motif-containing protein 51	-1.7	-1.9	-1.51
Genes differentially regulated in common between [Mock + rApoE3 vs Mock] and [Unt + ApoE3 vs Unt] *n* = 2					
Gene ID	Symbol	Gene Name		FC Mock + E3 vs Mock	FC Unt + E3 vs Unt
7531	YWHAE	Tyrosine 3-Monooxygenase/Tryptophan 5-Monooxygenase Activation Protein, Epsilon		1.6	1.61
79097	TRIM48	Tripartite motif-containing protein 48		-1.8	-1.61
Genes differentially regulated in common between [HIV + rApoE3 vs HIV] and [Mock + rApoE3 vs Mock] *n* = 1					
Gene ID	Symbol	Gene Name		FC HIV + E3 vs HIV	FC Mock + E3 vs Mock
11075	STMN2	Stathmin 2		-1.54	-1.68
Genes differentially regulated in common between [HIV + rApoE3 vs HIV] and [Unt + Apo3 vs Unt] *n* = 2					
Gene ID	Symbol	Gene Name		FC HIV + E3 vs HIV	FC Unt + E3 vs Unt
6696	SPP1	Secreted phosphoprotein 1		-1.57	-1.61
4023	LPL	Lipoprotein Lipase		-1.61	-1.61
Genes differentially regulated only by Mock + rApoE3 vs Mock *n* = 10					
Gene ID	Symbol	Gene Name		FC Mock + E3 vs Mock	
26986	PABPC1	Poly(A) binding protein cytoplasmic 1		1.65	
64710	LOC100130123 AKA NUCKS1	Nuclear casein kinase and cyclin dependent kinase substrate 1		1.54	
729384	LOC729384 AKA TRIM49D2	Tripartite motif containing 49D2		-1.5	
8835	SOCS2	Suppressor of cytokine signaling 2		-1.5	
642612	LOC653111 AKA TRIM49C	Tripartite motif containing 49C		-1.52	
6133	RPL9	Ribosomal protein L9		-1.53	
29121	CLEC2D	C-type lectin domain family 2 member D		-1.54	
6860	SYT4	Synaptotagmin 4		-1.55	
80333	KCNIP4	Potassium voltage-gated channel interacting protein 4		-1.61	
399939	LOC399939 AKA TRIM49D1	Tripartite motif containing 49D1		-1.8	
Genes differentially regulated only in Unt + rApoE3 vs Unt *n* = 20					
Gene ID	Symbol	Gene Name		FC Unt + E3 vs Unt	
1592	CYP26A1	Cytochrome P450 family 26 subfamily A member 1		1.58	
56884	FSTL5	Follistatin like 5		-1.5	
25897	RNF19A	Ring finger protein 19A, RBR E3 ubiquitin protein ligase		-1.5	
1848	DUSP6	Dual specificity phosphatase 6		-1.51	
5358	PLS3	Plastin 3		-1.52	
51330	TNFRSF12A	Tumor necrosis factor receptor superfamily member 12A		-1.52	
26018	LRIG1	Leucine rich repeats and immunoglobulin like domains 1		-1.54	
23406	COTL1	Coactosin like F-actin binding protein 1		-1.55	
214	ALCAM	Activated leukocyte cell adhesion molecule		-1.56	
1959	EGR2	Early growth response 2		-1.56	
7980	TFPI2	Tissue factor pathway inhibitor 2		-1.56	
115207	KCTD12	Potassium channel tetramerization domain containing 12		-1.6	
8553	BHLHB2	Basic Helix-Loop-Helix Family Member E40		-1.62	
8459	TPST2	Tyrosylprotein sulfotransferase 2		-1.62	
7857	SCG2	Secretogranin II		-1.63	
800	CALD1	Caldesmon 1		-1.64	
1052	CEBPD	CCAAT/enhancer binding protein delta		-1.64	
23180	RFTN1	Raftlin, lipid raft linker 1		-1.66	
8870	IER3	Immediate early response 3		-1.7	
133	ADM	Adrenomedullin		-1.9	
	AKA BHLHE40				

Genes differentially regulated by HIV+ rApoE3 vs HIV *n* = 0

Differential gene expression was determined from comparisons between added rApoE3 and no ApoE in the three culture treatments. FC values were calculated as described in Materials and Methods, and FC are listed if they had an absolute value of 1.5 or more and were significant with a nominal *p* value of ≤0.05. Pseudogenes or discontinued probes were removed from this list. For genes with multiple probes, the FC value listed is the mean of all absolute FC values greater or equal to 1.5

#### Effect of rApoE4

When rApoE4 was added to differentiating hNP1 cultures, a total of 85 genes were differentially expressed (rApoE4 vs no added rApoE), when including any culture treatment (Table 4). Of the 85 genes differentially affected by rApoE4, only 13 were upregulated, while the majority (72) were downregulated. The genes differentially affected by rApoE4 in any culture treatment represented functions including pro-neurogenesis (*n* = 12), neuronal function (*n* = 7), cell signaling (*n* = 10) and enzymatic activities (*n* = 10), which were predominantly downregulated (Fig. 3). Sixteen genes were differentially expressed in all culture treatments (untreated, mock-exposed, and HIV-exposed); 4 were upregulated and 12 downregulated. Among the downregulated gene functions were cell signaling (*n* = 3, LRIG1, IER3, RFTN1), neurogenesis (*n* = 2, STMN2, MAB21L2), and neuronal function (*n* = 2, SCG2, ALCAM). Twenty genes were differentially expressed in common between mock- and HIV-exposed cultures. Twelve genes were differentially expressed only in HIV-treated cultures; all but one of these were downregulated. Eighteen genes were differentially expressed only in untreated cultures, and 8 genes only in mock-exposed cultures. The complete list of genes differentially expressed in the presence rApoE4, along with their specific functions, is in Supplementary Table 2.

**Table 4:** Genes differentially regulated by rApoE4. Pairwise comparisons

Genes differentially regulated in common between [HIV + rApoE4 vs HIV], [Mock + rApoE4 vs Mock] and [Unt + rApoE4 vs Unt] *n* = 16					
Gene ID	Symbol	Gene Name	FC HIV + E4 vs HIV	FC Mock + E4 vs Mock	FC Unt + E4 vs Unt
22915	MMRN1	Multimerin 1	1.83	1.88	1.75
83648	C8ORF13	Family with sequence similarity 167 member A (FAM167A)	1.69	1.88	1.71
1381	CRABP1	Retinoic acid binding protein	1.60	1.61	1.89
1949	EFNB3	Ephrin B3	1.51	1.55	1.53
26018	LRIG1	leucine rich repeats and immunoglobulin like domains 1	-1.58	-1.53	-1.58
8870	IER3	immediate early response 3	-1.62	-1.52	-1.7
7857	SCG2	secretogranin II	-1.68	-1.51	-1.77
800	CALD1	caldesmon 1	-1.71	-1.53	-1.57
10586	MAB21L2	mab-21 like 2	-1.73	1.04	-1.73
214	ALCAM	Activated leukocyte cell adhesion molecule	-1.74	-1.56	-1.94
133	ADM	Adrenomedullin	-1.78	-1.55	-2.1
4023	LPL	lipoprotein lipase	-1.81	-1.61	-2.03
23180	RFTN1	raftlin, lipid raft linker 1	-1.81	-1.56	-1.86
40876	SYT4	synaptotagmin 4	-2.17	-2.37	-1.9
11075	STMN2	stathmin 2	-2.75	-2.73	-2.16
79097	TRIM48	tripartite motif containing 48	-2.91	-3.16	-1.82
Genes differentially regulated in common between [HIV + rApoE4 vs HIV] and [Mock + rApoE4 vs Mock] *n* = 20					
Gene ID	Symbol	Gene Name		FC HIV + E4 vs HIV	FC Mock + E4 vs Mock
81706	PPP1R14C	protein phosphatase 1 regulatory inhibitor subunit 14C		1.80	1.73
10371	SEMA3A	semaphorin 3A		1.50	1.55
27319	BHLHE22	Basic helix-loop-helix family member e22		-1.54	-1.61
3486	IGFBP3	insulin like growth factor binding protein 3		-1.54	-1.51
253738	EBF3	early B-cell factor 3		-1.56	-1.57
6422	SFRP1	secreted frizzled related protein 1		-1.56	-1.56
27065	D4S234E	neuron specific gene family member 1		-1.57	-1.64
9118	INA	internexin neuronal intermediate filament protein alpha		-1.58	-1.56
4741	NEFM	Neurofilament, medium polypeptide		-1.61	-1.51
1282	COL4A1	collagen type IV alpha 1 chain		-1.64	-1.52
8835	SOCS2	Suppressor of cytokine signaling activity 2		-1.66	-1.74
1641	DCX	doublecortin		-1.76	-1.83
6658	SOX3	SRY-box 3		-1.78	-1.59
729384	LOC729384	tripartite motif containing 49D2 TRIM49D2		-1.88	-2.14
63973	NEUROG2	Neurogenin 2		-1.93	-2.14
388585	HES5	hes family bHLH transcription factor 5		-1.97	-2.22
100008589	LOC100008589	RNA, 28S ribosomal 5		-2.47	1.61
642,612	LOC100134006 aka TRIM49C	tripartite motif containing 49C		-2.44	-2.63
399939	LOC399939 aka TRIM49D1	tripartite motif containing 49D1		-2.53	-2.56
84767	SPRYD5 aka TRIM51	tripartite motif-containing 51		-3.1	-3.61
Genes differentially regulated in common between [HIV + rApoE4 vs HIV] and [Unt + rApo4 vs Unt] *n* = 9					
Gene ID	Symbol	Gene Name		FC HIV + E4 vs HIV	FC Unt + E4 vs Unt
1592	CYP26A1	cytochrome P450 family 26 subfamily A member 1		1.54	1.67
6696	SPP1	secreted phosphoprotein 1		-1.51	-1.63
51330	TNFRSF12A	TNF receptor superfamily member 12A		-1.53	-1.73
1278	COL1A2	collagen type I alpha 2 chain		-1.55	-1.56
1363	CPE	carboxypeptidase E		-1.56	-1.76
56884	FSTL5	follistatin like 5		-1.59	-1.75
167681	PRSS35	protease, serine 35		-1.59	-1.91
5358	PLS3	plastin 3		-1.6	-1.64
115207	KCTD12	potassium channel tetramerization domain containing 12		-1.65	-2.07
Genes differentially regulated in common between [Mock + rApoE4 vs Mock] and [Unt + rApoE4 vs Unt] *n* = 2					
Gene ID	Symbol	Gene Name		FC Mock + E4 vs Mock	FC Unt + E4 vs Unt
7531	YWHAE	tyrosine 3-monooxygenase/tryptophan 5-monooxygenase activation protein epsilon		1.58	1.54
26986	PABPC1	poly(A) binding protein cytoplasmic 1		-1.78	-1.65
Genes differentially regulated only by [HIV+ rApoE4 vs HIV] *n* = 12					
Gene ID	Symbol	Gene Name			FC HIV + E4 vs HIV
79966	SCD5	stearoyl-CoA desaturase 5			1.7
79007	DBNDD1	dysbindin domain containing 1			-1.5
27242	TNFRSF21	TNF receptor superfamily member 21			-1.5
163	AP2B1	adaptor related protein complex 2 beta 1 subunit			-1.53
100169760	RN5S9	RNA, 5S ribosomal 9			-1.54
6513	SLC2A1	solute carrier family 2 member 1			-1.56
8148	TAF15	TATA-box binding protein associated factor 15			-1.56
389073	C2ORF80	chromosome 2 open reading frame 80			-1.66
2296	FOXC1	forkhead box C1			-1.67
8140	SLC7A5	solute carrier family 7 member 5			-1.72
72	ACTG2	actin, gamma 2, smooth muscle, enteric			-1.78
56660	KCNK12	potassium two pore domain channel subfamily K member 12			-1.8
Genes differentially regulated only by [Mock +rApoE4 vs Mock] *n* = 8					
Gene ID	Symbol	Gene Name			FC Mock + E4 vs Mock
653	BMP5	bone morphogenetic protein 5			1.59
7473	WNT3	Wnt family member 3			1.54
59	ACTA2	actin, alpha 2, smooth muscle, aorta			-1.52
4661	MYT1	myelin transcription factor 1			-1.52
55775	TDP1	tyrosyl-DNA phosphodiesterase 1			-1.56
9208	LRRFIP1	LRR binding FLII interacting protein 1			-1.57
2674	HS.388347	GDNF family receptor alpha 1			-1.61
441087	LOC441087	uncharacterized			-1.73
Genes differentially regulated only by [Unt + rApoE4 VS Unt] *n* = 18					
Gene ID	Symbol	Gene Name			FC Unt + E4 vs Unt
348	APOE	apolipoprotein E			-1.59
794	CALB2	calbindin 2			-1.58
54769	DIRAS2	DIRAS family GTPase 2			-1.52
1848	DUSP6	dual specificity phosphatase 6			-1.60
1959	EGR2	early growth response 2			-1.77
2012	EMP1	epithelial membrane protein 1			-1.90
10912	GADD45G	growth arrest and DNA damage inducible gamma			1.54
153572	IRX2	iroquois homeobox 2			1.53
85450	ITPRIP	inositol 1,4,5-trisphosphate receptor interacting protein			-1.57
3915	LAMC1	laminin subunit gamma 1			-1.53
3988	LIPA	lipase A, lysosomal acid type			-1.62
4627	MYH9	myosin, heavy chain 9, non-muscle			-1.52
10455	PECI	enoyl-CoA delta isomerase 2			-1.50
5730	PTGDS	prostaglandin D2 synthase			-1.54
5764	PTN	pleiotrophin			-1.62
25897	RNF19A	ring finger protein 19A, RBR E3 ubiquitin protein ligase			-1.74
6464	SHC1	SHC adaptor protein 1			-1.52
7980	TFPI2	tissue factor pathway inhibitor 2			-1.69

Differential gene expression was determined from comparisons between added rApoE4 and no ApoE in the three culture treatments. FC values were calculated as described in Materials and Methods, and FC are listed if they had an absolute value of 1.5 or more and were significant with a nominal *p* value of ≤0.05. Pseudogenes or discontinued probes were removed from this list. For genes with multiple probes, the FC value listed is the mean of all absolute FC values greater or equal to 1.5

The total number of genes differentially affected by rApoE4 (85) was more than double the number that were differentially affected by rApoE3 (36). However, 28 of the differentially affected genes were in common between rApoE3 and rApoE4, and 26 of these were downregulated genes associated with functions including cell signaling (*n* = 8) and neuronal function (*n* = 2). There were 57 genes that were uniquely differentially expressed in the presence of rApoE4. Of these, 13 were genes with protein products promoting neurogenesis, and 11 were downregulated. Of the eight genes that were uniquely differentially expressed in the presence of rApoE3, none were functionally related to neurogenesis, but one was associated with neuronal function (KCNIP4).

### Effect of rApoE within Culture Treatments

The rApoE4 isoform had a much greater impact than did rApoE3 when comparing differential gene expression (i.e. rApoE vs no added rApoE) within each culture treatment (Supplementary Table 3). The difference between rApoE3 and rApoE4 effects was most evident in the differential gene expression associated with mock-exposed and HIV-exposed cells.

#### Untreated

**Fig. 4:**
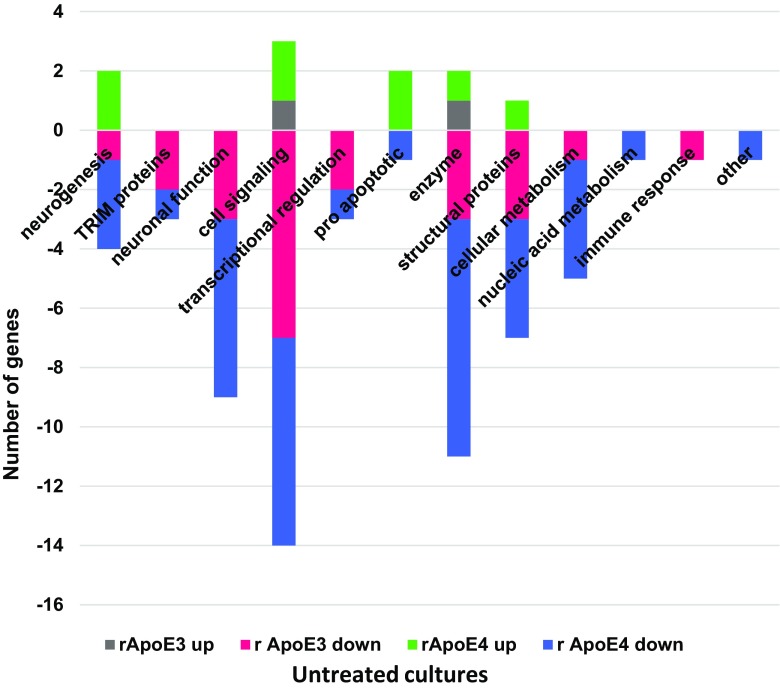
Function profiles of differentially expressed genes in untreated cultures with added rApoE. Differentially expressed genes (added rApoE3 or E4 compared to no added rApoE with an absolute FC value of ≥1.5 and *p* ≤ 0.05) were plotted according to their known biological function

In untreated cultures, 25 genes were differentially affected by rApoE3, with 23 of these being downregulated (Supplementary Table 3). Eightly percent (20/25) of the differentially affected genes were also differentially affected in the same direction by rApoE4 (Supplementary Table 2). About 1/3 of the rApoE3-affected genes in untreated cultures were associated with cell signaling functions (Fig. 4). A total of 45 genes were differentially affected by rApoE4 in untreated cultures; 18% of these (8/45) were up-regulated (Fig. 4), including 2 neurogenesis-related genes (EFNB3, IRX2) and 2 cell signaling-related genes (FAM167A, YWHAE). Among the differentially affected genes unique to untreated cultures, rApoE3 and rApoE4 shared 4 genes: DUSP6, EGR2, RNF19A, TFPI2 (see Tables 3 and 4). Shared gene RNF19A is Ring Finger Protein 19A, RBR E3 Ubiquitin Protein Ligase. This protein has a potential neuroprotective role, by binding to pathogenic forms of superoxide dismutase (SOD1) variants and targeting them for proteasomal degradation (Niwa et al. 2002). In cells treated with rApoE, this gene is downregulated (FC −1.50 with rApoE3, FC −1.74 with rApoE4).

#### Mock

**Fig. 5:**
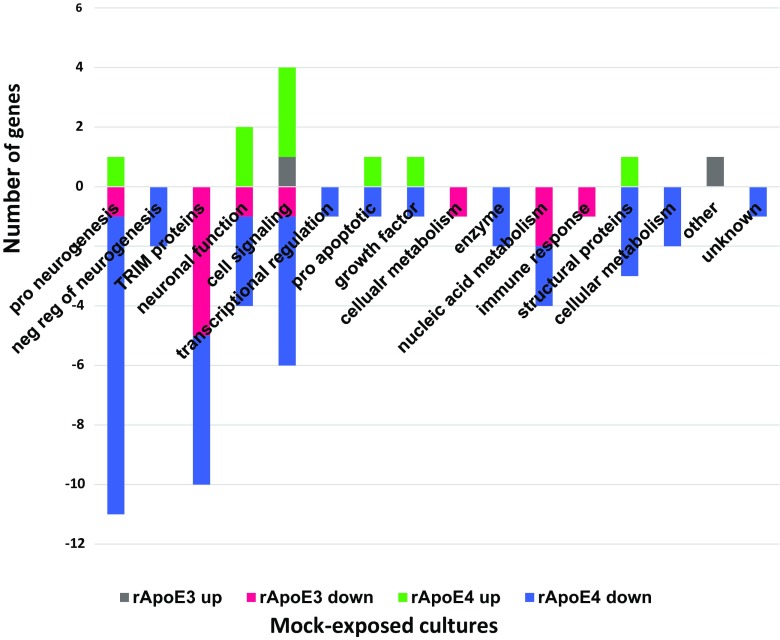
Function profiles of differentially expressed genes in mock-exposed cultures with added rApoE. Differentially expressed genes (added rApoE3 or E4 compared to no added rApoE with an absolute FC value of ≥1.5 and *p* ≤ 0.05) were plotted according to their known biological function

In mock-exposed cultures, 14 genes were differentially affected by rApoE3, with 12 of these being downregulated (Supplementary Table 3). Ten of the 14 genes differentially affected by rApoE3 were also differentially affected in the same direction by rApoE4. About 40% of the rApoE3-affected genes in mock-exposed cultures were TRIM proteins (Fig. 5). A total of 47 genes were differentially affected by rApoE4 in mock-exposed cultures; 19% of these (9/47) were up-regulated (Fig. 5), including 3 cell signaling-related genes (FAM167A, YWHAE, WNT3). Among the differentially affected genes unique to mock-exposed cultures, rApoE3 and rApoE4 did not share any genes.

#### HIV

**Fig. 6:**
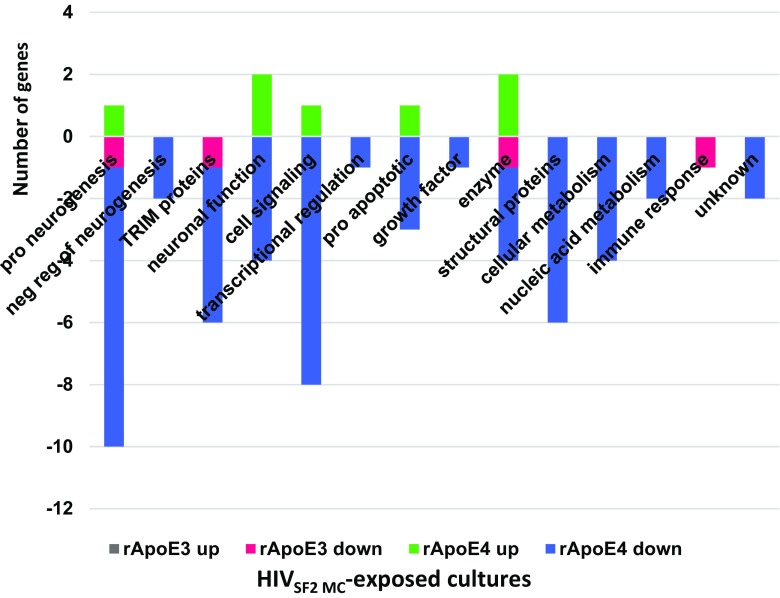
Function profiles of differentially expressed genes in HIV-exposed cultures with added rApoE. Differentially expressed genes (added rApoE3 or E4 compared to no added rApoE with an absolute FC value of ≥1.5 and *p* ≤ 0.05) were plotted according to their known biological function

In HIV-exposed cultures, rApoE4 had an almost exclusive effect on gene expression, as compared to rApoE3 (Supplementary Table 3, Tables 3, 4). Only 4 genes were differentially affected by rApoE3 (Supplementary Table 3, Table 3, Fig. 6); all of these were downregulated, and all 4 genes were also differentially affected in the same direction by rApoE4 (Supplementary Table 3, Table 4). A total of 57 genes were differentially affected by rApoE4 in HIV-exposed cultures, which was the highest number of genes affected by rApoE in any of the culture treatments. Twelve percent of these (7/57) were up-regulated, including 2 neuronal function genes (PPP1R14C, SEMA3A) and two genes genes coding for enzymes (CYP26A1, SCD5) (Fig. 6). Twenty one percent (12/57) of the genes differentially affected by rApoE4 in HIV-exposed cultures were unique to HIV-exposure; these 12 genes represented a variety of cell functions, and all but one were downregulated (Table 4, Fig. 6).

### Effect of Mock- or HIV-Exposure on Differential Gene Expression: Pairwise Comparisons

To look for genes that are impacted by culture treatment but not by ApoE phenotype, gene expression in mock-exposed or HIV-exposed cultures was compared by Student’s t-test to that of the same gene in untreated cultures. Then differential gene expression was calculated as a FC, which represents the ratio of a gene’s expression in mock-exposed vs untreated or HIV-exposed vs untreated hNP1 cultures. Genes with FC having nominal *p* value ≤0.5 and absolute FC value ≥1.5 were identified, and then categorized for function.

Among all ApoE phenotypes, including hNP1 cultures without any added rApoE, 4 genes are upregulated in common by mock supernatant exposure as compared to untreated (B2M, STAT1, TAP1, KCNIP4), and 1 gene is upregulated in common by HIV exposure as compared to untreated (KCNIP4). Thus, 4 genes are upregulated in hNP1-derived neurons exposed to mock- or HIV-supernatants, regardless of ApoE. The ubiquitous KCNIP4 gene encodes a voltage-gated potassium (Kv) channel-interacting protein which regulates neuronal excitability in response to changes in intracellular calcium. Two of these consistently upregulated genes, B2M (beta-2-microglobulin) and STAT1, were significantly upregulated (*p* ≤ 0.05) in HIV-exposed mixed astrocyte and neuron cultures differentiated from human NEP, regardless of ApoE genotype (Geffin et al. 2013). Both B2M and STAT1 are reportedly upregulated in transcriptomes from NeuroAIDS brain (Winkler et al. 2012). Thus, these hNP1-derived neurons identify at least 2 common neuronal-specific gene responses to HIV exposure.

### Functional Enrichment Analyses

For these analyses, the input gene sets were the differentially expressed genes derived from the pairwise comparison of gene expression with added rApoE vs no added rApoE in untreated, mock-exposed, or HIV-exposed cultures.

**Fig. 7:**
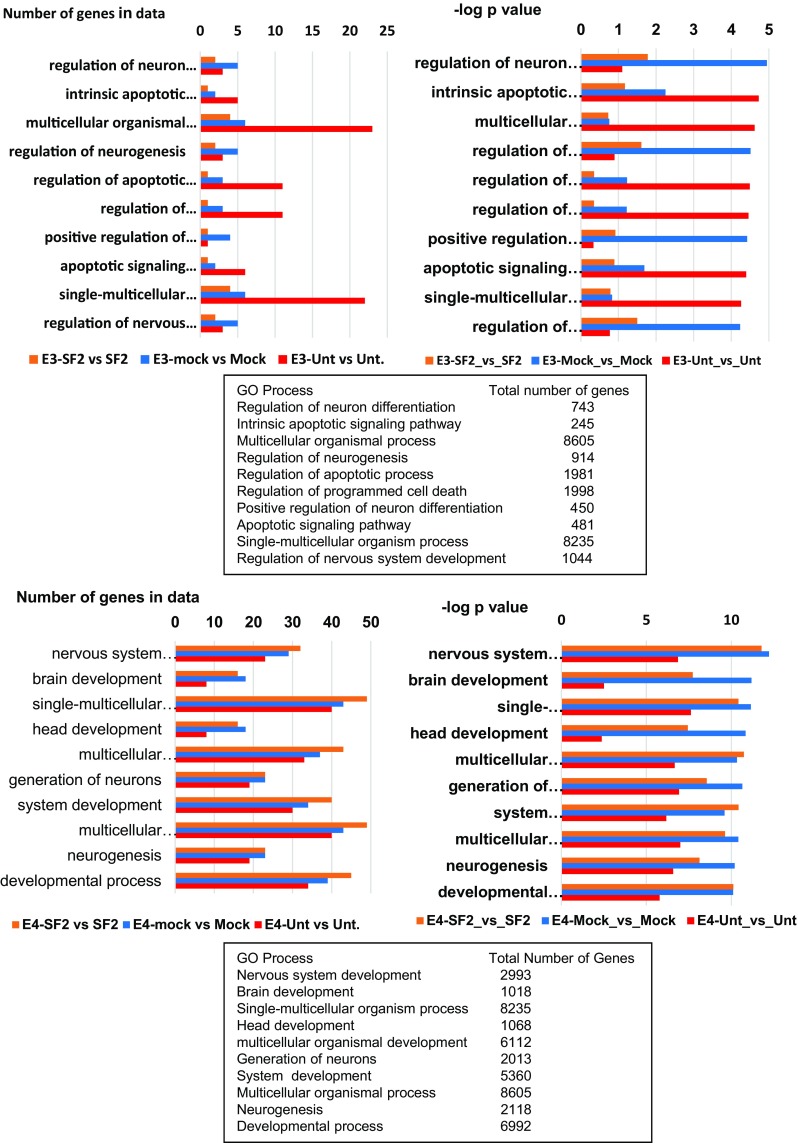
GO processes enriched by added rApoE3 (upper panel) or rApoE4 (lower panel) when comparing untreated, mock and HIV-treated cultures. The input gene sets were the differentially expressed genes derived from the pairwise comparison of gene expression with added rApoE vs no added rApoE in untreated, mock-exposed, or HIV-exposed cultures; the differentially expressed genes had absolute FC values of 1.5 or more, and nominal *p* value of ≤0.05. Top 10 GO processes are listed with the number of genes participating in the process and the –log *p* value of their significance. The names of the processes and the total number of genes in the process are listed in the text boxes below each panel

Enrichment pathway analysis found no enriched canonical pathways for genes differentially affected by rApoE3 with absolute FC value of 1.5 or more, regardless of culture treatment. Enrichment analysis for GO (Gene Ontology) cellular processes for the same rApoE3-related input gene sets revealed a small number of genes in each GO cellular process that was found to be enriched, as compared to the total number of genes participating in the pathway (Fig. 7). The top 10 most significant GO cellular processes affected by rApoE3 include regulation of neuron differentiation, and intrinsic apoptotic signaling pathway. However, the most significant *p* value was 1.131 × 10^−05^ FDR 1.234 × 10^−03^ for the top GO process (regulation of neuron differentiation) comparing mock cultures with added ApoE3 vs mock cultures without added ApoE. Four of the top 10 GO processes in this analysis were related to neuronal lineage cells and were most significantly enriched by rApoE3 in mock-exposed cultures: regulation of neuron differentiation, regulation of neurogenesis, positive regulation of neuron differentiation, regulation of nervous system development. A list of GO processes affected by rApoE3 as well as the genes enriched in these processes is located in Supplementary Table 4.

Functional enrichment pathway analysis identified a number of canonical pathways for genes that were significantly differentially affected by rApoE4 with absolute FC of 1.5 or more. Again, a small number of genes significantly enriched the identified pathways for each of the culture treatments, and *p* values ranged from the order of 10^−1^ to 10^−5^ (data not shown). Only one of the top 10 enriched pathways involved neuronal function. However, when the GO cellular processes were examined with the same input gene sets, a larger number of genes differentially affected by rApoE4 were in data for each process (Fig. 7). Of the top 10 GO cellular processes enriched by rApoE4-affected genes in all three culture treatments, at least 5 directly involve neuronal development. These included nervous system development and brain development (the top 2 processes) and head development, generation of neurons, and neurogenesis. The *p* value for the most significant process (nervous system development) was 6.13 × 10^−13^, FDR 1.73 × 10^−9^ found with the differentially affected genes in mock-exposed cultures. In all of the top 10 GO cellular processes, the *p* values were consistently more significant with genes differentially affected by rApoE4 in mock-exposed and HIV-exposed cultures, while *p* values were less significant with genes in untreated cultures (Fig. 7). The genes actively participating in these neuronal-related processes were most often downregulated, and these downregulated genes have functional significance for neurogenesis and neuronal growth (Supplementary Table 5). For example, in HIV-exposed cultures, 36 genes were found to be enriched in the “nervous system development” GO process. Of these 36 genes, at least 20 were downregulated (Supplementary Table 5). These genes include SOCS2, Adrenomedullin, STMN2, Synaptotagmin, Neurofilament-M, Doublecortin, and Neurogenin2, among others. The functional enrichment analysis by GO process yielded results that are consistent with the results obtained from gene expression profiles (Fig. 3), i.e. neurogenesis and neuronal-related genes that are differentially affected by rApoE4 tend to be downregulated. A detailed list of GO processes enriched by rApoE4 is located in Supplementary Table 5.

**Fig. 8:**
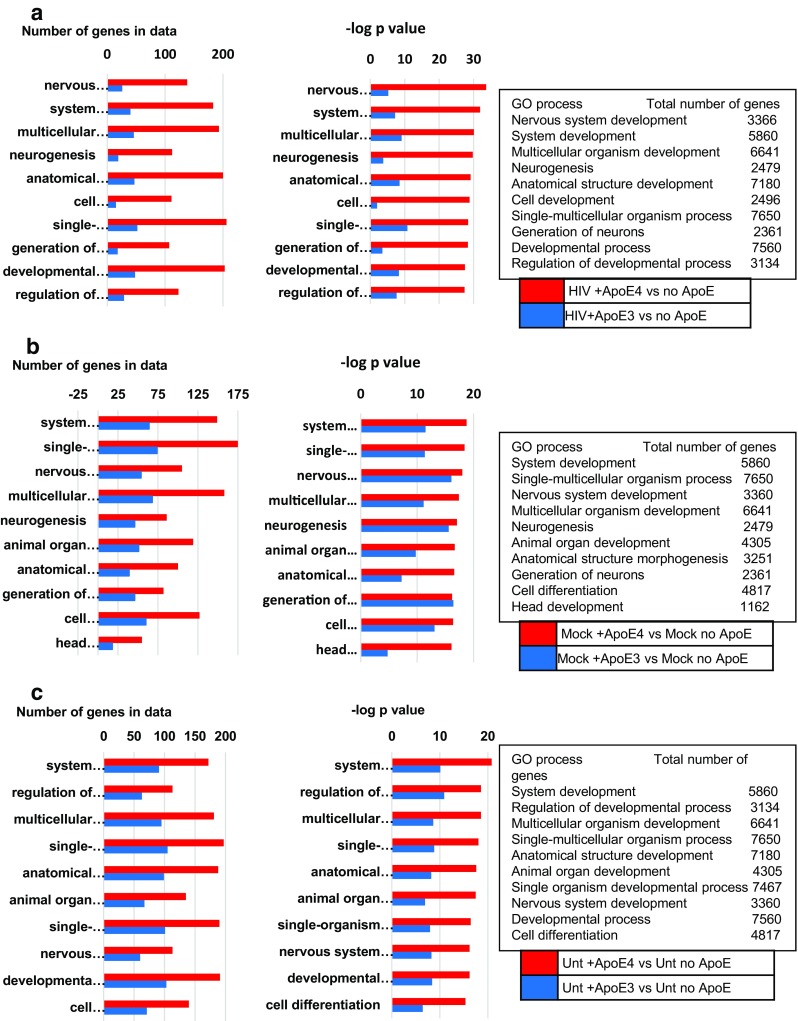
GO processes enriched when comparing the effect of rApoE3 or rApoE4 within each culture treatment. The input gene sets were the differentially expressed genes derived from the pairwise comparison of gene expression with added rApoE vs no added rApoE in HIV-exposed (Fig. 8a), mock-exposed (Fig. 8b), or untreated (Fig. 8c) cultures with absolute FC values of 1.2 or more, and nominal *p* value of ≤0.05. Top 10 GO processes are listed with the number of genes participating in the process and the –log *p* value of their significance. The names of the processes and the total number of genes in the process are listed in the text boxes to the right of each panel

Parallel functional enrichment analyses were also performed to compare the effect that rApoE4 vs rApoE3 treatment have on the enrichment of GO processes in the context of the individual culture treatment. For these comparisons, the input gene sets were the differentially expressed genes derived by the pairwise comparison of rApoE (E3 or E4) vs no added rApoE in a single culture treatment. The selected genes had an absolute FC value of 1.2 or more and nominal *p* values of less than 0.05. Gene Ontology (GO process) analyses revealed significant differences in the way the two rApoE isoforms affect the functional enrichment by GO process within each culture treatment (Figs. 8a-c). In general, for all culture treatments, a larger number of genes enriched the top ten GO processes with rApoE4 treatment. This difference between rApoE4 and rApoE3 in the number of genes affecting the GO processes was more pronounced for cultures exposed to HIV (Fig. 8a). Approximately 4–7 fold more genes affected the top 10 GO processes when rApoE4 was added than rApoE3. For mock-exposed and untreated cultures, the differences between the two rApoE isoforms was approximately two-fold (Figs. 8b, c). The *p* values for all three culture treatments were very significant (between 2 × 10^−34^ to 4 × 10^−16^), but the lowest (most significant) were found for the enrichment GO processes related to HIV exposure and addition of rApoE4. For the three culture treatments, nervous system development was consistently among the top ten GO processes. More GO processes related to neuronal development, including neurogenesis and generation of neurons, were among the top 10 GO processes detected for the mock-exposed and HIV-exposed cultures, but not for untreated cultures. Other processes not specifically related to neurogenesis were also affected preferentially by rApoE4 in all three culture treatments, processes such as system development, single multicellular organism process, and multicellular organism development. Among the 26 genes affecting the top GO process (nervous system development) when rApoE3 is added in the context of HIV exposure, 21 are also found among the 138 genes enriching this GO process when ApoE4 is added, and the direction in which these common genes are differentially affected is also the same. Of the remaining 117 genes that affect the nervous system development GO processes and that are unique to ApoE4, the majority (approximately 75%) are downregulated. A list comparing GO process genes enriched by rApoE3 vs rApoE4 in the different culture treatments is located in Supplementary Table 6.

### Confirmatory RT PCR of Differentially Regulated Genes

Quantitative RT PCR was used to independently confirm the differences in gene expression obtained with microarray analysis of hNP1 cultures. Representative genes were selected to validate changes in gene expression as a consequence of rApoE addition to the cultures, or as a consequence of culture treatment with mock-infected or HIV-infected PBMC supernatants. Representative genes, reflecting the differential effect of rApoE3, rApoE4, or both, included: PABPC1, which encodes a protein involved in RNA metabolism; ALCAM, important for neurite extension; TRIM48, one of the TRIM family of proteins; and neurofilament medium polypeptide (NEFM), a post-mitotic intermediate filament protein of neurons (Table 5). Representative genes reflecting the effects of mock- or HIV exposure on differentiating hNP1 cells regardless of ApoE phenotype included: beta-2-microglobulin (B2M), and STAT1 (Table 6). Results obtained by the two methods, expressed as fold change in gene expression (Tables 5 and 6), were comparable, and indicate a close agreement between gene expression determined by microarray and by RT-PCR assays.

**Table 5:** FC values obtained by RT PCR versus gene expression microarray for genes differentially expressed with rApoE treatment

		RT PCR	MICROARRAY
PABPC1	Mock E3	-1.18	-1.32
	Mock E4	-1.34	-1.42
	Unt E4	-1.10	-1.34
ALCAM	Unt E3	-1.13	-1.56
	HIV E4	-1.43	-1.74
	Mock E4	-1.33	-1.56
	Unt E4	-1.37	-1.95
YWHAE	Mock E3	-1.14	1.38
	Unt E3	1.34	1.22
	Mock E4	-1.10	1.26
	Unt E4	1.22	1.54
TRIM48	Mock E3	-3.53	-1.81
	Unt E3	-1.99	-1.61
	HIV E4	-3.18	-2.91
	Mock E4	-4.25	-3.16
	Unt E4	-1.72	-1.82
NEFM	HIV E4	-2.00	-1.62
	Mock E4	-1.61	-1.52

Genes found to be differentially expressed with rApoE treatment as determined by gene expression microarray were then tested for differential expression by RT PCR of the same cellular RNA. FC values were derived from comparison of gene expression with rApoE vs no rApoE. The microarray FC value listed for PABPC1 represents the mean of 5 probes and for YWHAE is the mean of 3 probes

**Table 6:** Comparison of FC values obtained by RT PCR and by gene expression microarray

Gene	rApoE treatment	Culture treatment	RT PCR	Microarray
B2M	No rAPOE	HIV vs Unt	1.43	1.42
		Mock vs Unt	2.17	1.83
	rAPOE3 added	HIV vs Unt	2.04	1.85
		Mock vs Unt	1.91	1.90
	rAPOE4 added	HIV vs Unt	1.80	1.57
		Mock vs Unt	1.92	1.93
STAT1	No rAPOE	HIV vs Unt	1.50	1.26
		Mock vs Unt	2.10	1.52
	rAPOE3 added	HIV vs Unt	2.11	1.33
		Mock vs Unt	2.09	1.57
	rAPOE4 added	HIV vs Unt	1.76	1.30
		Mock vs Unt	1.77	1.52

Genes found to be differentially regulated by culture treatments detected using gene expression microarray were tested for their expression using RT PCR. FC values are listed for each culture treatment comparisons. FC values listed for microarray analysis for both genes are the average of more than one probe

## Discussion

In this study, mock- or HIV-exposed neuronal lineage cells were differentiated directly from the human neural progenitor cell line hNP1 in the presence of recombinant ApoE3 or ApoE4 isoforms in order to examine the effect of ApoE phenotype on neuronal gene expression. These hNP1-derived neuronal cultures lack a GFAP-expressing astrocyte sub-population, so the observed differential gene responses to HIV exposure or to ApoE isoforms represent neuronal-specific responses. The hNP1-derived neurons, with an inherent APOE3/3 genotype, produce detectable but low amounts of endogenous ApoE, characteristic of neurons. But these cells take up and retain rApoE3 or rApoE4 isoforms readily when rApoE is added to cultures in μg/ml concentrations, mimicking what neurons do in vivo when ApoE is secreted by astrocytes. The ApoE phenotype of the hNP1-derived neurons was thus affected by the added rApoE isoform, and differences in gene expression were observed accordingly, with both fold change values and functional genomics analyses. Addition of rApoE3 resulted in the differential expression of 36 genes, while addition of rApoE4 was remarkable in both the number of genes that were differentially regulated (85, more than double than rApoE3) and in the negative direction of the regulation. A suppressive effect of rApoE4 was demonstrated with downregulation of 72 of the 85 differentially expressed genes. Of these, 19 coded for proteins associated with neurogenesis and neuronal functions. Differences in gene expression between rApoE3 and rApoE4 were more pronounced in mock- or HIV-exposed compared to untreated hNP1 neurons. This suggests that mitogens or factors released by stimulated PBMC (Martinez et al. 2012) may affect the interaction between rApoE3 or rApoE4 isoforms and hNP1 neurons. However, the most striking differences in gene expression between rApoE3 and rApoE4 were observed in HIV-exposed hNP1 neurons (4 genes differentially regulated by rApoE3 compared to 57 genes by rApoE4). Enrichment analysis of the differentially expressed genes also found that the number of genes enriching the Gene Ontology (GO) processes was highest in cultures exposed to HIV and with added rApoE4 compared to added rApoE3. For each of the culture treatments (untreated, mock-exposed, HIV-exposed), both the number of genes enriching the GO cellular processes and the *p* value of the effect on the biological process were more significant for cultures with rApoE4 than for cultures with rApoE3. These differential effects suggest an additive or synergistic interaction between ApoE4 and HIV.

Both this study and our previous study (Geffin et al. 2013) have endorsed a suppressive effect of ApoE4 on neural gene expression, particularly in HIV-exposed cells. In our previous study, human fetal NEP-derived cultures of mature astrocytes and post-mitotic neurons with the APOE3/3 genotype upregulated innate immune response genes when exposed to HIV. Most of these genes were interferon-related. However, HIV-exposed cultures harboring the APOE3/4 genotype had diminished innate immune gene responses, with at least 50% fewer genes enriched in the top 10 GO cellular processes. Given the known immune reactive capacity of astroglia (Liddelow and Hoyer 2016), it is likely that the astrocyte sub-population with the APOE3/4 genotype was the predominant source of the differentially suppressed immune-related gene responses. The astrocytes would also be the principal source of secreted ApoE3 or ApoE4 that binds to specific neuronal surface receptors. In the current study, the hNP1 cultures lack GFAP-expressing astrocytes, and the source of “secreted” ApoE is added exogenous rApoE. When rApoE4 was added to hNP1 neurons, the ApoE phenotype was effectively changed from the inherent ApoE3 to predominantly ApoE4. The greatest impact of the change to an ApoE4 phenotype was observed in the HIV-exposed cultures (Fig. 8a), where most of the genes enriching the GO cellular processes were downregulated. Functional genomics analysis also highlighted the suppressive impact of ApoE4 on the expression of numerous genes associated with neurogenesis, neuronal structure or function, or immune responses. Five genes that were uniquely downregulated by rApoE4 code for transcriptional factors that promote neurogenesis (EBF3, MYT1, FOXC1, NEUROG2, BHLHE22). Other downregulated genes code for structural proteins characteristic of mature neurons (DCX, NEFM, INA), growth factors (PTN) and neurotrophic factors (HS.388347).

Apolipoprotein E, particularly the APOE4 allele or the ApoE4 protein isoform, is one of the most significant of known host factors influencing neurodegenerative or inflammatory disorders (Frank et al. 2002; Jahanshad et al., 2012), particularly in older affected individuals (Pomara et al. 2008; Mukerji et al. 2016; Wendelken et al. 2016). Multiple experimental reports have expanded on the observed neurological consequences of harboring the APOE4 allele or the ApoE4 isoform. In ApoE-targeted replacement (TR) mice, dendritic spine density is lower in ApoE4 than in ApoE3 and ApoE2 TR mice (Nwabuisi-Heath et al. 2014). Neurons synthesize ApoE when subjected to stress, and the results may be injurious, especially with ApoE4 (Mahley et al. 2009). After traumatic brain injury (TBI), rodents expressing the human ApoE4 protein die more often, and those who survive do worse in cognitive tests and measurements of neurite growth compared to mice expressing the human ApoE3 protein or no human ApoE (Buttini et al. 1999; Sabo et al. 2000). After TBI induced by controlled cortical impact, ApoE3 and ApoE4 transgenic mice showed differences in gene expression profiles from both cortex and hippocampus (Crawford et al. 2009). The observed differences in gene expression predicted functional consequences related to inflammatory responses, cell growth, and cell signaling, and suggested a “loss of reparative function rather than a gain of negative function” in the presence of ApoE4 (Crawford et al. 2009).

Diverse molecular mechanisms have been postulated for ApoE’s effects, ranging from direct transcriptional repression (Theendakara et al. 2016) to facilitating amyloid plaque development in damaged neurons (Bu 2009). In a study using human neuroblastoma and human glioblastoma cells, transfection with ApoE3 and ApoE4 expression vectors revealed differential and repressive effects of ApoE4 on gene transcription (Theendakara et al. 2016). Application of ChIP-seq to assess ApoE3 and ApoE4 binding on a genome-wide scale identified four genes (sirtuin1, MADD, ADNP, COMMD6) in which ApoE4 interacted with the promoter, reduced transcription, and reduced mRNA levels. This indicated a capacity for ApoE4 to function as a transcriptional repressor. None of the ApoE4-affected genes identified in that study overlapped with the differentially expressed genes in our study. This is consistent, though, with distinct differences in the cells tested (glial-derived vs neuronal lineage) and the modes of ApoE delivery. These differences lead to relevant but distinct outcomes. However, our studies have a common strategy of altering the ApoE phenotype in human neural cells in culture, and a common outcome of observing depressed differential gene expression associated with the ApoE4 phenotype compared to the ApoE3 phenotype.

The influence of ApoE4 on HAND is controversial. Recent published studies of HIV-infected cohorts found no association between the severity of cognitive impairment and APOE genotype (Morgan et al. 2013; Becker et al. 2015), and no association between APOE4 genotype and poorer neuroimaging outcomes, even when stratified by age (Cooley et al. 2016). However, other studies have associated APOE4 genotype with faster HIV-1 disease progression (Burt et al. 2008), or with decreased cognitive executive functioning in older HIV-infected individuals (Wendelken et al. 2016). Moreover, higher CSF ApoE levels reportedly associated with poorer cognitive performance in a “dose-dependent” manner in HIV-infected APOE4 carriers (Andres et al. 2010). In a study including 70 seronegative and 69 HIV seropositive subjects (Chang et al. 2011), the APOE4 allele exhibited antagonistic pleiotropy on cognition and brain volume in seronegative controls, with a positive effect on younger and negative effect on older subjects. But the younger HIV-infected APOE4 carriers showed poorer cognition and smaller brain volumes. A subsequent study of glial metabolites expanded on this APOE4 allele effect. A study of 177 individuals, including 80 HIV seropositives, found that myo-inositol levels in frontal white matter increased with age in seronegative subjects, more steeply in APOE4 carriers. But levels were elevated regardless of age in HIV-infected individuals, suggesting that persistent glial activation due to HIV infection offsets the antagonistic pleiotropy of APOE4 (Chang et al. 2014).

The findings in this study suggest there is an interaction between ApoE4 and HIV exposure in neurons. Among the three culture treatments, rApoE4 has the greatest differential effect on gene expression in HIV-exposed hNP1 neurons, and most of the differentially expressed genes are downregulated. While rApoE4 has a largely downregulatory effect on gene expression with all culture treatments, HIV exposure magnifies this effect to a higher number of genes. In that rApoE4 can enhance HIV-target cell fusion (Burt et al. 2008), hNP1 neurons treated with rApoE4 may be binding HIV_SF2 MC_ to a greater extent than neurons treated with rApoE3, thus intensifying the effects that HIV exposure has on neuronal gene expression. Addition of rApoE4 at 10μg/ml to HIV target cells has been shown to increase their susceptibility to HIV infection, while addition of rApoE3 reduces it (Burt et al. 2008). This is due to a higher frequency of HIV-target cell fusion, and occurs using either CXCR4 or CCR5-tropic virus. This suggests a direct or indirect interaction between the virus and ApoE or the ApoE receptor that is isoform sensitive. ApoE4 may increase neuronal susceptibility to toxic effects (e.g. depressed mitochondrial membrane potential) after exposure to HIV-1 tat or gp120 proteins (Turchan-Cholewo et al. 2006). Indeed, tat protein, binds to neuronal LRP1, which can inhibit uptake and clearance of ApoE4 as well as amyloid precursor protein (Liu et al. 2000), effectively prolonging their circulation in the neuron’s microenvironment. Collectively, these results suggest that ApoE4 may have a detrimental effect on neurons that synergizes with HIV infection.

This study has limits posed by certain constraints inherent in the cell culture model. The neuronal population differentiated from the hNP1 cell line may be a mixture of cells with varying levels of differentiation. Therefore, there may be additional influences of neuronal maturation on differential gene expression in response to ApoE4 and HIV exposure. The lack of astrocytes does limit detection of the full range of potential effects that ApoE isoforms might exert on neurons by eliminating any astrocyte-mediated interactions. But in this study, the neuronal setting provides the advantage of manipulating only the ApoE phenotype in neurons, and determining differentially expressed genes specific to neurons, without other confounding factors. Microarray and functional genomic studies also have inherent limitations in the algorithms and databases used to probe gene expression, and to define the metabolic pathways and cellular processes enriched by the differentially affected genes.

Our current study with the human hNP1 neural progenitor cell line, along with previous studies using human NEP-derived mixed neuronal and astrocyte cultures (Martinez et al. 2012; Geffin et al. 2013), add to the evidence for the suppressive effect of ApoE4 on neural gene expression, an effect that is magnified in cells exposed to HIV. The response to ApoE4 can be neuronal-specific; astrocytes do not need to be present to see suppressive changes in neuronal gene expression. In the context of HIV infection, ApoE4 can differentially suppress gene expression in cellular processes that impact neurogenesis and neuronal survival.

## Electronic Supplementary Material

ESM 1 (PDF 607 kb)
