# Supplementary material for: Apolipoprotein E4 Suppresses Neuronal-Specific Gene Expression in Maturing Neuronal Progenitor Cells Exposed to HIV
Source: J Neuroimmune Pharmacol. 2017 Mar 20;12(3):462–83. doi: 10.1007/s11481-017-9734-9 (PMC5527073; doi:10.1007/s11481-017-9734-9)
Supplement: Supplementary file 1 — (PDF 607 kb) [file 11481_2017_9734_MOESM1_ESM.pdf]

Supplementary Material

Title: Apolipoprotein E4 suppresses neuronal-specific gene expression in maturing neuronal progenitor cells exposed to HIV

Journal of Neuroimmune Pharmacology

Rebeca Geffin, Ricardo Martinez, Alicia de las Pozas, Biju Issac, and Micheline McCarthy

Corresponding Author:

M McCarthy

Research Service, Bruce W. Carter Veterans Affairs Medical Center, 1201 NW 16th Street, Miami, FL 33125, USA, and Department of Neurology, Miller School of Medicine, University of Miami, 1120 NW 14th St, Miami FL 33136, USA.

Corresponding author:

Micheline McCarthy, MD, PhD.

Associate Professor of Neurology

Department of Neurology, Miller School of Medicine, University of Miami, and

Research Service (151), Bruce W. Carter Veterans Affairs Medical Center

1201 NW 16th Street

Miami, FL 33125

Phone: (305) 575-3151

Fax: (305) 575-3259

e-mail: mmccarth@med.miami.edu

Supplementary Fig. 1 Study Design and Experimental Flow

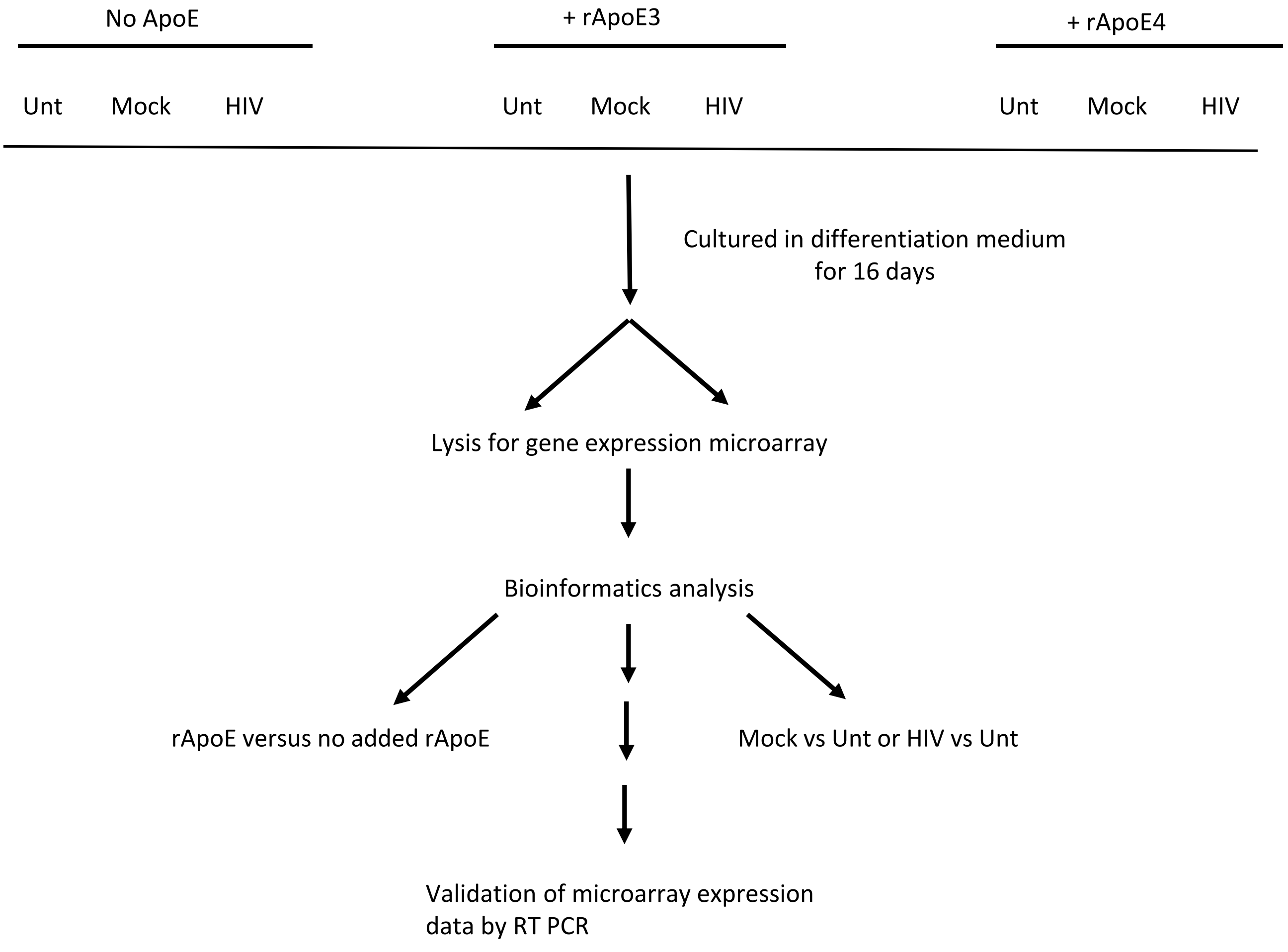

Supplementary Table 1 Genes differentially regulated by rApoE3

Genes differentially regulated in common between [HIV +rAPOE3 vs HIV], [Mock+ rApoE3 vs Mock] and [Unt +rApoE3 vs Unt] n=1

Target ID	FC values	Function
SPRYD5 AKA TRIM51	-1.7<br>-1.9<br>-1.51	Belongs to the TRIM family of proteins. TRIM51 has been associated with precision autophagy

Genes differentially regulated in common between [Mock+ rApoE3 vs Mock] and [Unt + rApoE3 vs Unt] n=2

Target ID	FC values	Function
TRIM48	-1.8<br>-1.61	Belongs to the TRIM family of proteins. The function of this specific protein is unknown, but other TRIM proteins are immune modulators, some have a role in autophagy or have pro neurogenic functions
YWHAE	1.6<br>1.61	This gene product belongs to the 14-3-3 family of proteins which mediate signal transduction by binding to phosphoserine-containing proteins. This highly conserved protein family is found in both plants and mammals, and this protein is 100% identical to the mouse ortholog. It interacts with CDC25 phosphatases, RAF1 and IRS1 proteins, suggesting its role in diverse biochemical activities related to signal transduction, such as cell division and regulation of insulin sensitivity. It has also been implicated in the pathogenesis of small cell lung cancer. Two transcript variants, one protein-coding and the other non-protein-coding, have been found for this gene. [provided by RefSeq, Aug 2008]. YWHAE (Tyrosine 3-Monooxygenase/Tryptophan 5-Monooxygenase Activation Protein, Epsilon) is a Protein Coding gene. Diseases associated with YWHAE include distal 17p13.3 microdeletion syndrome and 17p13.3 microduplication syndrome. Among its related pathways are PI3K-Akt signaling pathway and Signaling by GPCR. GO annotations related to this gene include poly(A) RNA binding and enzyme binding. An important paralog of this gene is YWHAZ. Adapter protein implicated in the regulation of a large spectrum of both general and specialized signaling pathways. Binds to a large number of partners, usually by recognition of a phosphoserine or phosphothreonine motif. Binding generally results in the modulation of the activity of the binding partner<br>14.3.3 proteins are a group of highly conserved proteins that are involved in many vital cellular processes such as metabolism, protein trafficking, signal transduction, apoptosis and cell cycle regulation. 14.3.3 proteins are phospho-serine/-threonine binding proteins.

Genes differentially regulated in common between [HIV +rApoE3 vs HIV] and [Mock+ rApoE3 vs Mock] n=1

Target ID	FC values	Function
STMN2	-1.54<br>-1.68	This gene encodes a member of the stathmin family of phosphoproteins. Stathmin proteins function in microtubule dynamics and signal transduction. The encoded protein plays a regulatory role in neuronal growth and is also thought to be involved in osteogenesis. Reductions in the expression of this gene have been associated with Down's syndrome and Alzheimer's disease. Alternatively spliced transcript variants have been observed for this gene. A pseudogene of this gene is located on the long arm of chromosome 6. [provided by RefSeq, Nov 2010]. STMN2 (Stathmin 2) is a Protein Coding gene. Diseases associated with STMN2 include creutzfeldt-jakob disease and alzheimer disease. GO annotations related to this gene include calcium-dependent protein binding and tubulin binding. An important paralog of this gene is STMN1. Regulator of microtubule stability. When phosphorylated by MAPK8, stabilizes microtubules and consequently controls neurite length in cortical neurons. In the developing brain, negatively regulates the rate of exit from multipolar stage and retards radial migration from the ventricular zone (By similarity).

Genes differentially regulated in common between [HIV +rApoE3 vs HIV] and [Unt +rApo3 vs Unt]   n=2

Target ID	FC values	Function
LPL	-1.61<br>-1.61	LPL (Lipoprotein Lipase) is a Protein Coding gene. Diseases associated with LPL include lipoprotein lipase deficiency and hyperlipidemia, familial combined. Among its related pathways are Signaling by GPCR and Developmental Biology. GO annotations related to this gene include receptor binding and carboxylic ester hydrolase activity. An important paralog of this gene is PNLIPRP1. The primary function of this lipase is the hydrolysis of triglycerides of circulating chylomicrons and very low density lipoproteins (VLDL). Binding to heparin sulfate proteogylcans at the cell surface is vital to the function. The apolipoprotein, APOC2, acts as a coactivator of LPL activity in the presence of lipids on the luminal surface of vascular endothelium (By similarity). Lipases are esterase enzymes that catalyze the hydrolysis of ester bonds within hydrophobic lipids. The diverse class of lipase enzymes includes diacylglycerol lipase (DAGL) and lipoprotein lipase (LPL) and endothelial lipase (LIPG).
SPP1	-1.57<br>-1.61	The protein encoded by this gene is involved in the attachment of osteoclasts to the mineralized bone matrix. The encoded protein is secreted and binds hydroxyapatite with high affinity. The osteoclast vitronectin receptor is found in the cell membrane and may be involved in the binding to this protein. This protein is also a cytokine that upregulates expression of interferon-gamma and interleukin-12. Several transcript variants encoding different isoforms have been found for this gene. [provided by RefSeq, Oct 2011]. SPP1 (Secreted Phosphoprotein 1) is a Protein Coding gene. Diseases associated with SPP1 include tendinitis and malignant pleural mesothelioma. Among its related pathways are PI3K-Akt signaling pathway and Signaling by GPCR. GO annotations related to this gene include cytokine activity and

		extracellular matrix binding. Binds tightly to hydroxyapatite. Appears to form an integral part of the mineralized matrix. Probably important to cell-matrix interaction. Acts as a cytokine involved in enhancing production of interferon-gamma and interleukin-12 and reducing production of interleukin-10 and is essential in the pathway that leads to type I immunity.

Genes differentially regulated only by Mock +rApoE3 vs Mock n=10

Target ID	FC values	Function
CLEC2D	-1.54	This gene encodes a member of the natural killer cell receptor C-type lectin family. The encoded protein inhibits osteoclast formation and contains a transmembrane domain near the N-terminus as well as the C-type lectin-like extracellular domain. Several alternatively spliced transcript variants have been identified for this gene. [provided by RefSeq, Oct 2010]. CLEC2D (C-Type Lectin Domain Family 2, Member D) is a Protein Coding gene. Among its related pathways are Immune System and Immunoregulatory interactions between a Lymphoid and a non-Lymphoid cell. GO annotations related to this gene include transmembrane signaling receptor activity and carbohydrate binding. An important paralog of this gene is CLEC2A. Receptor for KLRB1 that protects target cells against natural killer cell-mediated lysis. Inhibits osteoclast formation. Inhibits bone resorption. Modulates the release of interferon-gamma. Binds high molecular weight sulfated glycosaminoglycans.
KCNIP4	-1.61	This gene encodes a member of the family of voltage-gated potassium (Kv) channel-interacting proteins (KCNIPs), which belong to the recoverin branch of the EF-hand superfamily. Members of the KCNIP family are small calcium binding proteins. They all have EF-hand-like domains, and differ from each other in the N-terminus. They are integral subunit components of native Kv4 channel complexes. They may regulate A-type currents, and hence neuronal excitability, in response to changes in intracellular calcium. This protein member also interacts with presenilin. Multiple alternatively spliced transcript variants encoding distinct isoforms have been identified for this gene. [provided by RefSeq, Jul 2008]. KCNIP4 (Kv Channel Interacting Protein 4) is a Protein Coding gene. Among its related pathways are Regulation of Wnt-mediated beta catenin signaling and target gene transcription. GO annotations related to this gene include calcium ion binding and potassium channel regulator activity. An important paralog of this gene is KCNIP3. Regulatory subunit of Kv4/D (Shal)-type voltage-gated rapidly inactivating A-type potassium channels. Modulates KCND2 channel density, inactivation kinetics and rate of recovery from inactivation in a calcium-dependent and isoform-specific manner (PubMed:11847232, PubMed:18957440, PubMed:23576435). Modulates KCND3/Kv4.3 currents (PubMed:23576435). Isoform 4 does not increase KCND2 expression at the cell membrane

		(PubMed:18957440). Isoform 4 retains KCND3 in the endoplasmic reticulum and negatively regulates its expression at the cell membrane.<br>Voltage-gated potassium channels (KV) belong to the 6-TM family of potassium channel that also comprises the Ca2+-activated Slo (actually 7-TM) and the Ca2+-activated SK subfamilies. The alpha-subunits contain a single pore-forming region and combine to form tetramers.
LOC100130123 AKA NUCKS1	1.54	This gene encodes a nuclear protein that is highly conserved in vertebrates. The conserved regions of the protein contain several consensus phosphorylation sites for casein kinase II and cyclin-dependent kinases, two putative nuclear localization signals, and a basic DNA-binding domain. It is phosphorylated in vivo by Cdk1 during mitosis of the cell cycle. [provided by RefSeq, Aug 2010]<br>NUCKS1 (Nuclear Casein Kinase And Cyclin-Dependent Kinase Substrate 1) is a Protein Coding gene. GO annotations related to this gene include poly(A) RNA binding.
LOC399939 AKA TRIM49D1	-1.8	Belongs to the TRIM family of proteins. The function of TRIM49 has been associated with precision autophagy.
LOC653111 AKA TRIM49C	-1.52	Belongs to the TRIM family of proteins. The function of TRIM49 has been associated with precision autophagy.
LOC729384 AKA TRIM49D2	-1.5	Belongs to the TRIM family of proteins. The function of TRIM49 has been associated with precision autophagy.
PABPC1	1.65	This gene encodes a poly(A) binding protein. The protein shuttles between the nucleus and cytoplasm and binds to the 3' poly(A) tail of eukaryotic messenger RNAs via RNA-recognition motifs. The binding of this protein to poly(A) promotes ribosome recruitment and translation initiation; it is also required for poly(A) shortening which is the first step in mRNA decay. The gene is part of a small gene family including three protein-coding genes and several pseudogenes.[provided by RefSeq, Aug 2010]. PABPC1 (Poly(A) Binding Protein, Cytoplasmic 1) is a Protein Coding gene. Among its related pathways are TGF-Beta Pathway and Apoptotic Pathways in Synovial Fibroblasts. GO annotations related to this gene include nucleic acid binding and RNA binding. An important paralog of this gene is PABPC5. Binds the poly(A) tail of mRNA, including that of its own transcript. May be involved in cytoplasmic regulatory processes of mRNA metabolism such as pre-mRNA splicing. Its function in translational initiation regulation can either be enhanced by PAIP1 or repressed by PAIP2. Can probably bind to cytoplasmic RNA sequences other than poly(A) in vivo. Involved in translationally coupled mRNA turnover. Implicated with other RNA-binding proteins in the cytoplasmic deadenylation/translational and decay interplay of the FOS mRNA mediated by the major coding-region determinant of instability (mCRD) domain. Involved in regulation of nonsense-mediated decay (NMD) of mRNAs containing premature stop codons; for the recognition of premature termination codons (PTC) and initiation of NMD a competitive interaction between UPF1 and PABPC1 with the ribosome-bound release

		factors is proposed. By binding to long poly(A) tails, may protect them from uridylation by ZCCHC6/ZCCHC11 and hence contribute to mRNA stability (PubMed:25480299)
RPL9	-1.53	Ribosomes, the organelles that catalyze protein synthesis, consist of a small 40S subunit and a large 60S subunit. Together these subunits are composed of 4 RNA species and approximately 80 structurally distinct proteins. This gene encodes a ribosomal protein that is a component of the 60S subunit. The protein belongs to the L6P family of ribosomal proteins. It is located in the cytoplasm. As is typical for genes encoding ribosomal proteins, there are multiple processed pseudogenes of this gene dispersed through the genome. Two alternatively spliced transcript variants encoding the same protein have been found for this gene. [provided by RefSeq, Jul 2008]. RPL9 (Ribosomal Protein L9) is a Protein Coding gene. Among its related pathways are Infectious disease and Transport to the Golgi and subsequent modification. GO annotations related to this gene include RNA binding and rRNA binding.
SOCS2	-1.5	This gene encodes a member of the suppressor of cytokine signaling (SOCS) family. SOCS family members are cytokine-inducible negative regulators of cytokine receptor signaling via the Janus kinase/signal transducer and activation of transcription pathway (the JAK/STAT pathway). SOCS family proteins interact with major molecules of signaling complexes to block further signal transduction, in part, by proteasomal depletion of receptors or signal-transducing proteins via ubiquitination. The expression of this gene can be induced by a subset of cytokines, including erythropoietin, GM-CSF, IL10, interferon (IFN)-gamma and by cytokine receptors such as growth horomone receptor. The protein encoded by this gene interacts with the cytoplasmic domain of insulin-like growth factor-1 receptor (IGF1R) and is thought to be involved in the regulation of IGF1R mediated cell signaling. This gene has pseudogenes on chromosomes 20 and 22. Alternative splicing results in multiple transcript variants. [provided by RefSeq, Jul 2012]. SOCS2 (Suppressor Of Cytokine Signaling 2) is a Protein Coding gene. Diseases associated with SOCS2 include vertebral artery occlusion. Among its related pathways are Immune System and TGF-Beta Pathway. GO annotations related to this gene include SH3/SH2 adaptor activity and insulin-like growth factor receptor binding. An important paralog of this gene is SOCS6. SOCS family proteins form part of a classical negative feedback system that regulates cytokine signal transduction. SOCS2 appears to be a negative regulator in the growth hormone/IGF1 signaling pathway. Probable substrate recognition component of a SCF-like ECS (Elongin BC-CUL2/5-SOCS-box protein) E3 ubiquitin-protein ligase complex which mediates the ubiquitination and subsequent proteasomal degradation of target proteins
SYT4	-1.55	SYT4 (Synaptotagmin IV) is a Protein Coding gene. GO annotations related to this gene include calcium ion binding and syntaxin binding. An important paralog of this gene is SYT5.<br>May be involved in Ca(2+)-dependent exocytosis of secretory vesicles through Ca(2+) and phospholipid binding to the C2 domain or may serve as Ca(2+) sensors in the process of vesicular trafficking and exocytosis (By similarity). Plays a role in dendrite formation by melanocytes (PubMed:23999003).

Genes differentially regulated only in Unt +rApoE3 vs Unt n=20

Target ID	FC values	Function
ADM	-1.9	The protein encoded by this gene is a preprohormone which is cleaved to form two biologically active peptides, adrenomedullin and proadrenomedullin N-terminal 20 peptide. Adrenomedullin is a 52 aa peptide with several functions, including vasodilation, regulation of hormone secretion, promotion of angiogenesis, and antimicrobial activity. The antimicrobial activity is antibacterial, as the peptide has been shown to kill E. coli and S. aureus at low concentration. [provided by RefSeq, Aug 2014]. ADM (Adrenomedullin) is a Protein Coding gene. Diseases associated with ADM include malignant hypertension and adrenomyeloneuropathy. Among its related pathways are Signaling by GPCR and HIF-1-alpha transcription factor network. GO annotations related to this gene include receptor binding and adrenomedullin receptor binding. AM and PAMP are potent hypotensive and vasodilatator agents. Numerous actions have been reported most related to the physiologic control of fluid and electrolyte homeostasis. In the kidney, am is diuretic and natriuretic, and both am and pamp inhibit aldosterone secretion by direct adrenal actions. In pituitary gland, both peptides at physiologically relevant doses inhibit basal ACTH secretion. Both peptides appear to act in brain and pituitary gland to facilitate the loss of plasma volume, actions which complement their hypotensive effects in blood vessels
ALCAM	-1.56	This gene encodes activated leukocyte cell adhesion molecule (ALCAM), also known as CD166 (cluster of differentiation 166), which is a member of a subfamily of immunoglobulin receptors with five immunoglobulin-like domains (VVC2C2C2) in the extracellular domain. This protein binds to T-cell differentiation antigene CD6, and is implicated in the processes of cell adhesion and migration. Multiple alternatively spliced transcript variants encoding different isoforms have been found. [provided by RefSeq, Aug 2011]. ALCAM (Activated Leukocyte Cell Adhesion Molecule) is a Protein Coding gene. Diseases associated with ALCAM include appendix adenocarcinoma. Among its related pathways are Developmental Biology and L1CAM interactions. GO annotations related to this gene include receptor binding. An important paralog of this gene is MCAM. Cell adhesion molecule that binds to CD6. Involved in neurite extension by neurons via heterophilic and homophilic interactions. May play a role in the binding of T- and B-cells to activated leukocytes, as well as in interactions between cells of the nervous system
BHLHB2	-1.62	This gene encodes a basic helix-loop-helix protein expressed in various tissues. The encoded protein can interact with ARNTL or compete for E-box binding sites in the promoter of PER1 and repress CLOCK/ARNTL's transactivation of PER1. This gene is believed to be involved in the control of circadian rhythm and cell differentiation. [provided by RefSeq, Feb 2014]

		BHLHE40 (Basic Helix-Loop-Helix Family, Member E40) is a Protein Coding gene. Among its related pathways are Heart Development and Cell Cycle, Mitotic. GO annotations related to this gene include transcription factor activity, sequence-specific DNA binding and protein heterodimerization activity. An important paralog of this gene is BHLHE41. Transcriptional repressor involved in the regulation of the circadian rhythm by negatively regulating the activity of the clock genes and clock-controlled genes. Acts as the negative limb of a novel autoregulatory feedback loop (DEC loop) which differs from the one formed by the PER and CRY transcriptional repressors (PER/CRY loop). Both these loops are interlocked as it represses the expression of PER1/2 and in turn is repressed by PER1/2 and CRY1/2. Represses the activity of the circadian transcriptional activator: CLOCK-ARNTL/BMAL1 ARNTL2/BMAL2 heterodimer by competing for the binding to E-box elements (5-CACGTG-3) found within the promoters of its target genes. Negatively regulates its own expression and the expression of DBP and BHLHE41/DEC2. Acts as a corepressor of RXR and the RXR-LXR heterodimers and represses the ligand-induced RXRA and NR1H3/LXRA transactivation activity. May be involved in the regulation of chondrocyte differentiation via the cAMP pathway.
CALD1	-1.64	This gene encodes a calmodulin- and actin-binding protein that plays an essential role in the regulation of smooth muscle and nonmuscle contraction. The conserved domain of this protein possesses the binding activities to Ca(2+)-calmodulin, actin, tropomyosin, myosin, and phospholipids. This protein is a potent inhibitor of the actin-tropomyosin activated myosin MgATPase, and serves as a mediating factor for Ca(2+)-dependent inhibition of smooth muscle contraction. Alternative splicing of this gene results in multiple transcript variants encoding distinct isoforms. [provided by RefSeq, Jul 2008]. CALD1 (Caldesmon 1) is a Protein Coding gene. Diseases associated with CALD1 include kidney leiomyosarcoma and conventional leiomyosarcoma. Among its related pathways are Integrin Pathway and Metabolism. GO annotations related to this gene include actin binding and myosin binding. Actin- and myosin-binding protein implicated in the regulation of actomyosin interactions in smooth muscle and nonmuscle cells (could act as a bridge between myosin and actin filaments). Stimulates actin binding of tropomyosin which increases the stabilization of actin filament structure. In muscle tissues, inhibits the actomyosin ATPase by binding to F-actin. This inhibition is attenuated by calcium-calmodulin and is potentiated by tropomyosin. Interacts with actin, myosin, two molecules of tropomyosin and with calmodulin. Also play an essential role during cellular mitosis and receptor capping. Involved in Schwann cell migration during peripheral nerve regeneration (By similarity).
CEBPD	-1.64	The protein encoded by this intronless gene is a bZIP transcription factor which can bind as a homodimer to certain DNA regulatory regions. It can also form heterodimers with the related protein CEBP-alpha. The encoded protein is important in the regulation of genes involved in immune and inflammatory responses, and may be involved in the regulation of genes associated with activation and/or differentiation of

		macrophages. The cytogenetic location of this locus has been reported as both 8p11 and 8q11. [provided by RefSeq, Sep 2010]. CEBPD (CCAAT/Enhancer Binding Protein (C/EBP), Delta) is a Protein Coding gene. Among its related pathways are Developmental Biology and IL6-mediated signaling events. GO annotations related to this gene include transcription factor activity, sequence-specific DNA binding and RNA polymerase II core promoter proximal region sequence-specific DNA binding. An important paralog of this gene is CEBPE. UniProtKB/Swiss-Prot for CEBPD Gene. C/EBP is a DNA-binding protein that recognizes two different motifs: the CCAAT homology common to many promoters and the enhanced core homology common to many enhancers. Important transcriptional activator in the regulation of genes involved in immune and inflammatory responses, may play an important role in the regulation of the several genes associated with activation and/or differentiation of macrophages
COTL1	-1.55	This gene encodes one of the numerous actin-binding proteins which regulate the actin cytoskeleton. This protein binds F-actin, and also interacts with 5-lipoxygenase, which is the first committed enzyme in leukotriene biosynthesis. Although this gene has been reported to map to chromosome 17 in the Smith-Magenis syndrome region, the best alignments for this gene are to chromosome 16. The Smith-Magenis syndrome region is the site of two related pseudogenes. [provided by RefSeq, Jul 2008]. COTL1 (Coactosin-Like F-Actin Binding Protein 1) is a Protein Coding gene. GO annotations related to this gene include enzyme binding and actin binding. Binds to F-actin in a calcium-independent manner. Has no direct effect on actin depolymerization. Acts as a chaperone for ALOX5 (5LO), influencing both its stability and activity in leukotrienes synthesis.
CYP26A1	1.58	This gene encodes a member of the cytochrome P450 superfamily of enzymes. The cytochrome P450 proteins are monooxygenases which catalyze many reactions involved in drug metabolism and synthesis of cholesterol, steroids and other lipids. This endoplasmic reticulum protein acts on retinoids, including all-trans-retinoic acid (RA), with both 4-hydroxylation and 18-hydroxylation activities. This enzyme regulates the cellular level of retinoic acid which is involved in regulation of gene expression in both embryonic and adult tissues. Two alternatively spliced transcript variants of this gene, which encode the distinct isoforms, have been reported. [provided by RefSeq, Jul 2008]. CYP26A1 (Cytochrome P450, Family 26, Subfamily A, Polypeptide 1) is a Protein Coding gene. Diseases associated with CYP26A1 include keratomalacia and caudal regression syndrome. Among its related pathways are Signaling by GPCR and Metabolism. GO annotations related to this gene include iron ion binding and oxidoreductase activity, acting on paired donors, with incorporation or reduction of molecular oxygen. An important paralog of this gene is CYP26C1. UniProtKB/Swiss-Prot for CYP26A1 Gene. Plays a key role in retinoic acid metabolism. Acts on retinoids, including all-trans-retinoic acid (RA) and its stereoisomer 9-cis-RA. Capable of both 4-hydroxylation and 18-hydroxylation. Responsible for generation of several hydroxylated forms of RA, including 4-OH-RA, 4-oxo-RA and 18-OH-RA. Cytochrome P450 (CYP450) enzymes are a diverse group of catalysts that contains 57

		members in humans. CYPs are usually membrane-bound and are localized to the inner mitochondrial or endoplasmic reticular membrane. CYPs have oxygenase activity.
DUSP6	-1.51	The protein encoded by this gene is a member of the dual specificity protein phosphatase subfamily. These phosphatases inactivate their target kinases by dephosphorylating both the phosphoserine/threonine and phosphotyrosine residues. They negatively regulate members of the mitogen-activated protein (MAP) kinase superfamily (MAPK/ERK, SAPK/JNK, p38), which are associated with cellular proliferation and differentiation. Different members of the family of dual specificity phosphatases show distinct substrate specificities for various MAP kinases, different tissue distribution and subcellular localization, and different modes of inducibility of their expression by extracellular stimuli. This gene product inactivates ERK2, is expressed in a variety of tissues with the highest levels in heart and pancreas, and unlike most other members of this family, is localized in the cytoplasm. Mutations in this gene have been associated with congenital hypogonadotropic hypogonadism. Alternatively spliced transcript variants have been found for this gene. [provided by RefSeq, Jan 2014] DUSP6 (Dual Specificity Phosphatase 6) is a Protein Coding gene. Diseases associated with DUSP6 include hypogonadotropic hypogonadism 19 with or without anosmia and kallmann syndrome. Among its related pathways are MAPK signaling pathway and Immune System. GO annotations related to this gene include phosphatase activity and phosphoprotein phosphatase activity. An important paralog of this gene is SSH1. UniProtKB/Swiss-Prot for DUSP6 Gene. Inactivates MAP kinases. Has a specificity for the ERK family
EGR2	-1.56	The protein encoded by this gene is a transcription factor with three tandem C2H2-type zinc fingers. Defects in this gene are associated with Charcot-Marie-Tooth disease type 1D (CMT1D), Charcot-Marie-Tooth disease type 4E (CMT4E), and with Dejerine-Sottas syndrome (DSS). Multiple transcript variants encoding two different isoforms have been found for this gene. [provided by RefSeq, Oct 2008]. EGR2 (Early Growth Response 2) is a Protein Coding gene. Diseases associated with EGR2 include charcot-marie-tooth disease, type 1d and neuropathy, congenital hypomyelinating. Among its related pathways are Developmental Biology and IL4-mediated signaling events. GO annotations related to this gene include transcription factor activity, sequence-specific DNA binding and ligase activity. An important paralog of this gene is WT1. Sequence-specific DNA-binding transcription factor. Binds to two specific DNA sites located in the promoter region of HOXA4. E3 SUMO-protein ligase helping SUMO1 conjugation to its coregulators NAB1 and NAB2, whose sumoylation down-regulates EGR2 own transcriptional activity.
FSTL5	-1.5	
IER3	-1.7	This gene functions in the protection of cells from Fas- or tumor necrosis factor type alpha-induced apoptosis. Partially degraded and unspliced transcripts are found after virus infection in vitro, but these transcripts are not found in vivo and do not generate a valid protein. [provided by RefSeq, Jul 2008]

		IER3 (Immediate Early Response 3) is a Protein Coding gene. Diseases associated with IER3 include juvenile hyaline fibromatosis and kallmann syndrome. May play a role in the ERK signaling pathway by inhibiting the dephosphorylation of ERK by phosphatase PP2A-PPP2R5C holoenzyme. Acts also as an ERK downstream effector mediating survival. As a member of the NUPR1/RELB/IER3 survival pathway, may provide pancreatic ductal adenocarcinoma with remarkable resistance to cell stress, such as starvation or gemcitabine treatment.
KCTD12	-1.6	KCTD12 (Potassium Channel Tetramerization Domain Containing 12) is a Protein Coding gene. Diseases associated with KCTD12 include gastrointestinal stromal tumor. Among its related pathways are Activation of cAMP-Dependent PKA and Neuropathic Pain-Signaling in Dorsal Horn Neurons. GO annotations related to this gene include poly(A) RNA binding. An important paralog of this gene is KCTD11. Auxiliary subunit of GABA-B receptors that determine the pharmacology and kinetics of the receptor response. Increases agonist potency and markedly alter the G-protein signaling of the receptors by accelerating onset and promoting desensitization (By similarity).
LRIG1	-1.54	LRIG1 (Leucine-Rich Repeats And Immunoglobulin-Like Domains 1) is a Protein Coding gene. Among its related pathways are Signaling by GPCR and Infectious disease. An important paralog of this gene is LRRTM2.<br>Acts as a feedback negative regulator of signaling by receptor tyrosine kinases, through a mechanism that involves enhancement of receptor ubiquitination and accelerated intracellular degradation.
PLS3	-1.52	Plastins are a family of actin-binding proteins that are conserved throughout eukaryote evolution and expressed in most tissues of higher eukaryotes. In humans, two ubiquitous plastin isoforms (L and T) have been identified. Plastin 1 (otherwise known as Fimbrin) is a third distinct plastin isoform which is specifically expressed at high levels in the small intestine. The L isoform is expressed only in hemopoietic cell lineages, while the T isoform has been found in all other normal cells of solid tissues that have replicative potential (fibroblasts, endothelial cells, epithelial cells, melanocytes, etc.). The C-terminal 570 amino acids of the T-plastin and L-plastin proteins are 83% identical. It contains a potential calcium-binding site near the N terminus. Alternate splicing results in multiple transcript variants. [provided by RefSeq, Feb 2010] PLS3 (Plastin 3) is a Protein Coding gene. Diseases associated with PLS3 include bone mineral density qtl18, osteoporosis and x-linked osteoporosis with fractures. GO annotations related to this gene include calcium ion binding and actin binding. An important paralog of this gene is PLS1.<br>Actin-bundling protein found in intestinal microvilli, hair cell stereocilia, and fibroblast filopodia. May play a role in the regulation of bone development
RFTN1	-1.66	May play a pivotal role in the formation and/or maintenance of lipid rafts. May regulate B-cell antigen receptor-mediated signaling.

RNF19A	-1.5	This gene encodes a member of the ring between ring fingers (RBR) protein family, and the encoded protein contains two RING-finger motifs and an in between RING fingers motif. This protein is an E3 ubiquitin ligase that is localized to Lewy bodies, and ubiquitylates synphilin-1, which is an interacting protein of alpha synuclein in neurons. The encoded protein may be involved in amyotrophic lateral sclerosis and Parkinson's disease. Alternative splicing results in multiple transcript variants. [provided by RefSeq, Jul 2013]. RNF19A (Ring Finger Protein 19A, RBR E3 Ubiquitin Protein Ligase) is a Protein Coding gene. Diseases associated with RNF19A include lateral sclerosis and amyotrophic lateral sclerosis 1. Among its related pathways are Parkin-Ubiquitin Proteasomal System pathway. GO annotations related to this gene include ligase activity and transcription factor binding. An important paralog of this gene is ANKIB1. E3 ubiquitin-protein ligase which accepts ubiquitin from E2 ubiquitin-conjugating enzymes UBE2L3 and UBE2L6 in the form of a thioester and then directly transfers the ubiquitin to targeted substrates, such as SNCAIP or CASR. Specifically ubiquitinates pathogenic SOD1 variants, which leads to their proteasomal degradation and to neuronal protection.
SCG2	-1.63	The protein encoded by this gene is a member of the chromogranin/secretogranin family of neuroendocrine secretory proteins. Studies in rodents suggest that the full-length protein, secretogranin II, is involved in the packaging or sorting of peptide hormones and neuropeptides into secretory vesicles. The full-length protein is cleaved to produce the active peptide secretoneurin, which exerts chemotaxic effects on specific cell types, and EM66, whose function is unknown.<br>SCG2 (Secretogranin II) is a Protein Coding gene. Diseases associated with SCG2 include lymph node cancer and collagenous colitis. GO annotations related to this gene include cytokine activity and chemoattractant activity. Secretogranin-2 is a neuroendocrine secretory granule protein, which is the precursor for biologically active peptides
TFPI2	-1.56	This gene encodes a member of the Kunitz-type serine proteinase inhibitor family. The protein can inhibit a variety of serine proteases including factor VIIa/tissue factor, factor Xa, plasmin, trypsin, chymotryspin and plasma kallikrein. This gene has been identified as a tumor suppressor gene in several types of cancer. Alternative splicing results in multiple transcript variants. [provided by RefSeq, Aug 2012]<br><br>TFPI2 (Tissue Factor Pathway Inhibitor 2) is a Protein Coding gene. Diseases associated with TFPI2 include malignant ovarian cyst and rh isoimmunization. Among its related pathways are Cell adhesion. Plasmin signaling and Formation of Fibrin Clot (Clotting Cascade). GO annotations related to this gene include serine-type endopeptidase inhibitor activity and peptidase inhibitor activity. An important paralog of this gene is TFPI. May play a role in the regulation of plasmin-mediated matrix remodeling. Inhibits trypsin, plasmin, factor VIIa/tissue factor and weakly factor Xa. Has no effect on thrombin.

TNFRSF12A	-1.52	TNFRSF12A (Tumor Necrosis Factor Receptor Superfamily, Member 12A) is a Protein Coding gene. Among its related pathways are Immune System and Interleukin receptor SHC signaling. Receptor for TNFSF12/TWEAK. Weak inducer of apoptosis in some cell types. Promotes angiogenesis and the proliferation of endothelial cells. May modulate cellular adhesion to matrix proteins.
TPST2	-1.62	The protein encoded by this gene catalyzes the O-sulfation of tyrosine residues within acidic regions of proteins. The encoded protein is a type II integral membrane protein found in the Golgi body. Two transcript variants encoding the same protein have been found for this gene. [provided by RefSeq, Jul 2008] TPST2 (Tyrosylprotein Sulfotransferase 2) is a Protein Coding gene. GO annotations related to this gene include sulfotransferase activity and protein-tyrosine sulfotransferase activity. An important paralog of this gene is TPST1.<br>Catalyzes the O-sulfation of tyrosine residues within acidic motifs of polypeptides

Genes differentially regulated only in HIV + rApoE3 vs HIV n=0

Detailed functions of genes differentially regulated by rApoE3 from Table 3 in Results. Functions of each gene were copied from information found online at http://www.genecards.org/ which in turn integrates data and information found in various websites. FC values are listed in the following vertical order:
HIV+ rApoE3 vs HIV, Mock + rApoE3 vs Mock, Unt+ rApoE3 vs Unt

Supplementary Table 2 Genes differentially regulated by rApoE4 and their functions

Genes differentially regulated by [HIV+rApoE4 vs HIV], [Mock+rApoE4 vs Mock] and [Unt +rApoE4 vs Unt]

Target ID	FC	FUNCTION
ADM	-1.78<br>-1.55<br>-2.1	The protein encoded by this gene is a preprohormone which is cleaved to form two biologically active peptides, adrenomedullin and proadrenomedullin N-terminal 20 peptide. Adrenomedullin is a 52 aa peptide with several functions, including vasodilation, regulation of hormone secretion, promotion of angiogenesis, and antimicrobial activity. The antimicrobial activity is antibacterial, as the peptide has been shown to kill E. coli and S. aureus at low concentration.
ALCAM	-1.74<br>-1.56<br>-1.94	Cell adhesion molecule that binds to CD6. Involved in neurite extension by neurons via heterophilic and homophilic interactions. May play a role in the binding of T- and B-cells to activated leukocytes, as well as in interactions between cells of the nervous system<br><br>PLoS One. 2013 Jul 26;8(7):e69270. doi: 10.1371/journal.pone.0069270. Print 2013.<br>Mechanisms of HIV entry into the CNS: increased sensitivity of HIV infected CD14+CD16+ monocytes to CCL2 and key roles of CCR2, JAM-A, and ALCAM in diapedesis.<br>Williams DW1, Calderon TM, Lopez L, Carvallo-Torres L, Gaskill PJ, Eugenin EA, Morgello S, Berman JW.<br>As HIV infected individuals live longer, the prevalence of HIV associated neurocognitive disorders is increasing, despite successful antiretroviral therapy. CD14(+)CD16(+) monocytes are critical to the neuropathogenesis of HIV as they promote viral seeding of the brain and establish neuroinflammation. The mechanisms by which HIV infected and uninfected monocytes cross the blood brain barrier and enter the central nervous system are not fully understood. We determined that HIV infection of CD14(+)CD16(+) monocytes resulted in their highly increased transmigration across the blood brain barrier in response to CCL2 as compared to uninfected cells, which did not occur in the absence of the chemokine. This exuberant transmigration of HIV infected monocytes was due, at least in part, to increased CCR2 and significantly heightened sensitivity to CCL2. The entry of HIV infected and uninfected CD14(+)CD16(+) monocytes into the brain was facilitated by significantly increased surface JAM-A, ALCAM, CD99, and PECAM-1, as compared to CD14(+) cells that are CD16 negative. Upon HIV infection, there was an additional increase in surface JAM-A and ALCAM on CD14(+)CD16(+) monocytes isolated from some individuals. Antibodies to ALCAM and JAM-A inhibited the transmigration of both HIV infected and uninfected CD14(+)CD16(+) monocytes across the BBB, demonstrating their importance in facilitating monocyte transmigration and entry into the brain parenchyma. Targeting CCR2, JAM-A, and ALCAM present on CD14(+)CD16(+) monocytes that preferentially infiltrate the

		CNS represents a therapeutic strategy to reduce viral seeding of the brain as well as the ongoing neuroinflammation that occurs during HIV pathogenesis.
C8ORF13	1.71<br>2.03<br>1.89	Associated with systemic sclerosis. Whole blood mRNA gene expression profiling followed by pathway analysis suggested that C8orf13-BLK region was associated with the dysregulation of BCR and NF-κB signaling pathways.
CALD1	-1.71<br>-1.53<br>-1.57	Actin- and myosin-binding protein implicated in the regulation of actomyosin interactions in smooth muscle and nonmuscle cells (could act as a bridge between myosin and actin filaments). Stimulates actin binding of tropomyosin which increases the stabilization of actin filament structure. In muscle tissues, inhibits the actomyosin ATPase by binding to F-actin. This inhibition is attenuated by calcium-calmodulin and is potentiated by tropomyosin. Interacts with actin, myosin, two molecules of tropomyosin and with calmodulin. Also play an essential role during cellular mitosis and receptor capping. Involved in Schwann cell migration during peripheral nerve regeneration
CRABP1	1.6<br>1.605<br>1.89	This gene encodes a specific binding protein for a vitamin A family member and is thought to play an important role in retinoic acid-mediated differentiation and proliferation processes. It is structurally similar to the cellular retinol-binding proteins, but binds only retinoic acid at specific sites within the nucleus, which may contribute to vitamin A-directed differentiation in epithelial tissue.
EFNB3	1.51<br>1.55<br>1.53	EFNB3, a member of the ephrin gene family, is important in brain development as well as in its maintenance. Moreover, since levels of EFNB3 expression were particularly high in several forebrain subregions compared to other brain subregions, it may play a pivotal role in forebrain function. The EPH and EPH-related receptors comprise the largest subfamily of receptor protein-tyrosine kinases and have been implicated in mediating developmental events, particularly in the nervous system. EPH Receptors typically have a single kinase domain and an extracellular region containing a Cys-rich domain and 2 fibronectin type III repeats. The ephrin ligands and receptors have been named by the Eph Nomenclature Committee (1997). Based on their structures and sequence relationships, ephrins are divided into the ephrin-A (EFNA) class, which are anchored to the membrane by a glycosylphosphatidylinositol linkage, and the ephrin-B (EFNB) class, which are transmembrane proteins. The Eph family of receptors are similarly divided into 2 groups based on the similarity of their extracellular domain sequences and their affinities for binding ephrin-A and ephrin-B ligands.
IER3	-1.62<br>-1.52<br>-1.7	This gene functions in the protection of cells from Fas- or tumor necrosis factor type alpha-induced apoptosis. Partially degraded and unspliced transcripts are found after virus infection in vitro, but these transcripts are not found in vivo and do not generate a valid protein. May play a role in the ERK signaling pathway by inhibiting the dephosphorylation of ERK by phosphatase PP2A-PPP2R5C holoenzyme. Acts also as an ERK

		downstream effector mediating survival. As a member of the NUPR1/RELB/IER3 survival pathway, may provide pancreatic ductal adenocarcinoma with remarkable resistance to cell stress, such as starvation or gemcitabine treatment.
LPL	-1.8<br>-1.6<br>-2.03	LPL encodes lipoprotein lipase, which is expressed in heart, muscle, and adipose tissue. LPL functions as a homodimer, and has the dual functions of triglyceride hydrolase and ligand/bridging factor for receptor-mediated lipoprotein uptake. Severe mutations that cause LPL deficiency result in type I hyperlipoproteinemia, while less extreme mutations in LPL are linked to many disorders of lipoprotein metabolism. LPL (Lipoprotein Lipase) is a Protein Coding gene. Diseases associated with LPL include lipoprotein lipase deficiency and familial lipoprotein lipase deficiency. Among its related pathways are Signaling by GPCR and Disease. GO annotations related to this gene include receptor binding and triglyceride lipase activity. An important paralog of this gene is PNLIPRP1.<br>UniProtKB/Swiss-Prot for LPL Gene. The primary function of this lipase is the hydrolysis of triglycerides of circulating chylomicrons and very low density lipoproteins (VLDL). Binding to heparin sulfate proteogylcans at the cell surface is vital to the function. The apolipoprotein, APOC2, acts as a coactivator of LPL activity in the presence of lipids on the luminal surface of vascular endothelium (By similarity).
LRIG1	-1.58<br>-1.53<br>-1.58	Acts as a feedback negative regulator of signaling by receptor tyrosine kinases, through a mechanism that involves enhancement of receptor ubiquitination and accelerated intracellular degradation.
MAB21L2	-1.73<br>-1.73<br>-1.53	This gene is similar to the C. elegans MAB-21 cell fate-determining gene, a downstream target of transforming growth factor-beta signaling. It is thought that this gene may be involved in neural development. The protein encoded by this gene is primarily nuclear, although some cytoplasmic localization has been observed.
MMRN1	1.8<br>1.88<br>1.75	Multimerin is a massive, soluble protein found in platelets and in the endothelium of blood vessels. It is comprised of subunits linked by interchain disulfide bonds to form large, variably sized homomultimers. Multimerin is a factor V/Va-binding protein and may function as a carrier protein for platelet factor V. It may also have functions as an extracellular matrix or adhesive protein. Recently, patients with an unusual autosomal-dominant bleeding disorder (factor V Quebec) were found to have a deficiency of platelet multimerin.
RFTN1	-1.81<br>-1.56<br>-1.86	May play a pivotal role in the formation and/or maintenance of lipid rafts. May regulate B-cell antigen receptor-mediated signaling
SCG2	-1.68<br>-1.51<br>-1.77	The protein encoded by this gene is a member of the chromogranin/secretogranin family of neuroendocrine secretory proteins. Studies in rodents suggest that the full-length protein, secretogranin II, is involved in the packaging or sorting of peptide hormones and neuropeptides into secretory vesicles. The full-length protein is

		cleaved to produce the active peptide secretoneurin, which exerts chemotaxic effects on specific cell types, and EM66, whose function is unknown.
STMN2	-2.75<br>-2.8<br>-2.16	This gene encodes a member of the stathmin family of phosphoproteins. Stathmin proteins function in microtubule dynamics and signal transduction. The encoded protein plays a regulatory role in neuronal growth and is also thought to be involved in osteogenesis. Reductions in the expression of this gene have been associated with Down's syndrome and Alzheimer's disease. Alternatively spliced transcript variants have been observed for this gene. A pseudogene of this gene is located on the long arm of chromosome 6.
SYT4	-2.17<br>-2.3<br>-1.9	May be involved in Ca(2+)-dependent exocytosis of secretory vesicles through Ca(2+) and phospholipid binding to the C2 domain or may serve as Ca(2+) sensors in the process of vesicular trafficking and exocytosis
TRIM48	-2.9<br>-3.1<br>-1.82	Belongs to the TRIM family of proteins. TRIM proteins are immune modulators, some have a role in autophagy or have pro neurogenic functions

Genes differentially regulated by [HIV+ rApoE4 VS HIV] and [Mock +rApoE4 vs Mock]

Target ID	FC	FUNCTION
BHLHE22	-1.535<br>-1.61	BHLHE22 belongs to the basic helix-loop-helix (bHLH) family of transcription factors that regulate cell fate determination, proliferation, and Inhibits DNA binding of TCF3/E47 homodimers and TCF3 (E47)/NEUROD1 heterodimers and acts as a strong repressor of Neurod1 and Myod-responsive genes, probably by heterodimerization with class a basic helix-loop-helix factors. Despite the presence of an intact basic domain, does not bind to DNA differentiation. These proteins function as dimers and bind to an E-box DNA sequence (CANNTG). BHLHE22 is expressed exclusively in the central nervous system and retina (Xu et al., 2002 [PubMed 12213201]).[supplied by OMIM, Jul 2010]
COL4A1	-1.642<br>-1.52	This gene encodes a type IV collagen alpha protein. Type IV collagen proteins are integral components of basement membranes. This gene shares a bidirectional promoter with a paralogous gene on the opposite strand. The protein consists of an amino-terminal 7S domain, a triple-helix forming collagenous domain, and a carboxy-terminal non-collagenous domain. It functions as part of a heterotrimer and interacts with other extracellular matrix components such as perlecans, proteoglycans, and laminins. In addition, proteolytic cleavage of the non-collagenous carboxy-terminal domain results in a biologically active fragment known as arresten, which has anti-angiogenic and tumor suppressor properties. Mutations in this gene cause porencephaly, cerebrovascular disease, and renal and muscular defects. Alternative splicing results in

		multiple transcript variants.
D4S234E	-1.57<br>-1.64	Int J Oncol. 2010 Nov;37(5):1133-41.<br>Identification of NEEP21, encoding neuron-enriched endosomal protein of 21 kDa, as a transcriptional target of tumor suppressor p53.<br>Ohnishi S1, Futamura M, Kamino H, Nakamura Y, Kitamura N, Miyamoto Y, Miyamoto T, Shinogi D, Goda O, Arakawa H.<br>Abstract<br><br>NEEP21, also designated D4S234E or NSG1, is an endosomal protein expressed in neuronal cells under normal conditions. Here, we report that NEEP21 is a direct transcriptional target gene of the tumor suppressor p53. NEEP21 expression is inducible in non-neuronal human cancer cell lines by exposure to adriamycin, hydrogen peroxide, UV and γ-ray in a p53-dependent manner. Chromatin immunoprecipitation assay indicated that a potential p53-binding site (p53BS) is located in intron 1 of the NEEP21 gene. A reporter assay confirmed that p53BS has p53-responsive activity. The heterologous luciferase gene containing p53BS is also transactivated by p73-β and p63-γ. The introduction of the NEEP21 gene into various cancer cell lines suppressed cell growth. Infection with an adenovirus vector containing NEEP21 induced apoptotic cell death via caspase-3 activation in many cancer cell lines. The expression of NEEP21 mRNA was remarkably induced by γ-ray irradiation in the spleen of p53+/+ mice but not in that of p53-/- mice. These results suggest that NEEP21 may play a critical role in apoptosis as a mediator of p53.
DCX	-1.76<br>-1.83	This gene encodes a member of the doublecortin family. The protein encoded by this gene is a cytoplasmic protein and contains two doublecortin domains, which bind microtubules. In the developing cortex, cortical neurons must migrate over long distances to reach the site of their final differentiation. The encoded protein appears to direct neuronal migration by regulating the organization and stability of microtubules. In addition, the encoded protein interacts with LIS1, the regulatory gamma subunit of platelet activating factor acetylhydrolase, and this interaction is important to proper microtubule function in the developing cortex. Mutations in this gene cause abnormal migration of neurons during development and disrupt the layering of the cortex, leading to epilepsy, mental retardation, subcortical band heterotopia ("double cortex" syndrome) in females and lissencephaly ("smooth brain" syndrome) in males. Multiple transcript variants encoding different isoforms have been found for this gene.
EBF3	-1.56<br>-1.57	This gene encodes a member of the early B-cell factor (EBF) family of DNA binding transcription factors. EBF proteins are involved in B-cell differentiation, bone development and neurogenesis, and may also function as tumor suppressors. The encoded protein inhibits cell survival through the regulation of genes involved in cell cycle arrest and apoptosis, and aberrant methylation or deletion of this gene may play a role in multiple malignancies including glioblastoma multiforme and gastric carcinoma

HES5	-1.97<br>-2.22	This gene encodes a member of a family of basic helix-loop-helix transcriptional repressors. The protein product of this gene, which is activated downstream of the Notch pathway, regulates cell differentiation in multiple tissues. Disruptions in the normal expression of this gene have been associated with developmental diseases and cancer.<br>HES5 (Hes Family BHLH Transcription Factor 5) is a Protein Coding gene. Among its related pathways are Signaling by GPCR and Disease. GO annotations related to this gene include chromatin binding and protein dimerization activity. An important paralog of this gene is HES4.<br>Transcriptional repressor of genes that require a bHLH protein for their transcription. Plays an important role as neurogenesis negative regulator (By similarity).
IGFBP3	-1.54<br>-1.51	This gene is a member of the insulin-like growth factor binding protein (IGFBP) family and encodes a protein with an IGFBP domain and a thyroglobulin type-I domain. The protein forms a ternary complex with insulin-like growth factor acid-labile subunit (IGFALS) and either insulin-like growth factor (IGF) I or II. In this form, it circulates in the plasma, prolonging the half-life of IGFs and altering their interaction with cell surface receptors. Alternate transcriptional splice variants, encoding different isoforms, have been characterized.
INA	-1.58<br>-1.56	Neurofilaments are type IV intermediate filament heteropolymers composed of light, medium, and heavy chains. Neurofilaments comprise the axoskeleton and they functionally maintain the neuronal caliber. They may also play a role in intracellular transport to axons and dendrites. This gene is a member of the intermediate filament family and is involved in the morphogenesis of neurons. [provided by RefSeq, Jun 2009]<br>INA (Internexin Neuronal Intermediate Filament Protein, Alpha) is a Protein Coding gene. Diseases associated with INA include prostate small cell carcinoma and wernicke encephalopathy. Among its related pathways are Cytoskeleton remodeling Neurofilaments. GO annotations related to this gene include structural constituent of cytoskeleton. An important paralog of this gene is LMNB2.<br>Class-IV neuronal intermediate filament that is able to self-assemble. It is involved in the morphogenesis of neurons. It may form an independent structural network without the involvement of other neurofilaments or it may cooperate with NF-L to form the filamentous backbone to which NF-M and NF-H attach to form the cross-bridges
LOC100008589	-2.4<br>1.6	RNA28S5 RNA, 28S ribosomal 5
LOC100134006 aka TRIM49C	-2.44<br>-2.63	Belongs to the TRIM family of proteins. TRIM proteins are immune modulators, some have a role in autophagy or have pro neurogenic functions
LOC399939 AKA TRIM49D1	-2.53<br>-2.56	Belongs to the TRIM family of proteins. TRIM proteins are immune modulators, some have a role in autophagy or have pro neurogenic functions

LOC440040	-2.12<br>-2.67	glutamate receptor, metabotropic 5 pseudogene
LOC642362	-1.76<br>-1.8	LOC652436 similar to tripartite motif protein 39 [Homo sapiens (human)]<br>Gene ID: 652436, discontinued on 20-Jun-2009<br>This record has been withdrawn by NCBI because the model on which it was based was not predicted in a later annotation.
LOC653111	-1.85<br>-2.04	Belongs to the TRIM family of proteins. TRIM proteins are immune modulators, some have a role in autophagy or have pro neurogenic functions
LOC729384	-1.883<br>-2.14	TRIM49D2 tripartite motif containing 49D2. Belongs to the TRIM family of proteins. TRIM proteins are immune modulators, some have a role in autophagy or have pro neurogenic functions
NEFM	-1.61<br>-1.51	Neurofilaments are type IV intermediate filament heteropolymers composed of light, medium, and heavy chains. Neurofilaments comprise the axoskeleton and functionally maintain neuronal caliber. They may also play a role in intracellular transport to axons and dendrites. This gene encodes the medium neurofilament protein. This protein is commonly used as a biomarker of neuronal damage. Alternative splicing results in multiple transcript variants encoding distinct isoforms.
NEUROG2	-1.93<br>-2.14	This gene encodes a neural-specific basic helix-loop-helix (bHLH) transcription factor that can specify a neuronal fate on ectodermal cells and is expressed in neural progenitor cells within the developing central and peripheral nervous systems. The protein product of this gene also plays a role in the differentiation and survival of midbrain dopaminergic neurons. [provided by RefSeq, Apr 2012]<br>NEUROG2 (Neurogenin 2) is a Protein Coding gene. Diseases associated with NEUROG2 include alcohol dependence. GO annotations related to this gene include sequence-specific DNA binding and E-box binding. An important paralog of this gene is BHLHA15.<br>Transcriptional regulator. Involved in neuronal differentiation. Activates transcription by binding to the E box (5-CANNTG-3)
PPP1R14C	1.796<br>1.73	The degree of protein phosphorylation is regulated by a balance of protein kinase and phosphatase activities. Protein phosphatase-1 (PP1; see MIM 176875) is a signal-transducing phosphatase that influences neuronal activity, protein synthesis, metabolism, muscle contraction, and cell division. PPP1R14C is an inhibitor of PP1
SEMA3A	1.5<br>1.55	This gene is a member of the semaphorin family and encodes a protein with an Ig-like C2-type (immunoglobulin-like) domain, a PSI domain and a Sema domain. This secreted protein can function as either a chemorepulsive agent, inhibiting axonal outgrowth, or as a chemoattractive agent, stimulating the growth of apical dendrites. In both cases, the protein is vital for normal neuronal pattern development. Increased expression of this protein is associated with schizophrenia and is seen in a variety of human tumor cell lines. Also, aberrant release of this protein is associated with the progression of Alzheimer's disease.<br>SEMA3A (Sema Domain, Immunoglobulin Domain (Ig), Short Basic Domain, Secreted, (Semaphorin) 3A) is a

		Protein Coding gene. Diseases associated with SEMA3A include hypogonadotropic hypogonadism 16 with or without anosmia and kallmann syndrome. Among its related pathways are L1CAM interactions and NF-KappaB Family Pathway. GO annotations related to this gene include receptor activity and chemorepellent activity. An important paralog of this gene is SEMA4G.<br>Involved in the development of the olfactory system and in neuronal control of puberty. Induces the collapse and paralysis of neuronal growth cones. Could serve as a ligand that guides specific growth cones by a motility-inhibiting mechanism. Binds to the complex neuropilin-1/plexin-1.
SFRP1	-1.56<br>-1.56	This gene encodes a member of the SFRP family that contains a cysteine-rich domain homologous to the putative Wnt-binding site of Frizzled proteins. Members of this family act as soluble modulators of Wnt signaling; epigenetic silencing of SFRP genes leads to deregulated activation of the Wnt-pathway which is associated with cancer. This gene may also be involved in determining the polarity of photoreceptor cells in the retina. [provided by RefSeq, Sep 2009]<br>Soluble frizzled-related proteins (sFRPS) function as modulators of Wnt signaling through direct interaction with Wnts. They have a role in regulating cell growth and differentiation in specific cell types. SFRP1 decreases intracellular beta-catenin levels (By similarity). Has antiproliferative effects on vascular cells, in vitro and in vivo, and can induce, in vivo, an angiogenic response. In vascular cell cycle, delays the G1 phase and entry into the S phase (By similarity). In kidney development, inhibits tubule formation and bud growth in metanephroi (By similarity). Inhibits WNT1/WNT4-mediated TCF-dependent transcription.
SOCS2	-1.66<br>-1.74	SOCS family proteins form part of a classical negative feedback system that regulates cytokine signal transduction. SOCS2 appears to be a negative regulator in the growth hormone/IGF1 signaling pathway. Probable substrate recognition component of a SCF-like ECS (Elongin BC-CUL2/5-SOCS-box protein) E3 ubiquitin-protein ligase complex which mediates the ubiquitination and subsequent proteasomal degradation of target proteins
SOX3	-1.78<br>-1.59	Transcription factor required during the formation of the hypothalamo-pituitary axis. May function as a switch in neuronal development. Keeps neural cells undifferentiated by counteracting the activity of proneural proteins and suppresses neuronal differentiation. Required also within the pharyngeal epithelia for craniofacial morphogenesis.
SPRYD5 same as TRIM51	-3.1<br>-3.6	Belongs to the TRIM family of proteins. TRIM proteins are immune modulators, some have a role in autophagy or have pro neurogenic functions

Genes differentially regulated commonly by [HIV+rApoE4 VS HIV] AND [Untreated +rApoE4 vs Untreated]

Target ID	FC	FUNCTION
COL1A2	-1.55<br>-1.56	This gene encodes the pro-alpha2 chain of type I collagen whose triple helix comprises two alpha1 chains and one alpha2 chain. Type I is a fibril-forming collagen found in most connective tissues and is abundant in bone, cornea, dermis and tendon. Mutations in this gene are associated with osteogenesis imperfecta types I-IV, Ehlers-Danlos syndrome type VIIB, recessive Ehlers-Danlos syndrome Classical type, idiopathic osteoporosis, and atypical Marfan syndrome. Symptoms associated with mutations in this gene, however, tend to be less severe than mutations in the gene for the alpha1 chain of type I collagen (COL1A1) reflecting the different role of alpha2 chains in matrix integrity.
CPE	-1.56<br>-1.76	Removes residual C-terminal Arg or Lys remaining after initial endoprotease cleavage during prohormone processing.<br>Processes proinsulin
CYP26A1	1.542<br>1.67	This gene encodes a member of the cytochrome P450 superfamily of enzymes. The cytochrome P450 proteins are monooxygenases which catalyze many reactions involved in drug metabolism and synthesis of cholesterol, steroids and other lipids. This endoplasmic reticulum protein acts on retinoids, including all-trans-retinoic acid (RA), with both 4-hydroxylation and 18-hydroxylation activities. This enzyme regulates the cellular level of retinoic acid which is involved in regulation of gene expression in both embryonic and adult tissues. Two alternatively spliced transcript variants of this gene, which encode the distinct isoforms, have been reported.
FSTL5	-1.59<br>-1.75	Int J Clin Exp Pathol. 2015 Mar 1;8(3):3386-94. eCollection 2015.<br>Down-regulated FSTL5 promotes cell proliferation and survival by affecting Wnt/β-catenin signaling in hepatocellular carcinoma.<br>Zhang D1, Ma X1, Sun W1, Cui P1, Lu Z1.<br><br>Follistatin-like 5 (FSTL5), a member of the follistatin family of genes, encodes a secretory glycoprotein. Previous study revealed that it might play a suppressive role in hepatocellular carcinoma (HCC). However, its clinical significances, biological functions and molecular mechanisms in HCC development are poorly understood. To gain insight to the functions of FSTL5 in HCC, We examined FSTL5 expression pattern in 117 HCC tissue samples. The results of immunohistochemical staining analysis showed that FSTL5 is more commonly down-regulated in HCC compared to adjacent tissues and further clinicopathological analysis showed that its expression level is closely correlated with tumor size, TNM stage, local infiltration and patient prognosis. Both gain function assays and recombinant human FSTL5 protein treatment assays in vitro revealed that over-expressing FSTL5 could inhibit the abilities of cancer cell proliferation and survival. Further, we found that those effects on HCC growth and survival are associated with Wnt/β-catenin signaling. Taken together, all of our results validate that FSTL5 plays a suppressive role in HCC and suggest that down-regulated FSTL5 could elevate abilities of growth and survival of HCC cells by

		activation of Wnt/β-catenin signaling.
KCTD12	-1.65<br>-2,07	Auxiliary subunit of GABA-B receptors that determine the pharmacology and kinetics of the receptor response. Increases agonist potency and markedly alter the G-protein signaling of the receptors by accelerating onset and promoting desensitization
PLS3	-1.6<br>-1.64	Plastins are a family of actin-binding proteins that are conserved throughout eukaryote evolution and expressed in most tissues of higher eukaryotes. In humans, two ubiquitous plastin isoforms (L and T) have been identified. Plastin 1 (otherwise known as Fimbrin) is a third distinct plastin isoform which is specifically expressed at high levels in the small intestine. The L isoform is expressed only in hemopoietic cell lineages, while the T isoform has been found in all other normal cells of solid tissues that have replicative potential (fibroblasts, endothelial cells, epithelial cells, melanocytes, etc.). The C-terminal 570 amino acids of the T-plastin and L-plastin proteins are 83% identical. It contains a potential calcium-binding site near the N terminus. Alternate splicing results in multiple transcript variants.[provided by RefSeq, Feb 2010]<br>PLS3 (Plastin 3) is a Protein Coding gene. Diseases associated with PLS3 include bone mineral density qtl18, osteoporosis and x-linked osteoporosis with fractures. GO annotations related to this gene include calcium ion binding and actin binding. An important paralog of this gene is PLS1.<br>Actin-bundling protein found in intestinal microvilli, hair cell stereocilia, and fibroblast filopodia. May play a role in the regulation of bone development
PRSS35	-1.59<br>-1.91	SERINE PROTEASE
SPP1	-1.51<br>-1.63	The protein encoded by this gene is involved in the attachment of osteoclasts to the mineralized bone matrix. The encoded protein is secreted and binds hydroxyapatite with high affinity. The osteoclast vitronectin receptor is found in the cell membrane and may be involved in the binding to this protein. This protein is also a cytokine that upregulates expression of interferon-gamma and interleukin-12. Several transcript variants encoding different isoforms have been found for this gene.
TNFRSF12A	-1.53<br>-1.73	TNFRSF12A (Tumor Necrosis Factor Receptor Superfamily, Member 12A) is a Protein Coding gene. Diseases associated with TNFRSF12A include glioblastoma multiforme and glioblastoma. Among its related pathways are Akt Signaling and Akt Signaling.<br>Receptor for TNFSF12/TWEAK. Weak inducer of apoptosis in some cell types. Promotes angiogenesis and the proliferation of endothelial cells. May modulate cellular adhesion to matrix proteins.

Genes differentially regulated commonly by [Mock+rApoE4 vs Mock] and [Untreated+rApoE4 VS Untreated]

Target ID	FC	FUNCTION
PABPC1	-1.78<br>-1.65	This gene encodes a poly(A) binding protein. The protein shuttles between the nucleus and cytoplasm and binds to the 3' poly(A) tail of eukaryotic messenger RNAs via RNA-recognition motifs. The binding of this protein to poly(A) promotes ribosome recruitment and translation initiation; it is also required for poly(A) shortening which is the first step in mRNA decay. The gene is part of a small gene family including three protein-coding genes and several pseudogenes.<br>PABPC1 (Poly(A) Binding Protein, Cytoplasmic 1) is a Protein Coding gene. Diseases associated with PABPC1 include myotonic dystrophy 2 and rift valley fever. Among its related pathways are TGF-Beta Pathway and Apoptotic Pathways in Synovial Fibroblasts. GO annotations related to this gene include nucleotide binding and poly(A) binding. An important paralog of this gene is PABPC5. Binds the poly(A) tail of mRNA, including that of its own transcript. May be involved in cytoplasmic regulatory processes of mRNA metabolism such as pre-mRNA splicing. Its function in translational initiation regulation can either be enhanced by PAIP1 or repressed by PAIP2. Can probably bind to cytoplasmic RNA sequences other than poly(A) in vivo. Involved in translationally coupled mRNA turnover. Implicated with other RNA-binding proteins in the cytoplasmic deadenylation/translational and decay interplay of the FOS mRNA mediated by the major coding-region determinant of instability (mCRD) domain. Involved in regulation of nonsense-mediated decay (NMD) of mRNAs containing premature stop codons; for the recognition of premature termination codons (PTC) and initiation of NMD a competitive interaction between UPF1 and PABPC1 with the ribosome-bound release factors is proposed.
YWHAE	1.58<br>1.54	This gene product belongs to the 14-3-3 family of proteins which mediate signal transduction by binding to phosphoserine-containing proteins. This highly conserved protein family is found in both plants and mammals, and this protein is 100% identical to the mouse ortholog. It interacts with CDC25 phosphatases, RAF1 and IRS1 proteins, suggesting its role in diverse biochemical activities related to signal transduction, such as cell division and regulation of insulin sensitivity. It has also been implicated in the pathogenesis of small cell lung cancer. Two transcript variants, one protein-coding and the other non-protein-coding, have been found for this gene.<br>YWHAE (Tyrosine 3-Monooxygenase/Tryptophan 5-Monooxygenase Activation Protein, Epsilon) is a Protein Coding gene. Diseases associated with YWHAE include 16q24.3 microdeletion syndrome and 17p13.3 microduplication syndrome. Among its related pathways are PI3K-Akt signaling pathway and Signaling by FGFR. GO annotations related to this gene include protein heterodimerization activity and protein domain specific binding. An important paralog of this gene is YWHAZ.<br>Adapter protein implicated in the regulation of a large spectrum of both general and specialized signaling pathways. Binds to a large number of partners, usually by recognition of a phosphoserine or phosphothreonine motif. Binding generally results in the modulation of the activity of the binding partner

14.3.3 proteins are a group of highly conserved proteins that are involved in many vital cellular processes such as metabolism, protein trafficking, signal transduction, apoptosis and cell cycle regulation. 14.3.3 proteins are phospho-serine/phospho-threonine binding proteins that have a diverse array of partners including transcription factors, biosynthetic enzymes, cytoskeletal proteins, signalling molecules, apoptosis factors and tumour suppressors. The 14.3.3 family consists of 7 isoforms; beta, gamma, epsilon, sigma, zeta, tau and eta. 14.3.3 proteins are ubiquitously expressed and self assemble into homo- and heterodimers, with the exception of 14.3.3sigma, which exclusively forms homodimers and is found in cells of epithelial origin only. Each monomer contains an independent ligand-binding site, thus the 14.3.3 dimer can interact with two target proteins simultaneously. 14.3.3 proteins are highly rigid structures and ligand binding can induce conformational changes that alter the stability and/or catalytic activity of the ligand. Furthermore, 14.3.3 protein binding can physically occlude sequence-specific or structural motifs on the target that prevent molecular interactions and/or modulate the accessibility of a target protein to modifying enzymes such as kinases, phosphatases and proteases. In addition, 14.3.3 proteins can act as a scaffold molecule to anchor target proteins within close proximity of one another. 14.3.3 proteins represent an integration point for proliferative, survival, apoptotic and stress signalling pathways. Members of the 14.3.3 protein family enhance the activity of many proteins with proliferative and/or survival functions, such as Raf kinases, and antagonise the activity of proteins that promote cell death and senescence, such as Bad, Bim and Bax. In contrast, 14.3.3sigma acts as a tumour suppressor and its expression is upregulated coordinately with p53 and BRAC1. This isoform sequesters cdk1-cyclin B complexes in the cytoplasm, and thus delays cell cycle progression. 14.3.3sigma is also a crucial regulator of translation during mitosis. Because many 14.3.3 interactions are phosphorylation dependent, 14.3.3 proteins have been integrated into the core regulatory pathways that are crucial for normal growth and development. 14.3.3 proteins are directly involved in cellular processes such as cytokinesis, cell-contact inhibition, anchorage-independent growth and cell adhesion, and it is these pathways that often become dysregulated in disease states such as cancer.

Genes differentially regulated only by [HIV+rApoE4 vs HIV]

Target ID	FC	FUNCTION
ACTG2	-1.779	Actins are highly conserved proteins that are involved in various types of cell motility and in the maintenance of the cytoskeleton. Three types of actins, alpha, beta and gamma, have been identified in vertebrates. Alpha actins are found in muscle tissues and are a major constituent of the contractile apparatus. The beta and gamma actins co-exist in most cell types as components of the cytoskeleton and as mediators of internal cell motility. This gene encodes actin gamma 2; a smooth muscle actin found in enteric tissues. Alternative splicing results in multiple transcript

		variants encoding distinct isoforms. Based on similarity to peptide cleavage of related actins, the mature protein of this gene is formed by removal of two N-terminal peptides.
AP2B1	-1.53	The protein encoded by this gene is one of two large chain components of the assembly protein complex 2, which serves to link clathrin to receptors in coated vesicles. The encoded protein is found on the cytoplasmic face of coated vesicles in the plasma membrane. Two transcript variants encoding different isoforms have been found for this gene.<br>AP2B1 (Adaptor-Related Protein Complex 2, Beta 1 Subunit) is a Protein Coding gene. Among its related pathways are Signaling by FGFR and Signaling by FGFR. GO annotations related to this gene include protein complex binding and clathrin binding. An important paralog of this gene is AP4B1.<br>Component of the adaptor protein complex 2 (AP-2). Adaptor protein complexes function in protein transport via transport vesicles in different membrane traffic pathways. Adaptor protein complexes are vesicle coat components and appear to be involved in cargo selection and vesicle formation. AP-2 is involved in clathrin-dependent endocytosis in which cargo proteins are incorporated into vesicles surrounded by clathrin (clathrin-coated vesicles, CCVs) which are destined for fusion with the early endosome. The clathrin lattice serves as a mechanical scaffold but is itself unable to bind directly to membrane components. Clathrin-associated adaptor protein (AP) complexes which can bind directly to both the clathrin lattice and to the lipid and protein components of membranes are considered to be the major clathrin adaptors contributing the CCV formation. AP-2 also serves as a cargo receptor to selectively sort the membrane proteins involved in receptor-mediated endocytosis. AP-2 seems to play a role in the recycling of synaptic vesicle membranes from the presynaptic surface. AP-2 recognizes Y-X-X-[FILMV] (Y-X-X-Phi) and [ED]-X-X-X-L-[LI] endocytosis signal motifs within the cytosolic tails of transmembrane cargo molecules. AP-2 may also play a role in maintaining normal post-endocytic trafficking through the ARF6-regulated, non-clathrin pathway. The AP-2 beta subunit acts via its C-terminal appendage domain as a scaffolding platform for endocytic accessory proteins; at least some clathrin-associated sorting proteins (CLASPs) are recognized by their [DE]-X(1,2)-F-X-X-[FL]-X-X-X-R motif. The AP-2 beta subunit binds to clathrin heavy chain, promoting clathrin lattice assembly; clathrin displaces at least some CLASPs from AP2B1 which probably then can be positioned for further coat assembly.
C2ORF80	-1.656	Unknown
DBNDD1	-1.5	Unknown
FOXC1	-1.67	This gene belongs to the forkhead family of transcription factors which is characterized by a distinct DNA-binding forkhead domain. The specific function of this gene has not yet been determined; however, it has been shown to play a role in the regulation of embryonic and ocular development. Mutations in this gene cause various glaucoma phenotypes including primary congenital glaucoma, autosomal dominant iridogoniodysgenesis anomaly, and Axenfeld-Rieger anomaly.<br>FOXC1 (Forkhead Box C1) is a Protein Coding gene. Diseases associated with FOXC1 include iridogoniodysgenesis, type 1 and axenfeld-rieger syndrome, type 3. Among its related pathways are Heart Development and Wnt / Hedgehog / Notch. GO annotations related to this gene include sequence-specific DNA binding transcription factor

		activity and transcription factor binding. An important paralog of this gene is FOXE3.<br>Binding of FREAC-3 and FREAC-4 to their cognate sites results in bending of the DNA at an angle of 80-90 degrees. Regulates FOXO1 through binding to a conserved element, 5-GTAAACAAA-3 in its promoter region, implicating FOXC1 as an important regulator of cell viability and resistance to oxidative stress in the eye.
KCNK12	-1.8	This gene encodes one of the members of the superfamily of potassium channel proteins containing two pore-forming P domains. The product of this gene has not been shown to be a functional channel, however, it may require other non-pore-forming proteins for activity.<br>KCNK12 (Potassium Channel, Two Pore Domain Subfamily K, Member 12) is a Protein Coding gene. Among its related pathways are Activation of cAMP-Dependent PKA and Activation of cAMP-Dependent PKA. GO annotations related to this gene include potassium channel activity and voltage-gated ion channel activity. An important paralog of this gene is KCNK3.<br>Probable potassium channel subunit. No channel activity observed in heterologous systems. May need to associate with another protein to form a functional channel
RN5S9	-1.54	RNA5S9 (RNA, 5S Ribosomal 9) is an RNA Gene, and is affiliated with the rRNA class.
SCD5	1.7	Stearoyl-CoA desaturase (SCD; EC 1.14.99.5) is an integral membrane protein of the endoplasmic reticulum that catalyzes the formation of monounsaturated fatty acids from saturated fatty acids. SCD may be a key regulator of energy metabolism with a role in obesity and dislipidemia. Four SCD isoforms, Scd1 through Scd4, have been identified in mouse. In contrast, only 2 SCD isoforms, SCD1 (MIM 604031) and SCD5, have been identified in human. SCD1 shares about 85% amino acid identity with all 4 mouse SCD isoforms, as well as with rat Scd1 and Scd2. In contrast, SCD5 shares limited homology with the rodent SCDs and appears to be unique to primates (Wang et al., 2005<br>SCD5 (Stearoyl-CoA Desaturase 5) is a Protein Coding gene. Diseases associated with SCD5 include spondylocostal dysostosis and obesity. Among its related pathways are PPAR signaling pathway and Fatty acid metabolism. GO annotations related to this gene include iron ion binding and stearoyl-CoA 9-desaturase activity. An important paralog of this gene is SCD.<br>Fatty acid delta-9-desaturase that introduces a double bond in fatty acyl-coenzyme A at the delta-9 position.
SLC2A1	-1.56	This gene encodes a major glucose transporter in the mammalian blood-brain barrier. The encoded protein is found primarily in the cell membrane and on the cell surface, where it can also function as a receptor for human T-cell leukemia virus (HTLV) I and II. Mutations in this gene have been found in a family with paroxysmal exertion-induced dyskinesia<br>Glucose is an essential source of energy for mammalian cells, and is also used as a substrate in protein and lipid synthesis. Given its hydrophilic nature, glucose must be transported into the cell by dedicated transporters; these are encoded by genes known collectively as the facilitative glucose transporter gene family (GLUT). There are 13 known members of the GLUT family. Glucose transporters maintain a ready supply of glucose for the cell's metabolic

		activity. During growth and division, the energy demands of a cell are increased; it needs glucose to generate ATP and biomass. Cancer cells, which proliferate at a greater rate, thus require more energy than a normal cell. Aerobic glycolysis - often observed in tumor cells, and also known as the Warburg effect - relies on a high rate of glucose uptake, since the generation of ATP by this process is far less efficient than oxidative phosphorylation. Glucose transporters, in particular GLUT1, have therefore become a target of interest in cancer research, as have glycolytic inhibitors.
SLC7A5	-1.72	SLC7A5 (Solute Carrier Family 7 (Amino Acid Transporter Light Chain, L System), Member 5) is a Protein Coding gene. Diseases associated with SLC7A5 include lysinuric protein intolerance. Among its related pathways are Hemostasis and Glucose / Energy Metabolism. GO annotations related to this gene include peptide antigen binding and neutral amino acid transmembrane transporter activity. An important paralog of this gene is SLC7A7.<br>Sodium-independent, high-affinity transport of large neutral amino acids such as phenylalanine, tyrosine, leucine, arginine and tryptophan, when associated with SLC3A2/4F2hc. Involved in cellular amino acid uptake. Acts as an amino acid exchanger. Involved in the transport of L-DOPA across the blood-brain barrier, and that of thyroid hormones triiodothyronine (T3) and thyroxine (T4) across the cell membrane in tissues such as placenta. Plays a role in neuronal cell proliferation (neurogenesis) in brain. Involved in the uptake of methylmercury (MeHg) when administered as the L-cysteine or D,L-homocysteine complexes, and hence plays a role in metal ion homeostasis and toxicity. Involved in the cellular activity of small molecular weight nitrosothiols, via the stereoselective transport of L-nitrosocysteine (L-CNSO) across the transmembrane. May play an important role in high-grade gliomas. Mediates blood-to-retina L-leucine transport across the inner blood-retinal barrier which in turn may play a key role in maintaining large neutral amino acids as well as neurotransmitters in the neural retina. Acts as the major transporter of tyrosine in fibroblasts.
TAF15	-1.56	This gene encodes a member of the TET family of RNA-binding proteins. The encoded protein plays a role in RNA polymerase II gene transcription as a component of a distinct subset of multi-subunit transcription initiation factor TFIID complexes. Translocations involving this gene play a role in acute leukemia and extraskeletal myxoid chondrosarcoma, and mutations in this gene may play a role in amyotrophic lateral sclerosis. Alternatively spliced transcript variants encoding multiple isoforms have been observed for this gene<br>RNA and ssDNA-binding protein that may play specific roles during transcription initiation at distinct promoters. Can enter the preinitiation complex together with the RNA polymerase II (Pol II).
TNFRSF21	-1.5	This gene encodes a member of the tumor necrosis factor receptor superfamily. The encoded protein activates nuclear factor kappa-B and mitogen-activated protein kinase 8 (also called c-Jun N-terminal kinase 1), and induces cell apoptosis. Through its death domain, the encoded receptor interacts with tumor necrosis factor receptor type 1-associated death domain (TRADD) protein, which is known to mediate signal transduction of tumor necrosis factor receptors. Knockout studies in mice suggest that this gene plays a role in T-helper cell activation, and may be involved in inflammation and immune regulation. [provided by RefSeq, Jul 2013]

		Promotes apoptosis, possibly via a pathway that involves the activation of NF-kappa-B. Can also promote apoptosis mediated by BAX and by the release of cytochrome c from the mitochondria into the cytoplasm. Plays a role in neuronal apoptosis, including apoptosis in response to amyloid peptides derived from APP, and is required for both normal cell body death and axonal pruning. Trophic-factor deprivation triggers the cleavage of surface APP by beta-secretase to release sAPP-beta which is further cleaved to release an N-terminal fragment of APP (N-APP). N-APP binds TNFRSF21; this triggers caspase activation and degeneration of both neuronal cell bodies (via caspase-3) and axons (via caspase-6). Negatively regulates oligodendrocyte survival, maturation and myelination. Plays a role in signaling cascades triggered by stimulation of T-cell receptors, in the adaptive immune response and in the regulation of T-cell differentiation and proliferation. Negatively regulates T-cell responses and the release of cytokines such as IL4, IL5, IL10, IL13 and IFNG by Th2 cells. Negatively regulates the production of IgG, IgM and IgM in response to antigens. May inhibit the activation of JNK in response to T-cell stimulation.

Genes differentially regulated only by [Mock+rApoE4 vs Mock]

Target ID	FC	FUNCTION
ACTA2	-1.52	The protein encoded by this gene belongs to the actin family of proteins, which are highly conserved proteins that play a role in cell motility, structure and integrity. Alpha, beta and gamma actin isoforms have been identified, with alpha actins being a major constituent of the contractile apparatus, while beta and gamma actins are involved in the regulation of cell motility. This actin is an alpha actin that is found in skeletal muscle. Defects in this gene cause aortic aneurysm familial thoracic type 6. Multiple alternatively spliced variants, encoding the same protein, have been identified.
BMP5	1.59	This gene encodes a member of the bone morphogenetic protein family which is part of the transforming growth factor-beta superfamily. The superfamily includes large families of growth and differentiation factors. Bone morphogenetic proteins were originally identified by an ability of demineralized bone extract to induce endochondral osteogenesis in vivo in an extraskeletal site. These proteins are synthesized as prepropeptides, cleaved, and then processed into dimeric proteins. This protein may act as an important signaling molecule within the trabecular meshwork and optic nerve head, and may play a potential role in glaucoma pathogenesis. This gene is differentially regulated during the formation of various tumors.
HS.388347	-1.61	Glial cell line-derived neurotrophic factor (GDNF) and neurturin (NTN) are two structurally related, potent neurotrophic factors that play key roles in the control of neuron survival and differentiation. The protein encoded by this gene is a member of the GDNF receptor family. It is a glycosylphosphatidylinositol(GPI)-linked

		cell surface receptor for both GDNF and NTN, and mediates activation of the RET tyrosine kinase receptor. This gene is a candidate gene for Hirschsprung disease. Multiple alternatively spliced transcript variants have been described for this gene.
HS.544637	-1.5	Unknown
LOC441087	-1.73	Unknown
LRRFIP1	-1.57	Transcriptional repressor which preferentially binds to the GC-rich consensus sequence (5-AGCCCCCGGCG-3) and may regulate expression of TNF, EGFR and PDGFA. May control smooth muscle cells proliferation following artery injury through PDGFA repression. May also bind double-stranded RNA. Positively regulates Toll-like receptor (TLR) signaling in response to agonist probably by competing with the negative FLII regulator for MYD88-binding.
MYT1	-1.52	The protein encoded by this gene is a member of a family of neural specific, zinc finger-containing DNA-binding proteins. The protein binds to the promoter regions of proteolipid proteins of the central nervous system and plays a role in the developing nervous system. [provided by RefSeq, Jul 2008] Binds to the promoter regions of proteolipid proteins of the central nervous system. May play a role in the development of neurons and oligodendrogalia in the CNS. May regulate a critical transition point in oligodendrocyte lineage development by modulating oligodendrocyte progenitor proliferation relative to terminal differentiation and up-regulation of myelin gene transcription
TDP1	-1.56	The protein encoded by this gene is involved in repairing stalled topoisomerase I-DNA complexes by catalyzing the hydrolysis of the phosphodiester bond between the tyrosine residue of topoisomerase I and the 3-prime phosphate of DNA. This protein may also remove glycolate from single-stranded DNA containing 3-prime phosphoglycolate, suggesting a role in repair of free-radical mediated DNA double-strand breaks. This gene is a member of the phospholipase D family and contains two PLD phosphodiesterase domains. Mutations in this gene are associated with the disease spinocerebellar ataxia with axonal neuropathy (SCAN1). While several transcript variants may exist for this gene, the full-length natures of only two have been described to date. These two represent the major variants of this gene and encode the same isoform. DNA repair enzyme that can remove a variety of covalent adducts from DNA through hydrolysis of a 3-phosphodiester bond, giving rise to DNA with a free 3 phosphate. Catalyzes the hydrolysis of dead-end complexes between DNA and the topoisomerase I active site tyrosine residue. Hydrolyzes 3-phosphoglycolates on protruding 3 ends on DNA double-strand breaks due to DNA damage by radiation and free radicals. Acts on blunt-ended double-strand DNA breaks and on single-stranded DNA. Has low 3exonuclease activity and can remove a single nucleoside from the 3end of DNA and RNA molecules with 3hydroxyl groups. Has no exonuclease activity towards DNA or RNA with a 3phosphate.
WNT3	1.54	The WNT gene family consists of structurally related genes which encode secreted signaling proteins. These proteins have been implicated in oncogenesis and in several developmental processes, including regulation of

		cell fate and patterning during embryogenesis. This gene is a member of the WNT gene family. It encodes a protein which shows 98% amino acid identity to mouse Wnt3 protein, and 84% to human WNT3A protein, another WNT gene product. The mouse studies show the requirement of Wnt3 in primary axis formation in the mouse. Studies of the gene expression suggest that this gene may play a key role in some cases of human breast, rectal, lung, and gastric cancer through activation of the WNT-beta-catenin-TCF signaling pathway. This gene is clustered with WNT15, another family member, in the chromosome 17q21 region.<br>WNT3 (Wingless-Type MMTV Integration Site Family, Member 3) is a Protein Coding gene. Diseases associated with WNT3 include tetra-amelia syndrome and tetra-amelia multiple malformations x-linked. Among its related pathways are Signaling by GPCR and Proteoglycans in cancer. GO annotations related to this gene include protein domain specific binding and receptor agonist activity. An important paralog of this gene is WNT1.<br>Ligand for members of the frizzled family of seven transmembrane receptors. Wnt-3 and Wnt-3a play distinct roles in cell-cell signaling during morphogenesis of the developing neural tube (By similarity).

Genes differentially regulated only by [Untreated +rApoE4 vs Untreated]

Target ID	FC	FUNCTION
APOE	-1.59	The protein encoded by this gene is a major apoprotein of the chylomicron. It binds to a specific liver and peripheral cell receptor, and is essential for the normal catabolism of triglyceride-rich lipoprotein constituents. This gene maps to chromosome 19 in a cluster with the related apolipoprotein C1 and C2 genes. Mutations in this gene result in familial dysbetalipoproteinemia, or type III hyperlipoproteinemia (HLP III), in which increased plasma cholesterol and triglycerides are the consequence of impaired clearance of chylomicron and VLDL remnants. Alternative splicing results in multiple transcript variants. [provided by RefSeq, Nov 2014]<br>APOE (Apolipoprotein E) is a Protein Coding gene. Diseases associated with APOE include lipoprotein glomerulopathy and sea-blue histiocyte disease. Among its related pathways are Signaling by GPCR and Lipoprotein metabolism. GO annotations related to this gene include protein homodimerization activity and receptor binding.<br>Mediates the binding, internalization, and catabolism of lipoprotein particles. It can serve as a ligand for the LDL (apo B/E) receptor and for the specific apo-E receptor (chylomicron remnant) of hepatic tissues.
CALB2	-1.58	This gene encodes an intracellular calcium-binding protein belonging to the troponin C superfamily. Members of this protein family have six EF-hand domains which bind calcium. This protein plays a role in diverse cellular functions, including message targeting and intracellular calcium buffering. It also functions as a modulator of

		neuronal excitability, and is a diagnostic marker for some human diseases, including Hirschsprung disease and some cancers. Alternative splicing results in multiple transcript variants. [provided by RefSeq, Jun 2010]<br>CALB2 (Calbindin 2) is a Protein Coding gene. Diseases associated with CALB2 include adenomatoid tumor and sertoli cell tumor. GO annotations related to this gene include calcium ion binding. An important paralog of this gene is SCGN.<br>Calretinin is a calcium-binding protein which is abundant in auditory neurons.
DIRAS2	-1.52	DIRAS2 belongs to a distinct branch of the functionally diverse Ras (see HRAS; MIM 190020) superfamily of monomeric GTPases.[supplied by OMIM, Apr 2004]<br>DIRAS2 (DIRAS Family GTP Binding RAS Like 2) is a Protein Coding gene. GO annotations related to this gene include GTP binding. An important paralog of this gene is RAP2B.<br>Displays low GTPase activity and exist predominantly in the GTP-bound form.
DUSP6	-1.60	The protein encoded by this gene is a member of the dual specificity protein phosphatase subfamily. These phosphatases inactivate their target kinases by dephosphorylating both the phosphoserine/threonine and phosphotyrosine residues. They negatively regulate members of the mitogen-activated protein (MAP) kinase superfamily (MAPK/ERK, SAPK/JNK, p38), which are associated with cellular proliferation and differentiation. Different members of the family of dual specificity phosphatases show distinct substrate specificities for various MAP kinases, different tissue distribution and subcellular localization, and different modes of inducibility of their expression by extracellular stimuli. This gene product inactivates ERK2, is expressed in a variety of tissues with the highest levels in heart and pancreas, and unlike most other members of this family, is localized in the cytoplasm. Mutations in this gene have been associated with congenital hypogonadotropic hypogonadism. Alternatively spliced transcript variants have been found for this gene. [provided by RefSeq, Jan 2014].<br>DUSP6 (Dual Specificity Phosphatase 6) is a Protein Coding gene. Diseases associated with DUSP6 include hypogonadotropic hypogonadism 19 with or without anosmia and normosmic congenital hypogonadotropic hypogonadism. Among its related pathways are Signaling by GPCR and Immune System. GO annotations related to this gene include phosphatase activity and phosphoprotein phosphatase activity. An important paralog of this gene is SSH1. Inactivates MAP kinases. Has a specificity for the ERK family.
EGR2	-1.77	The protein encoded by this gene is a transcription factor with three tandem C2H2-type zinc fingers. Defects in this gene are associated with Charcot-Marie-Tooth disease type 1D (CMT1D), Charcot-Marie-Tooth disease type 4E (CMT4E), and with Dejerine-Sottas syndrome (DSS). Multiple transcript variants encoding two different isoforms have been found for this gene. [provided by RefSeq, Oct 2008]<br>EGR2 (Early Growth Response 2) is a Protein Coding gene. Diseases associated with EGR2 include charcot-marie-tooth disease, type 1d and neuropathy, congenital hypomyelinating. Among its related pathways are Developmental Biology and BDNF signaling pathway. GO annotations related to this gene include transcription factor activity, sequence-specific DNA binding and ligase activity. An important paralog of this gene is EGR1.

		Sequence-specific DNA-binding transcription factor. Binds to two specific DNA sites located in the promoter region of HOXA4.<br>E3 SUMO-protein ligase helping SUMO1 conjugation to its coregulators NAB1 and NAB2, whose sumoylation down-regulates EGR2 own transcriptional activity.
EMP1	-1.90	suppresses cell proliferation in various cancers
GADD45G	1.54	This gene is a member of a group of genes whose transcript levels are increased following stressful growth arrest conditions and treatment with DNA-damaging agents. The protein encoded by this gene responds to environmental stresses by mediating activation of the p38/JNK pathway via MTK1/MEKK4 kinase. The GADD45G is highly expressed in placenta. [provided by RefSeq, Jul 2008]<br>GADD45G (Growth Arrest And DNA Damage Inducible Gamma) is a Protein Coding gene. Among its related pathways are p53 Signaling and ATM Pathway. An important paralog of this gene is GADD45B.<br>UniProtKB/Swiss-Prot for GADD45G Gene<br>Involved in the regulation of growth and apoptosis. Mediates activation of stress-responsive MTK1/MEKK4 MAPKKK.
IRX2	1.53	IRX2 is a member of the Iroquois homeobox gene family. Members of this family appear to play multiple roles during pattern formation of vertebrate embryos.[supplied by OMIM, Apr 2004]<br>IRX2 (Iroquois Homeobox 2) is a Protein Coding gene. GO annotations related to this gene include sequence-specific DNA binding. An important paralog of this gene is IRX3.
ITPRIP	-1.57	This gene encodes a membrane-associated protein that binds the inositol 1,4,5-trisphosphate receptor (ITPR). The encoded protein enhances the sensitivity of ITPR to intracellular calcium signaling. Alternative splicing results in multiple transcript variants. [provided by RefSeq, Dec 2012]<br>ITPRIP (Inositol 1,4,5-Trisphosphate Receptor Interacting Protein) is a Protein Coding gene. An important paralog of this gene is ITPRIPL2.<br>Enhances Ca(2+)-mediated inhibition of inositol 1,4,5-triphosphate receptor (ITPR) Ca(2+) release.
LAMC1	-1.53	Laminins, a family of extracellular matrix glycoproteins, are the major noncollagenous constituent of basement membranes. They have been implicated in a wide variety of biological processes including cell adhesion, differentiation, migration, signaling, neurite outgrowth and metastasis. Laminins, composed of 3 non identical chains: laminin alpha, beta and gamma (formerly A, B1, and B2, respectively), have a cruciform structure consisting of 3 short arms, each formed by a different chain, and a long arm composed of all 3 chains. Each laminin chain is a multidomain protein encoded by a distinct gene. Several isoforms of each chain have been described. Different alpha, beta and gamma chain isomers combine to give rise to different heterotrimeric laminin isoforms which are designated by Arabic numerals in the order of their discovery, i.e. alpha1beta1gamma1 heterotrimer is laminin 1. The biological functions of the different chains and trimer molecules are largely unknown, but some of the chains have been shown to differ with respect to their tissue

		distribution, presumably reflecting diverse functions in vivo. This gene encodes the gamma chain isoform laminin, gamma 1. The gamma 1 chain, formerly thought to be a beta chain, contains structural domains similar to beta chains, however, lacks the short alpha region separating domains I and II. The structural organization of this gene also suggested that it had diverged considerably from the beta chain genes. Embryos of transgenic mice in which both alleles of the gamma 1 chain gene were inactivated by homologous recombination, lacked basement membranes, indicating that laminin, gamma 1 chain is necessary for laminin heterotrimer assembly. It has been inferred by analogy with the strikingly similar 3' UTR sequence in mouse laminin gamma 1 cDNA, that multiple polyadenylation sites are utilized in human to generate the 2 different sized mRNAs (5.5 and 7.5 kb) seen on Northern analysis. [provided by RefSeq, Aug 2011]<br><br>LAMC1 (Laminin Subunit Gamma 1) is a Protein Coding gene. Diseases associated with LAMC1 include anti-p200 pemphigoid and junctional epidermolysis bullosa inversa. Among its related pathways are Degradation of the extracellular matrix and Blood-Brain Barrier and Immune Cell Transmigration: Pathways Overview. GO annotations related to this gene include extracellular matrix structural constituent and glycosphingolipid binding. An important paralog of this gene is LAMC2.<br>Binding to cells via a high affinity receptor, laminin is thought to mediate the attachment, migration and organization of cells into tissues during embryonic development by interacting with other extracellular matrix components.
LIPA	-1.62	This gene encodes lipase A, the lysosomal acid lipase (also known as cholesterol ester hydrolase). This enzyme functions in the lysosome to catalyze the hydrolysis of cholesteryl esters and triglycerides. Mutations in this gene can result in Wolman disease and cholesteryl ester storage disease. Alternatively spliced transcript variants have been found for this gene. [provided by RefSeq, Jan 2014]<br>LIPA (Lipase A, Lysosomal Acid Type) is a Protein Coding gene. Diseases associated with LIPA include wolman disease and cholesterol ester storage disease. Among its related pathways are Lysosome and Phase I, non P450. GO annotations related to this gene include lipase activity and sterol esterase activity. An important paralog of this gene is LIPM.<br><br>Crucial for the intracellular hydrolysis of cholesteryl esters and triglycerides that have been internalized via receptor-mediated endocytosis of lipoprotein particles. Important in mediating the effect of LDL (low density lipoprotein) uptake on suppression of hydroxymethylglutaryl-CoA reductase and activation of endogenous cellular cholesteryl ester formation.
MYH9	-1.52	This gene encodes a conventional non-muscle myosin; this protein should not be confused with the unconventional myosin-9a or 9b (MYO9A or MYO9B). The encoded protein is a myosin IIA heavy chain that contains an IQ domain and a myosin head-like domain which is involved in several important functions, including

		cytokinesis, cell motility and maintenance of cell shape. Defects in this gene have been associated with non-syndromic sensorineural deafness autosomal dominant type 17, Epstein syndrome, Alport syndrome with macrothrombocytopenia, Sebastian syndrome, Fechtner syndrome and macrothrombocytopenia with progressive sensorineural deafness. [provided by RefSeq, Dec 2011]<br>MYH9 (Myosin, Heavy Chain 9, Non-Muscle) is a Protein Coding gene. Diseases associated with MYH9 include fechtner syndrome and sebastian syndrome. Among its related pathways are Signaling by GPCR and Signaling by Rho GTPases. GO annotations related to this gene include poly(A) RNA binding and protein homodimerization activity. An important paralog of this gene is MYH4.<br>UniProtKB/Swiss-Prot for MYH9 Gene<br>Cellular myosin that appears to play a role in cytokinesis, cell shape, and specialized functions such as secretion and capping. During cell spreading, plays an important role in cytoskeleton reorganization, focal contacts formation (in the margins but not the central part of spreading cells), and lamellipodial retraction; this function is mechanically antagonized by MYH10.<br>Myosins are a large family of motor proteins that share the common features of ATP hydrolysis (ATPase enzyme activity), actin binding and potential for kinetic energy transduction. Originally isolated from muscle cells, almost all eukaryotic cells are known to contain myosins.
PECI AKA ECI2	-1.50	This gene encodes a member of the hydratase/isomerase superfamily. The protein encoded is a key mitochondrial enzyme involved in beta-oxidation of unsaturated fatty acids. It catalyzes the transformation of 3-cis and 3-trans-enoyl-CoA esters arising during the stepwise degradation of cis-, mono-, and polyunsaturated fatty acids to the 2-trans-enoyl-CoA intermediates. Alternatively spliced transcript variants have been described.<br>ECI2 (Enoyl-CoA Delta Isomerase 2) is a Protein Coding gene. Among its related pathways are Metabolism and Peroxisomal lipid metabolism. GO annotations related to this gene include receptor binding and dodecenoyl-CoA delta-isomerase activity. An important paralog of this gene is CDY1B.<br>UniProtKB/Swiss-Prot for ECI2 Gene<br>Able to isomerize both 3-cis and 3-trans double bonds into the 2-trans form in a range of enoyl-CoA species. Has a preference for 3-trans substrates (By similarity).
PTGDS	-1.54	The protein encoded by this gene is a glutathione-independent prostaglandin D synthase that catalyzes the conversion of prostaglandin H2 (PGH2) to postaglandin D2 (PGD2). PGD2 functions as a neuromodulator as well as a trophic factor in the central nervous system. PGD2 is also involved in smooth muscle contraction/relaxation and is a potent inhibitor of platelet aggregation. This gene is preferentially expressed in brain. Studies with transgenic mice overexpressing this gene suggest that this gene may be also involved in the regulation of non-rapid eye movement sleep. [provided by RefSeq, Jul 2008]<br>PTGDS (Prostaglandin D2 Synthase) is a Protein Coding gene. Diseases associated with PTGDS include pendred syndrome and deafness, autosomal recessive 4, with enlarged vestibular aqueduct. Among its related pathways

		are Metabolism and Arachidonic acid metabolism. GO annotations related to this gene include transporter activity and retinoid binding. An important paralog of this gene is LCN15.<br>Catalyzes the conversion of PGH2 to PGD2, a prostaglandin involved in smooth muscle contraction/relaxation and a potent inhibitor of platelet aggregation. Involved in a variety of CNS functions, such as sedation, NREM sleep and PGE2-induced allodynia, and may have an anti-apoptotic role in oligodendrocytes. Binds small non-substrate lipophilic molecules, including biliverdin, bilirubin, retinal, retinoic acid and thyroid hormone, and may act as a scavenger for harmful hydrophopic molecules and as a secretory retinoid and thyroid hormone transporter. Possibly involved in development and maintenance of the blood-brain, blood-retina, blood-aqueous humor and blood-testis barrier. It is likely to play important roles in both maturation and maintenance of the central nervous system and male reproductive system.
PTN	-1.62	PTN (Pleiotrophin) is a Protein Coding gene. Diseases associated with PTN include bronchus cancer and breast cancer. Among its related pathways are Apoptotic Pathways in Synovial Fibroblasts and ERK Signaling. GO annotations related to this gene include growth factor activity and protein phosphatase inhibitor activity. An important paralog of this gene is MDK.<br>Secreted growth factor that induces neurite outgrowth and which is mitogenic for fibroblasts, epithelial, and endothelial cells. Binds anaplastic lymphoma kinase (ALK) which induces MAPK pathway activation, an important step in the anti-apoptotic signaling of PTN and regulation of cell proliferation.
RNF19A	-1.74	This gene encodes a member of the ring between ring fingers (RBR) protein family, and the encoded protein contains two RING-finger motifs and an in between RING fingers motif. This protein is an E3 ubiquitin ligase that is localized to Lewy bodies, and ubiquitylates synphilin-1, which is an interacting protein of alpha synuclein in neurons. The encoded protein may be involved in amyotrophic lateral sclerosis and Parkinson's disease. Alternative splicing results in multiple transcript variants. [provided by RefSeq, Jul 2013]<br>RNF19A (Ring Finger Protein 19A, RBR E3 Ubiquitin Protein Ligase) is a Protein Coding gene. Diseases associated with RNF19A include lateral sclerosis and amyotrophic lateral sclerosis 1. Among its related pathways are Immune System and Antigen processing- Ubiquitination and Proteasome degradation. GO annotations related to this gene include ligase activity and transcription factor binding. An important paralog of this gene is RNF144A.<br><br>E3 ubiquitin-protein ligase which accepts ubiquitin from E2 ubiquitin-conjugating enzymes UBE2L3 and UBE2L6 in the form of a thioester and then directly transfers the ubiquitin to targeted substrates, such as SNCAIP or CASR. Specifically ubiquitinates pathogenic SOD1 variants, which leads to their proteasomal degradation and to neuronal protection.
SHC1	-1.52	This gene encodes three main isoforms that differ in activities and subcellular location. While all three are adapter proteins in signal transduction pathways, the longest (p66Shc) may be involved in regulating life span and the effects of reactive oxygen species. The other two isoforms, p52Shc and p46Shc, link activated receptor

		tyrosine kinases to the Ras pathway by recruitment of the GRB2/SOS complex. p66Shc is not involved in Ras activation. Unlike the other two isoforms, p46Shc is targeted to the mitochondrial matrix. Several transcript variants encoding different isoforms have been found for this gene. [provided by RefSeq, Feb 2011]<br>SHC1 (SHC (Src Homology 2 Domain Containing) Transforming Protein 1) is a Protein Coding gene. Diseases associated with SHC1 include astrocytoma and insulin-like growth factor i. Among its related pathways are Platelet activation, signaling and aggregation and Signaling by GPCR. GO annotations related to this gene include protein tyrosine kinase activity and phospholipid binding. An important paralog of this gene is SHC4.<br>Signaling adapter that couples activated growth factor receptors to signaling pathways. Participates in a signaling cascade initiated by activated KIT and KITLG/SCF. Isoform p46Shc and isoform p52Shc, once phosphorylated, couple activated receptor tyrosine kinases to Ras via the recruitment of the GRB2/SOS complex and are implicated in the cytoplasmic propagation of mitogenic signals. Isoform p46Shc and isoform p52Shc may thus function as initiators of the Ras signaling cascade in various non-neuronal systems. Isoform p66Shc does not mediate Ras activation, but is involved in signal transduction pathways that regulate the cellular response to oxidative stress and life span. Isoform p66Shc acts as a downstream target of the tumor suppressor p53 and is indispensable for the ability of stress-activated p53 to induce elevation of intracellular oxidants, cytochrome c release and apoptosis. The expression of isoform p66Shc has been correlated with life span (By similarity). Participates in signaling downstream of the angiopoietin receptor TEK/TIE2, and plays a role in the regulation of endothelial cell migration and sprouting angiogenesis.
TFPI2	-1.69	This gene encodes a member of the Kunitz-type serine proteinase inhibitor family. The protein can inhibit a variety of serine proteases including factor VIIa/tissue factor, factor Xa, plasmin, trypsin, chymotryspin and plasma kallikrein. This gene has been identified as a tumor suppressor gene in several types of cancer. Alternative splicing results in multiple transcript variants. [provided by RefSeq, Aug 2012]<br>TFPI2 (Tissue Factor Pathway Inhibitor 2) is a Protein Coding gene. Diseases associated with TFPI2 include choriocarcinoma and fibrosarcoma. Among its related pathways are Matrix Metalloproteinases and Formation of Fibrin Clot (Clotting Cascade). GO annotations related to this gene include serine-type endopeptidase inhibitor activity and peptidase inhibitor activity. An important paralog of this gene is TFPI.<br>May play a role in the regulation of plasmin-mediated matrix remodeling. Inhibits trypsin, plasmin, factor VIIa/tissue factor and weakly factor Xa. Has no effect on thrombin.

Detailed functions of genes differentially regulated by rApoE4 from Table 4 in Results. Functions of each gene were copied from information found online at http://www.genecards.org/ which in turn integrates data and information found in various websites. In addition, information about gene functions was copied from abstracts of published papers as they appear in NCBI Pubmed. FC values are listed in the following vertical order: HIV+ rApoE4 vs HIV, Mock + rApoE4 vs Mock, Unt+ rApoE4 vs Unt

Supplementary Table 3 Numbers of genes differentially affected by rApoE stratified by culture treatment

Culture Treatment	E3	E4	E3 & E4
Unt	25 (5 unique to E3)<br>2 up, 23 down	45 (25 unique to E4)<br>8 up, 37 down	20<br>1 up, 19 down
Mock	14(4 unique to E3)<br>2 up, 12 down	47 (37 unique to E4)<br>9 up, 38 down	10<br>1 up, 9 down
HIV	4 (0 unique to E3)<br>0 up, 4 down	57 (53 unique to E4)<br>7 up, 50 down	4 (0 up, 4 down)

Genes differentially expressed by rApoE3 and rApoE4 as compared to no added rApoE. Genes with absolute FC values of 1.5 or more (nominal $p \leq 0.05$) are stratified by culture treatment

Supplementary Table 4 GO processes and genes affected/enriched by rApoE3

Enrichment by GO Processes		rApoE3 +HIV vs no rApoE +HIV			rApoE3+Mock vs no rApoE +Mock			rApoE3 Unt vs no rApoE Unt		
Processes	Total genes	p-value	Genes In Data	Network Objects from Active Data	p-value	Genes In Data	Network Objects from Active Data	p-value	Genes In Data	Network Objects from Active Data
1. Regulation of neuron differentiation										
	743	1.673 E-02	2	Osteopontin, STMN2	1.131 E-05	5	14-3-3, Synaptotagmin, Synaptotagmin IV, STMN2, SOCS2	7.973 E-02	3	Osteopontin, FN14/TNFRSF12A, 14-3-3
2. Intrinsic apoptotic signaling pathway										
	245	6.732 E-02	1	SGPP1	5.622 E-03	2	14-3-3 epsilon, 14-3-3	1.866 E-05	5	IEX1, C/EBP, 14-3-3 epsilon, SGPP1, 14-3-3
3. Multicellular organismal process										
	8605	1.870 E-01	4	LPL, Osteopontin, Plastin, STMN2	1.757 E-01	6	14-3-3 epsilon, 14-3-3, Synaptotagmin, Synaptotagmin IV, STMN2, SOCS2	2.397 E-05	23	DEC1 (Stra13), LPL, IEX1, CYP26A1, Menin, MKP-3, C/EBP, LIG-1, T-plastin, Adrenomedullin, Caldesmon, Secretogranin II, CD166, 14-3-3 epsilon, C/EBPdelta, TFPI-2,

										Osteopontin, FN14(TNFRSF12A), 14-3-3, TPST2, EGR2 (Krox20), Plastin, Raftlin
4. Regulation of neurogenesis										
	914	2.477 E-02	2	Osteopontin, STMN2	3.086 E-05	5	14-3-3, Synaptotagmin, Synaptotagmin IV, STMN2, SOCS2	1.275 E-01	3	Osteopontin, FN14(TNFRSF12A), 14-3-3
5. Regulation of apoptotic process										
	1981	4.447 E-01	1	Osteopontin	5.920 E-02	3	14-3-3 epsilon, 14-3-3, SOCS2	3.225 E-05	11	IEX1, Menin, MKP-3, C/EBP, Adrenomedullin, Secretogranin II, 14-3-3 epsilon, Osteopontin, FN14(TNFRSF12A), 14-3-3, EGR2 (Krox20)
6. Regulation of programmed cell death										
	1998	4.476 E-01	1	Osteopontin	6.047 E-02	3	14-3-3 epsilon, 14-3-3, SOCS2	3.494 E-05	11	IEX1, Menin, MKP-3, C/EBP, Adrenomedullin, Secretogranin II, 14-3-3 epsilon,

									Osteopontin, FN14(TNFRSF12A), 14-3-3, EGR2 (Krox20)

7. Positive regulation of neuron differentiation

	450	1.207 E-01	1	STMN2	3.789 E-05	4	Synaptotagmin, Synaptotagmin IV, STMN2, SOCS2	4.632 E-01	1	FN14(TNFRSF12A)

8. Apoptotic signaling pathway

	481	1.286 E-01	1	SGPP1	2.046 E-02	2	14-3-3 epsilon, 14-3-3	4.012 E-05	6	IEX1, C/EBP, 14-3-3 epsilon, SGPP1, FN14(TNFRSF12A), 14-3-3

9. Single-multicellular organism process

	8235	1.632 E-01	4	LPL, Osteopontin, Plastin, STMN2	1.470 E-01	6	14-3-3 epsilon, 14-3-3, Synaptotagmin, Synaptotagmin IV, STMN2, SOCS2	5.519 E-05	22	DEC1 (Stra13), LPL, IEX1, CYP26A1, Menin, MKP-3, C/EBP, LIG-1, T-plastin, Adrenomedullin, Caldesmon, Secretogranin II, CD166, 14-3-3 epsilon, C/EBPdelta, TFPI-2, Osteopontin, FN14(TNFRSF12A),

									14-3-3, EGR2 (Krox20), Plastin, Raftlin
10. Regulation of nervous system development									
	1044	3.179 E-02	2	Osteopontin, STMN2	5.851 E-05	5	14-3-3, Synaptotagmin, Synaptotagmin IV, STMN2, SOCS2	1.693 E-01	3	Osteopontin, FN14(TNFRSF12A), 14-3-3

Genes were analyzed for GO processes using the MetaCoreTM on GeneGo software from Thomson Reuters, and using genes differentially expressed when comparing culture treatments in the presence vs the absence of rApoE3. Input data for this analysis includes only genes for which the absolute FC values (added rApoE3 treatment versus no added rApoE) was 1.5 or more with a nominal p value of p≤0.05

Supplementary Table 5 List of GO processes and genes affected/enriched in rApoE4

Processes	Total Genes	rApoE4 +HIV vs no rApoE +HIV p-value	Genes In Data	Network Objects from Active Data	rApoE4 +Mock vs Mock no rApoE p-value	Genes In Data	Network Objects from Active Data	rApoE4 Unt vs Unt no rApoE p-value	Genes In Data	Network Objects from Active Data
1. Nervous system development										
	2993	1.687 E-12	32	CYP26A1, Ephrin-B3 (1.53), SOCS2 (-1.66, S), LIG-1, DR6(TNFRSF21) (-1.5), Adrenomedullin(ADM) (-1.78), STMN2 (-2.67), HES5 (-1.97), Mab21l2, BETA3, FOXC1 (-1.67), Synaptotagmin IV (-2.17), Synaptotagmin, Menin (MEN1), Doublecortin (-1.76), SOX3 (-1.78), Semaphorin 3A (SEMA3A) (1.5), T-plastin (PLS3) (-1.4), FOXC1/2, SLC7A5 (-1.7), Ephrin-B (EFNB), CD166 (ALCAM) (-1.74), Beta-adaptin 2, Actin,	6.132 E-13	29	MYT1 (-1.51), WNT3 (1.53), Synaptotagmin (SYT1), Menin, Ephrin-B3 (1.53), SOX3 (-1.53), Doublecortin (-1.83), SOCS2 (-1.76), Semaphorin 3A (1.54), LIG-1, Adrenomedullin (-1.55), Ephrin-B, STMN2 (-2.65), WNT, HES5 (-2.2), CD166 (-1.56), Mab21l2 (-1.73), 14-3-3 epsilon, BMP5 (1.59), Actin, NEFM (-1.51), Alpha-internexin, COL4A1 (-1.52), Collagen IV, 14-	1.391 E-07	23	Pleiotrophin (OSF1) (1.43), CYP26A1 (1.58), Synaptotagmin (-1.55), Ephrin-B3, Menin, LIG-1, T-plastin (-1.52), Adrenomedullin (-1.9), Ephrin-B, STMN2, APOE(-1.38), CD166(-1.56), Mab21l2(-1.32), 14-3-3 epsilon(1.61), LAMG1, Shc(1.47), MYH9, Osteopontin (-1.61), FN14(TNFRSF12A) (-1.52), MyHC, 14-3-3, Synaptotagmin IV, EGR2 (Krox20) (-1.56)

				Osteopontin(SPP1) (-1.58), NEFM (-1.61), COL4A1 (-1.64), Alpha-internexin (INA1) FN14(TNFRSF12A) (-1.52), Collagen IV, Neurogenin 2 (-1.9), SFRP1(-1.56)			3-3, BETA3, Synaptotagmin IV, Neurogenin 2 (-2.1), SFRP1			
2. Brain development										
	1018	1.855 E-08	16	HES5, BETA3, FOXC1, Synaptotagmin IV, Synaptotagmin, Menin, Doublecortin, SOX3, Semaphorin 3A, FOXC1/2, Actin, NEFM, COL4A1, Alpha-internexin, Collagen IV, Neurogenin 2	6.588 E-12	18	WNT3, Synaptotagmin, Menin, SOX3, Doublecortin, Semaphorin 3A, WNT, HES5, 14-3-3 epsilon, Actin, NEFM, Alpha-internexin, COL4A1, Collagen IV, 14-3-3, BETA3, Synaptotagmin IV, Neurogenin 2	3.144 E-03	8	Pleiotrophin (OSF1), Synaptotagmin, Menin, 14-3-3 epsilon, MyHC, 14-3-3, Synaptotagmin IV, EGR2 (Krox20)
3. Single-multicellular organism process										
	8235	3.838 E-11	49	EBF3, Carboxypeptidase H, CYP26A1, IEX1, Ephrin-B3, SOCS2, LIG-1, DR6(TNFRSF21),	7.146 E-12	43	MYT1, CRABP1, EBF3, LPL, IEX1, WNT3, Synaptotagmin, Menin, Ephrin-B3,	2.422 E-08	40	CRABP1, LPL, Carboxypeptidase H, IEX1, CEL, Pleiotrophin (OSF1), CYP26A1, Synaptotagmin, Ephrin-B3, Menin, MKP-3, EMP1 (Tmp), LIG-1, T-plastin,

	Adrenomedullin, Multimerin, STMN2, Caldesmon, HES5, Secretogranin II, Mab21l2, ACTG2, BETA3, FOXC1, Synaptotagmin IV, IBP3, TAFs, Plastin, Raftlin, CRABP1, LPL, Synaptotagmin, Menin, Doublecortin, SOX3, Semaphorin 3A, T-plastin, FOXC1/2, EBF4, SLC7A5, IBP, Ephrin-B, Actin muscle, CD166, Beta-adaptin 2, Actin, Osteopontin, NEFM, COL1A2, COL4A1, Alpha-internexin, FN14(TNFRSF12A), Collagen IV, Neurogenin 2, SFRP1	SOX3, Doublecortin, SOCS2, Semaphorin 3A, LIG-1, EBF4, Adrenomedullin, Multimerin, Ephrin-B, IBP, STMN2, Caldesmon, ACTA2, WNT, Actin muscle, Secretogranin II, HES5, CD166, Mab21l2, 14-3-3 epsilon, BMP5, Actin, NEFM, Alpha-internexin, COL4A1, Collagen IV, 14-3-3, BETA3, IBP3, Synaptotagmin IV, Neurogenin 2, Plastin, Raftlin, SFRP1	Adrenomedullin, IRX2, Multimerin, Ephrin-B, STMN2, Caldesmon, APOE, Secretogranin II, CD166, Mab21l2, 14-3-3 epsilon, LIPA, GADD45 gamma, LAMG1, Shc, TFPI-2, MYH9, COL1A2, Osteopontin, FN14(TNFRSF12A), MyHC, 14-3-3, Synaptotagmin IV, EGR2 (Krox20), Plastin, Raftlin
4. Head development			

1068	3.622 E-08	16	HES5, BETA3, FOXC1, Synaptotagmin IV, Synaptotagmin, Menin, Doublecortin, SOX3, Semaphorin 3A, FOXC1/2, Actin, NEFM, COL4A1, Alpha-internexin, Collagen IV, Neurogenin 2	1.460 E-11	18	WNT3, Synaptotagmin, Menin, SOX3, Doublecortin, Semaphorin 3A, WNT, HES5, 14-3-3 epsilon, Actin, NEFM, Alpha-internexin, COL4A1, Collagen IV, 14-3-3, BETA3, Synaptotagmin IV, Neurogenin 2	4.207 E-03	8	Pleiotrophin (OSF1), Synaptotagmin, Menin, 14-3-3 epsilon, MyHC, 14-3-3, Synaptotagmin IV, EGR2 (Krox20)

5. Multicellular organismal development

6112	1.845 E-11	43	EBF3, Carboxypeptidase H, CYP26A1, Ephrin-B3, SOCS2, LIG-1, DR6(TNFRSF21), Adrenomedullin, STMN2, HES5, Secretogranin II, Mab21l2, ACTG2, BETA3, FOXC1, Synaptotagmin IV, TAFs, Plastin, CRABP1, Synaptotagmin,	4.698 E-11	37	MYT1, CRABP1, EBF3, WNT3, Synaptotagmin, Menin, Ephrin-B3, SOX3, Doublecortin, SOCS2, Semaphorin 3A, LIG-1, EBF4, Adrenomedullin, Ephrin-B, IBP, STMN2, ACTA2, WNT,	2.165 E-07	33	CRABP1, Carboxypeptidase H, Pleiotrophin (OSF1), CYP26A1, Synaptotagmin, Ephrin-B3, Menin, MKP-3, EMP1 (Tmp), LIG-1, T-plastin, Adrenomedullin, IRX2, Ephrin-B, STMN2, APOE, Secretogranin II, CD166, Mab21l2, 14-3-3 epsilon, LIPA, GADD45 gamma, LAMG1, Shc, MYH9, COL1A2, Osteopontin, FN14(TNFRSF12A), MyHC, 14-3-3, Synaptotagmin IV, EGR2 (Krox20), Plastin

				Menin, Doublecortin, SOX3, Semaphorin 3A, T-plastin, FOXC1/2, EBF4, SLC7A5, IBP, Ephrin-B, Actin muscle, CD166, Beta-adaptin 2, Actin, Osteopontin, NEFM, COL1A2, COL4A1, Alpha-internexin, FN14(TNFRSF12A), Collagen IV, Neurogenin 2, SFRP1			Actin muscle, Secretogranin II, HES5, CD166, Mab21l2, 14-3-3 epsilon, BMP5, Actin, NEFM, Alpha-internexin, COL4A1, Collagen IV, 14-3-3, BETA3, Synaptotagmin IV, Neurogenin 2, Plastin, SFRP1			
6. Generation of neurons										
	2013	2.804 E-09	23	Ephrin-B3, SOCS2, DR6(TNFRSF21), Adrenomedullin, STMN2, HES5, BETA3, Synaptotagmin IV, Synaptotagmin, Doublecortin, SOX3, Semaphorin 3A, T-plastin, Ephrin-B, CD166, Beta-	2.265 E-11	23	WNT3, Synaptotagmin, Ephrin-B3, SOX3, Doublecortin, SOCS2, Semaphorin 3A, Adrenomedullin, Ephrin-B, STMN2, WNT, HES5, CD166, 14-3-3 epsilon, BMP5, Actin,	1.199 E-07	19	Pleiotrophin (OSF1), Synaptotagmin, Ephrin-B3, T-plastin, Adrenomedullin, Ephrin-B, STMN2, APOE, CD166, 14-3-3 epsilon, LAMG1, Shc, MYH9, Osteopontin, FN14(TNFRSF12A), MyHC, 14-3-3, Synaptotagmin IV, EGR2 (Krox20)

			adaptin 2, Actin, Osteopontin, NEFM, COL4A1, FN14(TNFRSF12A), Collagen IV, Neurogenin 2			NEFM, COL4A1, Collagen IV, 14-3-3, BETA3, Synaptotagmin IV, Neurogenin 2			
7. System development									
	5360	3.786 E-11	40	Carboxypeptidase H, CYP26A1, Ephrin-B3, SOCS2, LIG-1, DR6(TNFRSF21), Adrenomedullin, STMN2, HES5, Secretogranin II, Mab21l2, ACTG2, BETA3, FOXC1, Synaptotagmin IV, TAFs, Plastin, Synaptotagmin, Menin, Doublecortin, SOX3, Semaphorin 3A, T-plastin, FOXC1/2, SLC7A5, IBP, Ephrin-B, Actin muscle, CD166, Beta-adaptin 2, Actin, Osteopontin, NEFM, COL1A2,	2.516 E-10	34	MYT1, WNT3, Synaptotagmin, Menin, Ephrin-B3, SOX3, Doublecortin, SOCS2, Semaphorin 3A, LIG-1, Adrenomedullin, Ephrin-B, IBP, STMN2, ACTA2, WNT, Actin muscle, Secretogranin II, HES5, CD166, Mab21l2, 14-3-3 epsilon, BMP5, Actin, NEFM, Alpha-internexin, COL4A1, Collagen IV, 14-3-3, BETA3, Synaptotagmin	6.692 E-07	30	Carboxypeptidase H, Pleiotrophin (OSF1), CYP26A1, Synaptotagmin, Ephrin-B3, Menin, LIG-1, T-plastin, Adrenomedullin, IRX2, Ephrin-B, STMN2, APOE, Secretogranin II, CD166, Mab21l2, 14-3-3 epsilon, LIPA, GADD45 gamma, LAMG1, Shc, MYH9, COL1A2, Osteopontin, FN14(TNFRSF12A), MyHC, 14-3-3, Synaptotagmin IV, EGR2 (Krox20), Plastin

				COL4A1, Alpha-internexin, FN14(TNFRSF12A), Collagen IV, Neurogenin 2, SFRP1			IV, Neurogenin 2, Plastin, SFRP1			
8. Multicellular organismal process										
	8605	2.382 E-10	49	EBF3, Carboxypeptidase H, CYP26A1, IEX1, Ephrin-B3, SOCS2, LIG-1, DR6(TNFRSF21), Adrenomedullin, Multimerin, STMN2, Caldesmon, HES5, Secretogranin II, Mab21l2, ACTG2, BETA3, FOXC1, Synaptotagmin IV, IBP3, TAFs, Plastin, Raftlin, CRABP1, LPL, Synaptotagmin, Menin, Doublecortin, SOX3, Semaphorin 3A, T-plastin, FOXC1/2, EBF4, SLC7A5, IBP, Ephrin-B, Actin	3.913 E-11	43	MYT1, CRABP1, EBF3, LPL, IEX1, WNT3, Synaptotagmin, Menin, Ephrin-B3, SOX3, Doublecortin, SOCS2, Semaphorin 3A, LIG-1, EBF4, Adrenomedullin, Multimerin, Ephrin-B, IBP, STMN2, Caldesmon, ACTA2, WNT, Actin muscle, Secretogranin II, HES5, CD166, Mab21l2, 14-3-3 epsilon, BMP5, Actin, NEFM, Alpha-internexin,	1.013 E-07	40	CRABP1, LPL, Carboxypeptidase H, IEX1, CEL, Pleiotrophin (OSF1), CYP26A1, Synaptotagmin, Ephrin-B3, Menin, MKP-3, EMP1 (Tmp), LIG-1, T-plastin, Adrenomedullin, IRX2, Multimerin, Ephrin-B, STMN2, Caldesmon, APOE, Secretogranin II, CD166, Mab21l2, 14-3-3 epsilon, LIPA, GADD45 gamma, LAMG1, Shc, TFPI-2, MYH9, COL1A2, Osteopontin, FN14(TNFRSF12A), MyHC, 14-3-3, Synaptotagmin IV, EGR2 (Krox20), Plastin, Raftlin

				muscle, CD166, Beta-adaptin 2, Actin, Osteopontin, NEFM, COL1A2, COL4A1, Alpha-internexin, FN14(TNFRSF12A), Collagen IV, Neurogenin 2, SFRP1			COL4A1, Collagen IV, 14-3-3, BETA3, IBP3, Synaptotagmin IV, Neurogenin 2, Plastin, Raftlin, SFRP1			
9. Neurogenesis										
	2118	7.460 E-09	23	Ephrin-B3, SOCS2, DR6(TNFRSF21), Adrenomedullin, STMN2, HES5, BETA3, Synaptotagmin IV, Synaptotagmin, Doublecortin, SOX3, Semaphorin 3A, T-plastin, Ephrin-B, CD166, Beta-adaptin 2, Actin, Osteopontin, NEFM, COL4A1, FN14(TNFRSF12A), Collagen IV, Neurogenin 2	6.378 E-11	23	WNT3, Synaptotagmin, Ephrin-B3, SOX3, Doublecortin, SOCS2, Semaphorin 3A, Adrenomedullin, Ephrin-B, STMN2, WNT, HES5, CD166, 14-3-3 epsilon, BMP5, Actin, NEFM, COL4A1, Collagen IV, 14-3-3, BETA3, Synaptotagmin IV, Neurogenin 2	2.671 E-07	19	Pleiotrophin (OSF1), Synaptotagmin, Ephrin-B3, T-plastin, Adrenomedullin, Ephrin-B, STMN2, APOE, CD166, 14-3-3 epsilon, LAMG1, Shc, MYH9, Osteopontin, FN14(TNFRSF12A), MyHC, 14-3-3, Synaptotagmin IV, EGR2 (Krox20)

10. Developmental process

	6992	7.708 E-11	45	EBF3, Carboxypeptidase H, CYP26A1, IEX1, Ephrin-B3, SOCS2, LIG-1, DR6(TNFRSF21), Adrenomedullin, STMN2, HES5, Secretogranin II, Mab21l2, ACTG2, BETA3, FOXC1, Synaptotagmin IV, IBP3, TAFs, Plastin, CRABP1, Synaptotagmin, Menin, Doublecortin, SOX3, Semaphorin 3A, T-plastin, FOXC1/2, EBF4, SLC7A5, IBP, Ephrin-B, Actin muscle, CD166, Beta-adaptin 2, Actin, Osteopontin, NEFM, COL1A2, COL4A1, Alpha-internexin, FN14(TNFRSF12A), Collagen IV, Neurogenin 2, SFRP1	7.945 E-11	39	MYT1, CRABP1, EBF3, IEX1, WNT3, Synaptotagmin, Menin, Ephrin-B3, SOX3, Doublecortin, SOCS2, Semaphorin 3A, LIG-1, EBF4, Adrenomedullin, Ephrin-B, IBP, STMN2, ACTA2, WNT, Actin muscle, Secretogranin II, HES5, CD166, Mab21l2, 14-3-3 epsilon, BMP5, Actin, NEFM, Alpha-internexin, COL4A1, Collagen IV, 14-3-3, BETA3, IBP3, Synaptotagmin IV, Neurogenin 2, Plastin, SFRP1	1.674 E-06	34	CRABP1, Carboxypeptidase H, IEX1, Pleiotrophin (OSF1), CYP26A1, Synaptotagmin, Ephrin-B3, Menin, MKP-3, EMP1 (Tmp), LIG-1, T-plastin, Adrenomedullin, IRX2, Ephrin-B, STMN2, APOE, Secretogranin II, CD166, Mab21l2, 14-3-3 epsilon, LIPA, GADD45 gamma, LAMG1, Shc, MYH9, COL1A2, Osteopontin, FN14(TNFRSF12A), MyHC, 14-3-3, Synaptotagmin IV, EGR2 (Krox20), Plastin

Genes were analyzed for GO processes using the MetaCoreTM on GeneGo software from Thomson Reuters, and using genes differentially expressed when comparing culture treatments in the presence vs the absence of rApoE4. Input data for this analysis includes only genes for which the absolute FC values (added rApoE4 treatment versus no added rApoE) was 1.5 or more with a nominal p value of $p \leq 0.05$. The FC values of rApoE vs no rApoE for genes enriching the top process (Nervous system development) with absolute values higher than 1.2 are listed

Supplementary Table 6 Comparison of GO processes enriched by rApoE3 and rApoE4, stratified by culture treatment.

Enrichment by GO Processes				Unt +rApoE4/Unt no rApoE			Unt +rApoE3/Unt no rApoE		
#	Processes	Total	Min(p-value)	p-value	In Data	Network Objects from Active Data	p-value	In Data	Network Objects from Active Data
1.	System development								
		5860	1.629E-21	1.629E-21	172	Ephrin-B3(1.53), Fjx1 (-1.29), Alpha-actinin(-1.35), c-Maf(1.29), SOCS2(-1.26), ARID5B(-1.32), Ectodin(-1.28), RhoJ(-1.31), IRX2(1.53), Histone H2, NAV2, COL5A2(-1.44), LIPA (-1.61), NEBL(1.27), Histone H2A, PHLDA1(-1.28), LRRN3(-1.39), MYH9(-1.58), Catalase(1.3), Carbonic anhydrase II(-1.32), Plastin(-1.6), BDNF(-1.4), SFRP4(1.33), LPA1 receptor(-1.31), LRFN5(-1.42), Pleiotrophin (OSF1)(-1.62), MKP-3(-1.6), TXNIP (VDUP1)(1.3), Annexin I(-1.42), T-plastin(-1.6), TSLC1(1.28), Collagen V(-1.44), APOE(-1.59), IBP5(-1.26), ARS2(1.32), GADD45 gamma (1.54), BMP5 (1.36), ARGBP2(-1.24), PHF14(1.29), NEFM (-1.4), ADAM19(-1.26), SPRY1(-1.36), DCOR(1.25), BMP7(1.29), Lpd, CEL, FZD1(1.29), Myosin I-1.23), RBM4(1.28), MIAT(1.29), SULF1(-1.27), Tau (MAPT)(-1.3), DAN(-1.25), Fibrillin 2 (-1.48), COL11A1(-1.48), LIG-1, SMYD2(-1.26), FGF12(-1.28), SLC4A7(-1.25), Adrenomedullin(-2.1), ID2(1.46), TIM, Mab21l2(-1.53), VGF(-1.45), ROBO3(-1.31),	7.641E-11	91	BAF47, Fjx1(-1.25), Tissue kallikreins, Beta-2-microglobulin (-1.3), Ectodin(-1.36), DR6(TNFRSF21)(-1.34), IRX2(1.39), Histone H2(1.23), NAV2(-1.2), COL5A2(-1.45), PAFAH gamma(1.34), LIPA(-1.48), Shc(1.47), PRNP(-1.34), SDFR1(-1.35), Histone H2A, JNK(MAPK8-10)(1.36), LRRN3 (-1.36), C3orf58(-1.44), PLOD3 (-1.27), Carbonic anhydrase II (-1.37), Plastin(-1.52), BDNF (-1.26), HAS, ZFP36L2(-1.27), AIRE(1.23), SFRP4(1.4), Pleiotrophin (OSF1)(1.43), NRF2(-1.28), Menin, MKP-3(-1.51), Annexin I(-1.3), T-plastin(-1.52), Collagen V(-1.46), HDL proteins, APOE(-1.38), MSK2(-1.28), IBP5(-1.32), ARS2, 14-3-3 epsilon(1.61), GADD45 gamma(1.4), BMP5(1.25), BEX1(1.23), LAMB1(1.43), PDZ-GEF1(-1.25), NEFM(-1.3), Nestin(1.25), ARP2(-1.26), SPRY1(-1.27), PKC, BMP7(1.32), EGR2 (Krox20)(-

Irx6(1.35), Wee1(-1.25), CD24(1.37), ID3(1.27), G6PD(-1.3), Synaptotagmin IV(-1.89), hnRNP A1(1.32), DPPA4(1.34), 14-3-3 gamma(1.54), Creatine kinase(1.31), MA2A1, WNT3(1.47), Synaptotagmin, PIRB, MRLC2, MRCL3, Heme oxygenase 1(-1.26), R-cadherin(1.31), Rod1(-1.22), PTPR-zeta(1.26), IBP, ASNS(-1.41), COL1A2(-1.54), FN14(TNFRSF12A)(-1.52), Cytohesin2(1.27), Neurogenin 2(-1.9), PP2A catalytic, FLRT3(-1.37), Carboxypeptidase H(-1.75), MRLC, CD147, EGR1(-1.4), Frizzled, FNDC3B(-1.28), Shc(-1.5), PRNP(-1.46), SDFR1(-1.31), CHKB, C3orf58(-1.43), BRM(-1.31), Shootin1 (KIAA1598)(-1.37), PLOD3(-1.29), PTOP, AIRE(1.31), MYT(-1.38), NRF2(-141), Menin, Semaphorin 3A(1.49), 8ODP1.25) (MYO1B(-1.39), Connexin 43(-1.29), HDL proteins, SIX5(1.32), DNER(-1.32), KCRB(1.31), Osteonectin(-1.3), 14-3-3 epsilon(1.53), CLIC4(-1.25), NFIB(-1.25), hnRNP K(1.25), CTGF(-1.26), LAMB1(-1.34), PDZ-GEF1, Pirin(1.28), ARP2(-1.28), Galpha(i)-specific EDG GPCRs, MyHC, Tropomyosin (-1.3), ITGAV(-1.28), EGR2 (Krox20)(-1.71), Carbohydrate sulfotransferases(-1.36), CYP26A1(1.67), BMP receptor 1.56), Lpd, CEL, CYP26A1(1.58), K-cadherin (CDH6)(-1.38), ICAP-1(-1.26), C/EBP(-1.63), Ankyrin-B(-1.27), Fibrillin 2(-1.47), COL11A1(-1.47), LIG-1, Adrenomedullin(-1.9), ASSY(-1.33), Secretogranin II(-1.62), Mab21l2(-1.32), ZNF148(-1.3), MSK1/2 (RPS6KA5/4)(-1.28), Fibrillin(-1.47), AP1S2(-1.25), ERM, G6PD(-1.26), Nectin-3, LIFR(-1.28), hnRNP A1(1.25), DEC1 (Stra13), Heme oxygenase 1(-1.28), Rod1(-1.25), AP-1 sigma subunits(-1.25), ZC3HDC5, IBP, CD166(-1.56), LAMG1, Caveolin-1(-1.31), C/EBPdelta(-1.63), COL1A2(-1.44), Osteopontin(-1.61), IFITM3(-1.33), FN14(TNFRSF12A)(-1.52), 14-3-3, H2AFY2(1.25)

				2(-1.29), Ankyrin-B (-1.26), MIP26, LLGL1, STMN2(-2.16), Alpha-actinin 1(-1.35), ASSY(-1.41), Secretogranin II(-1.76), Histone H1, Fibrillin, AP1S2(-1.28), MMD, ERM, BETA3, MALT1(-1.3), CLN2 (Tripeptidyl-peptidase I)(-1.26), INSIG1(-1.25), AP-1 sigma subunits(-1.26), Ephrin-B(1.53), WNT, CD166(-1.94), LAMG1(-1.5), NF-I, Osteopontin(-1.65), Alpha-internexin(-1.27), FNBP1(-1.33), SMRT(-1.32), Midkine(1.27), IRS6(DOK5)(-1.27), Tropomyosin-1(-1.26), 14-3-3, IGFBP7/8, H2AFY2(1.34), Galpha(q)-specific EDG GPCRs, M6B (1.8)			
2. Regulation of developmental process							
	3134	2.660E-19	2.660E-19	113	METRNL, LYRIC, Alpha-actinin, c-Maf, SOCS2, Ectodin, RhoJ, Histone H2, ABCA1, TMEM90B, COL5A2, Sts-1, Histone H2A, LRRN3, MYH9, Carbonic anhydrase II, BDNF, SFRP4, LPA1 receptor, Pleiotrophin (OSF1), MKP-3, Annexin I, Collagen V, APOE, IBP5, ARS2, MYADM, BMP5, PHF14, NEFM, SPRY1, BMP7, FZD1, NEK6, DIO3, RBM4, SULF1, Tau (MAPT), DAN, Fibrillin 2, GC1QBP, Adrenomedullin, ID2, FMNL2, COCH, CD24, ID3, G6PD, Synaptotagmin IV, 14-3-3 gamma, MA2A1, WNT3, Synaptotagmin, PIRB, MRLC2, MRCL3, Heme	1.201E-11	63	LYRIC, Tissue kallikreins, Beta-2-microglobulin, Ectodin, DR6(TNFRSF21), TBC1D16, Histone H2, ABCA1, TMEM90B, COL5A2, Sts-1, SDFR1, Histone H2A, JNK(MAPK8-10), LRRN3, Carbonic anhydrase II, CLEC2D, BDNF, HAS, ZFP36L2, Kindlin-2, SFRP4, LPL, Pleiotrophin (OSF1), NRF2, Menin, MKP-3, Annexin I, NUCB2, Collagen V, HDL proteins, APOE, IBP5, ARS2, BMP5, BEX1, PDZ-GEF1, NEFM, SPRY1, PKC, Fibulin-1, BMP7, NEK6, ICAP-1, C/EBP, Fibrillin 2, Osteocrin,

oxygenase 1, DDAH1, R-cadherin, Rod1, PTPR-zeta, IBP, FN14(TNFRSF12A), Cytohesin2, Neurogenin 2, PP2A catalytic, FLRT3, Carboxypeptidase H, MRLC, LL5alpha, EGR1, Frizzled, FNDC3B, SDFR1, Shootin1 (KIAA1598), CLEC2D, Kindlin-2, LPL, NRF2, Menin, Semaphorin 3A, Connexin 43, HDL proteins, Osteonectin, NFIB, hnRNP K, CTGF, PDZ-GEF1, Galpha(i)-specific EDG GPCRs, MyHC, Tropomyosin, ITGAV, BMP receptor 2, Osteocrin, STMN2, Histone H1, Fibrillin, MMD, ERM, IBP3, INSIG1, Ephrin-B, WNT, NF-I, Osteopontin, SMRT, Thrombospondin 2, Tropomyosin-1, 14-3-3, IGFBP7/8, H2AFY2, Galpha(q)-specific EDG GPCRs, M6B

Adrenomedullin, Fibrillin, COCH, ERM, G6PD, IBP3, Heme oxygenase 1, Rod1, IBP, Caveolin-1, C/EBPdelta, Osteopontin, FN14(TNFRSF12A), Thrombospondin 2, 14-3-3, H2AFY2

3. Multicellular organism development

6641	2.679E-19	2.679E-19	181	Ephrin-B3, Fjx1, Alpha-actinin, c-Maf, SOCS2, ARID5B, Ectodin, RhoJ, IRX2, Histone H2, Aurora-B, NAV2, COL5A2, LIPA, NEBL, Histone H2A, PHLDA1, LRRN3, MYH9, Catalase, Carbonic anhydrase II, Plastin, BDNF, SFRP4, LPA1 receptor, LRFN5, Pleiotrophin (OSF1), MKP-3, TXNIP (VDUP1), Annexin I, T-plastin, TSLC1, Collagen V, CIA/ASF1, APOE, IBP5, ARS2, GADD45 gamma, BMP5, ARGBP2, PHF14, LBH, NEFM, ADAM19, SPRY1, FUT8, DCOR, BMP7, Lpd, CEL, FZD1, Myosin I, RBM4, MIAT, SULF1, Tau (MAPT), DAN, Fibrillin 2, COL11A1, LIG-1, SMYD2, TPI1, FGF12, SLC4A7, Adrenomedullin, ID2, TIM, Mab21l2, VGF, ROBO3, Irx6, Wee1, CD24, ID3, G6PD, Synaptotagmin IV, hnRNP A1, DPPA4, 14-3-3 gamma, Creatine kinase, MA2A1, WNT3, Synaptotagmin, PIRB, MRLC2, MRCL3, Heme oxygenase 1, R-cadherin, Rod1, PTPR-zeta, IBP, ASNS, COL1A2, FN14(TNFRSF12A), Cytohesin2, Neurogenin 2, PP2A catalytic, FLRT3, Carboxypeptidase H, MRLC, CD147, EGR1, Frizzled, FNDC3B, Shc, PRNP, SDFR1, CHKB, C3orf58, BRM, Shootin1 (KIAA1598), PLOD3, PTOP, AIRE, MYT1, NRF2, Menin, Semaphorin	2.299E-09	95	BAF47, Fjx1, Tissue kallikreins, Beta-2-microglobulin, Ectodin, DR6(TNFRSF21), IRX2, Histone H2, NAV2, COL5A2, PAFAH gamma, LIPA, Shc, PRNP, SDFR1, Histone H2A, JNK(MAPK8-10), LRRN3, C3orf58, PLOD3, Carbonic anhydrase II, Plastin, BDNF, HAS, ZFP36L2, AIRE, SFRP4, Pleiotrophin (OSF1), NRF2, Menin, MKP-3, Annexin I, T-plastin, Collagen V, HDL proteins, APOE, MSK2, IBP5, ARS2, 14-3-3 epsilon, GADD45 gamma, BMP5, BEX1, LAMB1, PDZ-GEF1, NEFM, Nestin, ARP2, SPRY1, PKC, Fibulin-1, BMP7, EGR2 (Krox20), Lpd, CEL, CYP26A1, K-cadherin (CDH6), ICAP-1, C/EBP, Ankyrin-B, Fibrillin 2, COL11A1, LIG-1, Osteocrin, Adrenomedullin, ASSY, Secretogranin II, Mab21l2, ZNF148, MSK1/2 (RPS6KA5/4), Fibrillin, AP1S2, MID1, ERM, G6PD, Nectin-3, LIFR, hnRNP A1, DEC1 (Stra13), Heme oxygenase 1, Rod1, AP-1 sigma subunits, ZC3HDC5, IBP, HOOK1, CD166, LAMG1, Caveolin-1, C/EBPdelta, COL1A2, Osteopontin, IFITM3,

3A, 8ODP, MYO1B, Connexin 43, HDL proteins, SIX5, DNER, KCRB, Osteonectin, 14-3-3 epsilon, SPRY4, CLIC4, NFIB, hnRNP K, CTGF, LAMB1, PDZ-GEF1, Pirin, ARP2, Galpha(i)-specific EDG GPCRs, MyHC, Tropomyosin, ITGAV, EGR2 (Krox20), Carbohydrate sulfotransferases, CYP26A1, BMP receptor 2, Ankyrin-B, EMP1 (Tmp), MIP26, LLGL1, Osteocrin, STMN2, Alpha-actinin 1, ASSY, Secretogranin II, Histone H1, Fibrillin, AP1S2, MMD, ERM, BETA3, MALT1, CLN2 (Tripeptidyl-peptidase I), CRABP1, INSIG1, AP-1 sigma subunits, Ephrin-B, WNT, CD166, LAMG1, NF-I, Osteopontin, Alpha-internexin, FNBP1, SMRT, Midkine, IRS6(DOK5), Tropomyosin-1, 14-3-3, IGFBP7/8, H2AFY2, Galpha(q)-specific EDG GPCRs, M6B	FN14(TNFRSF12A), 14-3-3, H2AFY2

4. Single-multicellular organism process

7650	8.943E-19	8.943E-19	197	Ephrin-B3, Fjx1, Alpha-actinin, c-Maf, Substance P, SOCS2, ARID5B, Ectodin, RhoJ, IRX2, Histone H2, ABCA1, Aurora-B, NAV2, COL5A2, AK1D1, LIPA, NEBL, Histone H2A, PHLDA1, LRRN3, Optineurin, MYH9, Catalase, Carbonic anhydrase II, Plastin, BDNF, SFRP4, LPA1 receptor, LRFN5, Pleiotrophin (OSF1), MKP-3, TXNIP (VDUP1), Annexin I, T-plastin, TSLC1, Collagen V, CIA/ASF1, APOE, IBP5, ARS2, GADD45 gamma, BMP5, ARGBP2, PHF14, LBH, NEFM, ADAM19, SPRY1, FUT8, DCOR, BMP7, Lpd, CEL, FZD1, Myosin I, RBM4, MIAT, SULF1, Tau (MAPT), DAN, Fibrillin 2, COL11A1, LIG-1, GC1QBP, SMYD2, TPI1, FGF12, SLC4A7, Adrenomedullin, ID2, TIM, Multimerin, Mab21l2, VGF, TFPI-2, ROBO3, Irx6, Wee1, CD24, ID3, G6PD, Synaptotagmin IV, hnRNP A1, DPPA4, 14-3-3 gamma, Creatine kinase, MA2A1, WNT3, Synaptotagmin, PIRB, MRLC2, MRCL3, Heme oxygenase 1, R-cadherin, Rod1, PTPR-zeta, IBP, ASNS, COL1A2, FN14(TNFRSF12A), Cytohesin2, Neurogenin 2, PP2A catalytic, FLRT3, Carboxypeptidase H, MRLC, CD147, LAMP2, EGR1, Frizzled, FNDC3B, Shc, PRNP,	1.414E-09	105	BAF47, Fjx1, Tissue kallikreins, Beta-2-microglobulin, Ectodin, DR6(TNFRSF21), IRX2, Histone H2, ABCA1, NAV2, COL5A2, PKC-delta, PAFAH gamma, LIPA, Shc, PRNP, SDFR1, Histone H2A, JNK(MAPK8-10), LRRN3, Optineurin, C3orf58, PLOD3, Carbonic anhydrase II, Plastin, BDNF, HAS, ZFP36L2, AIRE, SFRP4, LPL, Pleiotrophin (OSF1), NRF2, Menin, MKP-3, Annexin I, T-plastin, Collagen V, HDL proteins, APOE, MSK2, IBP5, ARS2, 14-3-3 epsilon, GADD45 gamma, BMP5, BEX1, LAMB1, PDZ-GEF1, NEFM, SCN7A, Nestin, ARP2, SPRY1, PKC, Fibulin-1, BMP7, EGR2 (Krox20), Lpd, HP1, CEL, CYP26A1, K-cadherin (CDH6), ICAP-1, C/EBP, Ankyrin-B, Fibrillin 2, COL11A1, LIG-1, Osteocrin, Adrenomedullin, Desmuslin, ASSY, Secretogranin II, Mab21l2, ZNF148, MSK1/2 (RPS6KA5/4), TFPI-2, Fibrillin, AP1S2, MID1, ERM, G6PD, IBP3, Nectin-3, LIFR, hnRNP A1, Raftlin, DEC1 (Stra13), Heme oxygenase 1, Rod1, AP-1 sigma subunits, ZC3HDC5, IBP, HOOK1, CD166, LAMG1,

SDFR1, CHKB, C3orf58, BRM, Shootin1 (KIAA1598), PLOD3, PTOP, AIRE, MYT1, LPL, NRF2, Menin, Semaphorin 3A, 8ODP, MYO1B, Connexin 43, HDL proteins, SIX5, DNER, KCRB, Osteonectin, 14-3-3 epsilon, SPRY4, CLIC4, NFIB, hnRNP K, CTGF, LAMB1, PDZ-GEF1, Pirin, SCN7A, ARP2, Galpha(i)-specific EDG GPCRs, MyHC, Tropomyosin, ITGAV, EGR2 (Krox20), Carbohydrate sulfotransferases, CYP26A1, BMP receptor 2, Ankyrin-B, EMP1 (Tmp), MIP26, LLGL1, Osteocrin, STMN2, Alpha-actinin 1, Desmuslin, ASSY, Secretogranin II, Histone H1, Fibrillin, AP1S2, Ca-ATPase2, MMD, ERM, BETA3, MALT1, IBP3, CLN2 (Tripeptidyl-peptidase I), Raftlin, CRABP1, Cox15, INSIG1, LFA-3, AP-1 sigma subunits, Ephrin-B, WNT, CD166, LAMG1, NF-I, Osteopontin, Alpha-internexin, FNBP1, SMRT, Midkine, IRS6(DOK5), Tropomyosin-1, 14-3-3, IGFBP7/8, H2AFY2, Galpha(q)-specific EDG GPCRs, M6B	Caveolin-1, C/EBPdelta, COL1A2, Osteopontin, IFITM3, FN14(TNFRSF12A), 14-3-3, H2AFY2

5. Anatomical Structure Development

7180	2.533E-18	2.533E-18	188	SPAG16, Ephrin-B3, Fjx1, Alpha-actinin, c-Maf, SOCS2, ARID5B, Ectodin, RhoJ, IRX2, Histone H2, Aurora-B, NAV2, COL5A2, LIPA, NEBL, Histone H2A, PHLDA1, LRRN3, MYH9, Catalase, Carbonic anhydrase II, Plastin, BDNF, SMYD3, SFRP4, LPA1 receptor, LRFN5, Pleiotrophin (OSF1), MKP-3, TXNIP (VDUP1), Annexin I, T-plastin, TSLC1, TMEFF2, Collagen V, CIA/ASF1, APOE, IBP5, ARS2, GADD45 gamma, MYADM, BMP5, ARGBP2, PHF14, LBH, NEFM, ADAM19, SPRY1, FUT8, DCOR, BMP7, Lpd, CEL, IEX1, FZD1, Myosin I, RBM4, MIAT, SULF1, Tau (MAPT), DAN, Fibrillin 2, COL11A1, LIG-1, SMYD2, TPI1, FGF12, SLC4A7, Adrenomedullin, ID2, TIM, Mab21l2, VGF, ROBO3, Irx6, Wee1, CD24, ID3, G6PD, Synaptotagmin IV, hnRNP A1, DPPA4, 14-3-3 gamma, Creatine kinase, MA2A1, WNT3, Synaptotagmin, PIRB, MRLC2, MRCL3, Heme oxygenase 1, R-cadherin, Rod1, PTPR-zeta, IBP, ASNS, COL1A2, FN14(TNFRSF12A), Cytohesin2, Neurogenin 2, PP2A catalytic, FLRT3, Carboxypeptidase H, MRLC, CD147, EGR1, Frizzled, FNDC3B, Shc, PRNP, SDFR1, CHKB, C3orf58, BRM, Shootin1	6.016E-09	99	BAF47, Fjx1, Tissue kallikreins, Beta-2-microglobulin, Ectodin, DR6(TNFRSF21), IRX2, Histone H2, NAV2, COL5A2, PAFAH gamma, LIPA, Shc, PRNP, SDFR1, Histone H2A, JNK(MAPK8-10), LRRN3, C3orf58, PLOD3, Carbonic anhydrase II, Plastin, BDNF, HAS, ZFP36L2, AIRE, Kindlin-2, SFRP4, Pleiotrophin (OSF1), NRF2, Menin, MKP-3, Annexin I, T-plastin, TMEFF2, Collagen V, HDL proteins, APOE, MSK2, IBP5, ARS2, 14-3-3 epsilon, GADD45 gamma, BMP5, BEX1, LAMB1, PDZ-GEF1, NEFM, Nestin, UGCG, ARP2, SPRY1, PKC, Fibulin-1, BMP7, EGR2 (Krox20), Lpd, CEL, IEX1, CYP26A1, K-cadherin (CDH6), ICAP-1, C/EBP, Ankyrin-B, Fibrillin 2, COL11A1, LIG-1, Osteocrin, Adrenomedullin, ASSY, Secretogranin II, Mab21l2, ZNF148, MSK1/2 (RPS6KA5/4), Fibrillin, AP1S2, MID1, ERM, G6PD, Nectin-3, LIFR, hnRNP A1, DEC1 (Stra13), Heme oxygenase 1, Rod1, AP-1 sigma subunits, ZC3HDC5, IBP, HOOK1, CD166, LAMG1, Caveolin-1, C/EBPdelta,

(KIAA1598), PLOD3, PTOP, AIRE, Kindlin-2, MYT1, NRF2, Menin, Semaphorin 3A, 8ODP, MYO1B, Connexin 43, HDL proteins, SIX5, DNER, KCRB, Osteonectin, 14-3-3 epsilon, SPRY4, CLIC4, NFIB, hnRNP K, CTGF, LAMB1, PDZ-GEF1, Pirin, ARP2, Galpha(i)-specific EDG GPCRs, MyHC, Tropomyosin, ITGAV, EGR2 (Krox20), Carbohydrate sulfotransferases, CYP26A1, BMP receptor 2, Ankyrin-B, EMP1 (Tmp), MIP26, LLGL1, Osteocrin, STMN2, Alpha-actinin 1, ASSY, Secretogranin II, Histone H1, Fibrillin, AP1S2, Ca-ATPase2, MMD, ERM, BETA3, MALT1, CLN2 (Tripeptidyl-peptidase I), CRABP1, INSIG1, AP-1 sigma subunits, Ephrin-B, WNT, CD166, LAMG1, NF-I, Osteopontin, Alpha-internexin, FNBP1, SMRT, Midkine, IRS6(DOK5), Tropomyosin-1, 14-3-3, IGFBP7/8, H2AFY2, Galpha(q)-specific EDG GPCRs, M6B

COL1A2, Osteopontin, IFITM3, FN14(TNFRSF12A), 14-3-3, H2AFY2

6. Animal organ development

	4305	3.408E-18	3.408E-18	135	Fjx1, Alpha-actinin, c-Maf, SOCS2, ARID5B, Ectodin, RhoJ, IRX2, Histone H2, COL5A2, LIPA, NEBL, Histone H2A, PHLDA1, MYH9, Catalase, Carbonic anhydrase II, Plastin, BDNF, SFRP4, LPA1 receptor, Pleiotrophin (OSF1), TXNIP (VDUP1), Annexin I, T-plastin, TSLC1, Collagen V, IBP5, GADD45 gamma, BMP5, ARGBP2, PHF14, NEFM, ADAM19, SPRY1, DCOR, BMP7, FZD1, Myosin I, RBM4, MIAT, SULF1, Tau (MAPT), Fibrillin 2, COL11A1, LIG-1, SMYD2, FGF12, SLC4A7, Adrenomedullin, ID2, TIM, Mab21l2, VGF, Irx6, CD24, ID3, G6PD, Synaptotagmin IV, hnRNP A1, DPPA4, Creatine kinase, MA2A1, WNT3, Synaptotagmin, PIRB, Heme oxygenase 1, Rod1, PTPR-zeta, IBP, ASNS, COL1A2, Neurogenin 2, PP2A catalytic, FLRT3, Carboxypeptidase H, MRLC, CD147, EGR1, Frizzled, FNDC3B, Shc, CHKB, C3orf58, PLOD3, AIRE, MYT1, Menin, Semaphorin 3A, 8ODP, Connexin 43, HDL proteins, SIX5, DNER, KCRB, Osteonectin, 14-3-3 epsilon, CLIC4, NFIB, hnRNP K, CTGF, LAMB1, PDZ-GEF1, Pirin, Galpha(i)-specific EDG GPCRs, MyHC, Tropomyosin, ITGAV, EGR2 (Krox20), CYP26A1, BMP receptor 2, Ankyrin-B, MIP26,	1.174E-07	67	Fjx1, Tissue kallikreins, Beta-2-microglobulin, Ectodin, IRX2, Histone H2, COL5A2, PAFAH gamma, LIPA, Shc, Histone H2A, C3orf58, PLOD3, Carbonic anhydrase II, Plastin, BDNF, HAS, ZFP36L2, AIRE, SFRP4, Pleiotrophin (OSF1), Menin, Annexin I, T-plastin, Collagen V, HDL proteins, IBP5, 14-3-3 epsilon, GADD45 gamma, BMP5, LAMB1, PDZ-GEF1, NEFM, Nestin, SPRY1, PKC, BMP7, EGR2 (Krox20), CYP26A1, K-cadherin (CDH6), ICAP-1, C/EBP, Ankyrin-B, Fibrillin 2, COL11A1, LIG-1, Adrenomedullin, ASSY, Mab21l2, ZNF148, Fibrillin, AP1S2, G6PD, Nectin-3, LIFR, hnRNP A1, Heme oxygenase 1, Rod1, AP-1 sigma subunits, IBP, Caveolin-1, C/EBPdelta, COL1A2, Osteopontin, IFITM3, 14-3-3, H2AFY2

				LLGL1, Alpha-actinin 1, ASSY, Histone H1, Fibrillin, AP1S2, BETA3, MALT1, INSIG1, AP-1 sigma subunits, Ephrin-B, WNT, NF-I, Osteopontin, Alpha-internexin, SMRT, Midkine, Tropomyosin-1, 14-3-3, IGFBP7/8, H2AFY2, Galpha(q)-specific EDG GPCRs			
7. Single organism developmental process							
7467	3.725E-17	3.725E-17	190	METRNL, LITAF, SPAG16, Ephrin-B3, Fjx1, Alpha-actinin, c-Maf, SOCS2, ARID5B, Ectodin, RhoJ, IRX2, Histone H2, Aurora-B, NAV2, COL5A2, LIPA, NEBL, Histone H2A, PHLDA1, LRRN3, MYH9, Catalase, Carbonic anhydrase II, Plastin, BDNF, SMYD3, SFRP4, LPA1 receptor, LRFN5, Pleiotrophin (OSF1), MKP-3, TXNIP (VDUP1), Annexin I, T-plastin, TSLC1, TMEFF2, Collagen V, CIA/ASF1, APOE, IBP5, ARS2, GADD45 gamma, MYADM, BMP5, ARGBP2, PHF14, LBH, NEFM, ADAM19, SPRY1, FUT8, DCOR, BMP7, Lpd, CEL, FZD1, Myosin I, RBM4, MIAT, SULF1, Tau (MAPT), DAN, Fibrillin 2, COL11A1, LIG-1, SMYD2, TPI1, FGF12, SLC4A7, Adrenomedullin, ID2, TIM, Mab21l2, VGF, ROBO3, Irx6, Wee1, CD24, ID3, G6PD, Synaptotagmin IV, hnRNP A1, DPPA4, 14-3-3 gamma, Creatine kinase, MA2A1, WNT3, Synaptotagmin, PIRB, MRLC2,	1.062E-08	101	BAF47, LITAF, Fjx1, Tissue kallikreins, Beta-2-microglobulin, Ectodin, DR6(TNFRSF21), IRX2, Histone H2, NAV2, COL5A2, PKC-delta, PAFAH gamma, LIPA, Shc, PRNP, SDFR1, Histone H2A, JNK(MAPK8-10), LRRN3, C3orf58, PLOD3, Carbonic anhydrase II, Plastin, BDNF, HAS, ZFP36L2, AIRE, Kindlin-2, SFRP4, Pleiotrophin (OSF1), NRF2, Menin, MKP-3, Annexin I, T-plastin, TMEFF2, Collagen V, HDL proteins, APOE, MSK2, IBP5, ARS2, 14-3-3 epsilon, GADD45 gamma, BMP5, BEX1, LAMB1, PDZ-GEF1, NEFM, Nestin, ARP2, SPRY1, PKC, Fibulin-1, BMP7, EGR2 (Krox20), Lpd, CEL, CYP26A1, K-cadherin (CDH6), ICAP-1, C/EBP, Ankyrin-B, Fibrillin 2, COL11A1, LIG-1, Osteocrin, Adrenomedullin, ASSY,

MRCL3, Heme oxygenase 1, R-cadherin, Rod1, PTPR-zeta, IBP, ASNS, COL1A2, FN14(TNFRSF12A), Cytohesin2, Neurogenin 2, PP2A catalytic, FLRT3, Carboxypeptidase H, MRLC, CD147, EGR1, Frizzled, FNDC3B, Shc, PRNP, SDFR1, CHKB, C3orf58, BRM, Shootin1 (KIAA1598), PLOD3, PTOP, AIRE, Kindlin-2, MYT1, NRF2, Menin, Semaphorin 3A, 8ODP, MYO1B, Connexin 43, HDL proteins, SIX5, DNER, KCRB, Osteonectin, 14-3-3 epsilon, SPRY4, CLIC4, NFIB, hnRNP K, CTGF, LAMB1, PDZ-GEF1, Pirin, ARP2, Galpha(i)-specific EDG GPCRs, MyHC, Tropomyosin, ITGAV, EGR2 (Krox20), Carbohydrate sulfotransferases, CYP26A1, BMP receptor 2, Ankyrin-B, EMP1 (Tmp), MIP26, LLGL1, Osteocrin, STMN2, Alpha-actinin 1, ASSY, Secretogranin II, Histone H1, Fibrillin, AP1S2, Ca-ATPase2, MMD, ERM, BETA3, MALT1, IBP3, CLN2 (Tripeptidyl-peptidase I), CRABP1, INSIG1, AP-1 sigma subunits, Ephrin-B, WNT, CD166, LAMG1, NF-I, Osteopontin, Alpha-internexin, FNBP1, SMRT, Midkine, IRS6(DOK5), Tropomyosin-1, 14-3-3, IGFBP7/8, H2AFY2, Galpha(q)-specific EDG GPCRs, M6B

Secretogranin II, Mab21l2, ZNF148, MSK1/2 (RPS6KA5/4), NQO1, Fibrillin, AP1S2, MID1, ERM, G6PD, IBP3, Nectin-3, LIFR, hnRNP A1, DEC1 (Stra13), Heme oxygenase 1, Rod1, AP-1 sigma subunits, ZC3HDC5, IBP, HOOK1, CD166, LAMG1, Caveolin-1, C/EBPdelta, COL1A2, Osteopontin, IFITM3, FN14(TNFRSF12A), 14-3-3, H2AFY2

8. Nervous system development

3366	6.123E-17	6.123E-17	113	Ephrin-B3, Alpha-actinin, SOCS2, Histone H2, NAV2, COL5A2, Histone H2A, PHLDA1, LRRN3, MYH9, BDNF, LPA1 receptor, LRFN5, Pleiotrophin (OSF1), MKP-3, Annexin I, T-plastin, TSLC1, Collagen V, APOE, ARS2, BMP5, NEFM, BMP7, Lpd, CEL, FZD1, Myosin I, MIAT, SULF1, Tau (MAPT), DAN, LIG-1, FGF12, SLC4A7, Adrenomedullin, ID2, Mab21l2, VGF, ROBO3, Irx6, Wee1, CD24, ID3, G6PD, Synaptotagmin IV, 14-3-3 gamma, Creatine kinase, MA2A1, WNT3, Synaptotagmin, MRLC2, MRCL3, R-cadherin, PTPR-zeta, FN14(TNFRSF12A), Cytohesin2, Neurogenin 2, FLRT3, MRLC, EGR1, Frizzled, Shc, PRNP, SDFR1, BRM, Shootin1 (KIAA1598), PLOD3, MYT1, NRF2, Menin, Semaphorin 3A, MYO1B, Connexin 43, HDL proteins, DNER, KCRB, 14-3-3 epsilon, NFIB, hnRNP K, LAMB1, PDZ-GEF1, ARP2, Galpha(i)-specific EDG GPCRs, MyHC, Tropomyosin, ITGAV, EGR2 (Krox20), Carbohydrate sulfotransferases, CYP26A1, BMP receptor 2, Ankyrin-B, LLGL1, STMN2, MMD, ERM, BETA3, CLN2 (Tripeptidyl-peptidase I), Ephrin-B, WNT, CD166, LAMG1, NF-I, Osteopontin, Alpha-internexin,	5.523E-09	60	BAF47, Tissue kallikreins, Beta-2-microglobulin, DR6(TNFRSF21), Histone H2, NAV2, COL5A2, PAFAH gamma, Shc, PRNP, SDFR1, Histone H2A, JNK(MAPK8-10), LRRN3, PLOD3, BDNF, Pleiotrophin (OSF1), NRF2, Menin, MKP-3, Annexin I, T-plastin, Collagen V, HDL proteins, APOE, MSK2, ARS2, 14-3-3 epsilon, BMP5, BEX1, LAMB1, PDZ-GEF1, NEFM, Nestin, ARP2, PKC, BMP7, EGR2 (Krox20), Lpd, CEL, CYP26A1, C/EBP, Ankyrin-B, LIG-1, Adrenomedullin, Mab21l2, ZNF148, MSK1/2 (RPS6KA5/4), ERM, G6PD, LIFR, DEC1 (Stra13), ZC3HDC5, CD166, LAMG1, Caveolin-1, Osteopontin, FN14(TNFRSF12A), 14-3-3, H2AFY2

					FNBP1, SMRT, Midkine, IRS6(DOK5), 14-3-3, H2AFY2, Galpha(q)-specific EDG GPCRs, M6B			
9. Developmental process								
	7560	6.232E-17	6.232E-17	191	METRNL, LITAF, SPAG16, Ephrin-B3, Fjx1, Alpha-actinin, c-Maf, SOCS2, ARID5B, Ectodin, RhoJ, IRX2, Histone H2, Aurora-B, NAV2, COL5A2, LIPA, NEBL, Histone H2A, PHLDA1, LRRN3, MYH9, Catalase, Carbonic anhydrase II, Plastin, BDNF, SMYD3, SFRP4, LPA1 receptor, LRFN5, Pleiotrophin (OSF1), MKP-3, TXNIP (VDUP1), Annexin I, T-plastin, TSLC1, TMEFF2, Collagen V, CIA/ASF1, APOE, IBP5, ARS2, GADD45 gamma, MYADM, BMP5, ARGBP2, PHF14, LBH, NEFM, ADAM19, SPRY1, FUT8, DCOR, BMP7, Lpd, CEL, IEX1, FZD1, Myosin I, RBM4, MIAT, SULF1, Tau (MAPT), DAN, Fibrillin 2, COL11A1, LIG-1, SMYD2, TPI1, FGF12, SLC4A7, Adrenomedullin, ID2, TIM, Mab21l2, VGF, ROBO3, Irx6, Wee1, CD24, ID3, G6PD, Synaptotagmin IV, hnRNP A1, DPPA4, 14-3-3 gamma, Creatine kinase, MA2A1, WNT3, Synaptotagmin, PIRB, MRLC2, MRCL3, Heme oxygenase 1, R-cadherin, Rod1, PTPR-zeta, IBP, ASNS, COL1A2, FN14(TNFRSF12A), Cytohesin2, Neurogenin 2, PP2A	3.980E-09	103	BAF47, LITAF, Fjx1, Tissue kallikreins, Beta-2-microglobulin, Ectodin, DR6(TNFRSF21), IRX2, Histone H2, NAV2, COL5A2, PKC-delta, PAFAH gamma, LIPA, Shc, PRNP, SDFR1, Histone H2A, JNK(MAPK8-10), LRRN3, C3orf58, PLOD3, Carbonic anhydrase II, Plastin, BDNF, HAS, ZFP36L2, AIRE, Kindlin-2, SFRP4, Pleiotrophin (OSF1), NRF2, Menin, MKP-3, Annexin I, T-plastin, TMEFF2, Collagen V, HDL proteins, APOE, MSK2, IBP5, ARS2, 14-3-3 epsilon, GADD45 gamma, BMP5, BEX1, LAMB1, PDZ-GEF1, NEFM, Nestin, UGCG, ARP2, SPRY1, PKC, Fibulin-1, BMP7, EGR2 (Krox20), Lpd, CEL, IEX1, CYP26A1, K-cadherin (CDH6), ICAP-1, C/EBP, Ankyrin-B, Fibrillin 2, COL11A1, LIG-1, Osteocrin, Adrenomedullin, ASSY, Secretogranin II, Mab21l2, ZNF148, MSK1/2 (RPS6KA5/4), NQO1, Fibrillin, AP1S2, MID1, ERM, G6PD,

catalytic, FLRT3, Carboxypeptidase H, MRLC, CD147, EGR1, Frizzled, FNDC3B, Shc, PRNP, SDFR1, CHKB, C3orf58, BRM, Shootin1 (KIAA1598), PLOD3, PTOP, AIRE, Kindlin-2, MYT1, NRF2, Menin, Semaphorin 3A, 8ODP, MYO1B, Connexin 43, HDL proteins, SIX5, DNER, KCRB, Osteonectin, 14-3-3 epsilon, SPRY4, CLIC4, NFIB, hnRNP K, CTGF, LAMB1, PDZ-GEF1, Pirin, ARP2, Galpha(i)-specific EDG GPCRs, MyHC, Tropomyosin, ITGAV, EGR2 (Krox20), Carbohydrate sulfotransferases, CYP26A1, BMP receptor 2, Ankyrin-B, EMP1 (Tmp), MIP26, LLGL1, Osteocrin, STMN2, Alpha-actinin 1, ASSY, Secretogranin II, Histone H1, Fibrillin, AP1S2, Ca-ATPase2, MMD, ERM, BETA3, MALT1, IBP3, CLN2 (Tripeptidyl-peptidase I), CRABP1, INSIG1, AP-1 sigma subunits, Ephrin-B, WNT, CD166, LAMG1, NF-I, Osteopontin, Alpha-internexin, FNBP1, SMRT, Midkine, IRS6(DOK5), Tropomyosin-1, 14-3-3, IGFBP7/8, H2AFY2, Galpha(q)-specific EDG GPCRs, M6B

IBP3, Nectin-3, LIFR, hnRNP A1, DEC1 (Stra13), Heme oxygenase 1, Rod1, AP-1 sigma subunits, ZC3HDC5, IBP, HOOK1, CD166, LAMG1, Caveolin-1, C/EBPdelta, COL1A2, Osteopontin, IFITM3, FN14(TNFRSF12A), 14-3-3, H2AFY2

10. Cell differentiation

4817	4.489E-16	4.489E-16	140	METRNL, SPAG16, Ephrin-B3, Alpha-actinin, c-Maf, SOCS2, ARID5B, Histone H2, COL5A2, NEBL, Histone H2A, PHLDA1, LRRN3, MYH9, Catalase, Carbonic anhydrase II, BDNF, SMYD3, SFRP4, LPA1 receptor, LRFN5, Pleiotrophin (OSF1), MKP-3, TXNIP (VDUP1), Annexin I, T-plastin, TSLC1, Collagen V, CIA/ASF1, APOE, IBP5, ARS2, GADD45 gamma, MYADM, BMP5, ARGBP2, NEFM, BMP7, Lpd, FZD1, Myosin I, RBM4, MIAT, SULF1, Tau (MAPT), DAN, COL11A1, SLC4A7, Adrenomedullin, ID2, VGF, ROBO3, Irx6, Wee1, CD24, ID3, G6PD, Synaptotagmin IV, 14-3-3 gamma, MA2A1, WNT3, Synaptotagmin, PIRB, MRLC2, MRCL3, R-cadherin, Rod1, PTPR-zeta, IBP, FN14(TNFRSF12A), Cytohesin2, Neurogenin 2, FLRT3, MRLC, EGR1, Frizzled, FNDC3B, Shc, PRNP, SDFR1, BRM, Shootin1 (KIAA1598), PLOD3, AIRE, Kindlin-2, MYT1, NRF2, Menin, Semaphorin 3A, Connexin 43, HDL proteins, SIX5, DNER, 14-3-3 epsilon, CLIC4, NFIB, hnRNP K, CTGF, LAMB1, PDZ-GEF1, Pirin, ARP2, Galpha(i)-specific EDG GPCRs, MyHC, Tropomyosin, ITGAV, EGR2 (Krox20), Carbohydrate	3.536E-07	71	BAF47, Tissue kallikreins, Beta-2-microglobulin, DR6(TNFRSF21), Histone H2, COL5A2, Shc, PRNP, SDFR1, Histone H2A, JNK(MAPK8-10), LRRN3, PLOD3, Carbonic anhydrase II, BDNF, HAS, ZFP36L2, AIRE, Kindlin-2, SFRP4, Pleiotrophin (OSF1), NRF2, Menin, MKP-3, Annexin I, T-plastin, Collagen V, HDL proteins, APOE, MSK2, IBP5, ARS2, 14-3-3 epsilon, GADD45 gamma, BMP5, BEX1, LAMB1, PDZ-GEF1, NEFM, Nestin, ARP2, PKC, BMP7, EGR2 (Krox20), Lpd, CYP26A1, ICAP-1, C/EBP, Ankyrin-B, COL11A1, Osteocrin, Adrenomedullin, MSK1/2 (RPS6KA5/4), AP1S2, ERM, G6PD, IBP3, LIFR, Rod1, AP-1 sigma subunits, ZC3HDC5, IBP, HOOK1, CD166, LAMG1, Caveolin-1, C/EBPdelta, Osteopontin, IFITM3, FN14(TNFRSF12A), 14-3-3

sulfotransferases, CYP26A1, BMP receptor 2, Ankyrin-B, LLGL1, Osteocrin, STMN2, Alpha-actinin 1, Histone H1, AP1S2, Ca-ATPase2, MMD, ERM, BETA3, MALT1, IBP3, CLN2 (Tripeptidyl-peptidase I), AP-1 sigma subunits, Ephrin-B, WNT, CD166, LAMG1, NF-I, Osteopontin, Alpha-internexin, FNBP1, SMRT, Midkine, Tropomyosin-1, 14-3-3, IGFBP7/8, Galpha(q)-specific EDG GPCRs, M6B

Enrichment by GO Processes				Mock ApoE4/Mock no ApoE			Mock ApoE3/Mock no ApoE		
#	Processes	Total	min(p-value)	p-value	In Data	Network Objects from Active Data	p-value	In Data	Network Objects from Active Data
1.	System development								
		5860	1.706E-19	1.706E-19	149	OC-2(-1.25), Annexin II(-1.45), Ephrin-B3(1.55), COL1A1(-1.27), SOCS2(-1.76), XRCC2(-1.48), DR6(TNFRSF21)(-1.45), IGSF3(-1.27), ProSAAS, IRX2(1.32), NAV2(-1.29), LIPA(-1.31), NEBL(1.36), JNK(MAPK8-10)(1.39), G-protein alpha-11(1.26), LRRN3(-1.37), Catalase(1.25), WNT5A(1.26), KIDINS220(-1.3), Plastin(-1.23), Pleiotrophin (OSF1)(-1.25), MKP-3(-1.35), Annexin I(-1.42), FOXC1/2(-1.37), VCAM1(-1.49), EID2B(-1.41), TOP2 beta(1.29), BMP5(1.59), Fatty acid-binding protein(-1.34), NEFM(-	3.180E-12	64	PTK7(1.27), CYP26A1(1.41), MRLC, Ephrin-B3(1.36), Gamma-sarcoglycan, Beta-2-microglobulin(-1.25), HLA-A2(-1.25), SOCS2(-1.43), HMG1,2(-1.25), LIG-1, RDC1(-1.34), HLA-A(-1.25), LLGL1(1.25), XRCC2(-1.39), APH-1A(1.28), STMN2(-1.69), HES5(-1.42), LRP4(1.28), Mab21l2(-1.44), G-protein alpha-11(1.325), LRRN3(-1.28), Semaphorin 3E(-1.39), BAT3(1.26), USF2(1.25), Wee1(-1.28), Carbonic

1.51), COL4A1(-1.52), hnRNP U(-1.32), CREB1(-1.32), Prdm8(-1.3), WNT3A(1.54), ODZ2, CEL, FZD1(1.31), C/EBP, Tau (MAPT)(-1.4), DAN, Homer(-1.25), COL11A1(-1.31), LIG-1, Adrenomedullin(-1.55), ID2(1.32), TLE1(-1.27), RPA1(1.27), Mab21l2(-1.72), Transgelin(-1.27), Irx6(1.39), Galpha(q)-specific peptide GPCRs, FOXC1 (-1.37), Synaptotagmin IV (-2.37), DPPA4(1.4), REDD1(1.31), WNT3(1.54), Synaptotagmin(2.37), MRLC2, NEDD4, MRCL3(-1.37), Doublecortin(-1.83), Caspr2(-1.31), B-FABP(-1.34), ODZ1, Thrombospondin 1(1.37), PTPR-zeta(1.33), IBP, AMELY, FGF10(1.28), ATF/CREB(-1.32), SEZ6L2(-1.3), COL1A2(-1.36), FN14(TNFRSF12A)(-1.29), CRP3 (MLP)(-1.28), ID1(1.38), MAP-1B(1.27), Neurogenin 2(-2.1), AFP(-1.28), FLRT3(-1.45), PTK7(1.27), p57(-1.33), Carboxypeptidase H, MRLC, ZIC2(1.3), ACTA2(-1.52), Contactin 2(-1.35), Frizzled, METRN(1.32), BAT3, ENC1, SMAD6(1.25), Collagen I(-1.27), Metallothionein-I(-1.3), HuD (ELAVL-4)(-1.27), MAP4(-1.39), MYT1(-1.56), Menin, Semaphorin 3A (1.5), UNC45A(1.25), SHRM(1.25), ODZ3(1.37),

anhydrase II(-1.22), PTB2, Synaptotagmin IV(-1.55), SFRP4(1.33), MYT1(-1.3), REDD1(1.31), WNT3(1.27), Synaptotagmin, SLC4A2, MKP-3(-1.26), MRLC2, MRCL3(-1.28), HMG2(-1.26), Semaphorin 3A(1.36), APH-1(1.28), ODZ1, G-protein alpha-q/11(1.32), VCAM1(-1.33), Thrombospondin 1(1.41), ODZ3(1.34), HLA-A68, EID2B, HLA-A11, PTPR-zeta(1.34), CORIN, Ephrin-B, WNT, 14-3-3 epsilon, TISB(-1.3), BMP5(1.29), ATF/CREB, 14-3-3, CREB1(-1.25), Neurogenin 2(-1.43), nAChR alpha(-1.34), ODZ2, HLA-A3, MHC class I, SFRP1(-1.26)

SIX5(1.32), Actin muscle, DNER(-1.31), STAT1(1.3), 14-3-3 epsilon(1.58), FGFR3(1.26), CTGF(-1.26), TOP2(1.29), TLE(-1.27), Tropomyosin(-1.35), nAChR alpha(-1.31), EGR2 (Krox20)(-1.29), SFRP1(-1.56), CYP26A1(1.48), K-cadherin (CDH6)(-1.28), Gamma-sarcoglycan, SFRP2(1.45), G-protein gamma(-1.26), MIP26, NAP1(1.26), STMN2(-2.8), ASSY(-1.3), Secretogranin II(-1.52), HES5(-2.2), PREX1(1.26), BETA3, LMO4(-1.3), SOX3(-1.59), SNAP-25(-1.37), G-protein alpha-q/11(1.26), DLL3(-1.28, SLC7A5(-1.43), Ephrin-B(1.55), WNT, CD166(-1.56), C/EBPdelta(-1.28), Actin, Osteopontin(-1.33), Alpha-internexin(-1.56), Uronyl-2-sulfotransferase(1.28), NHLH2(-1.25), Collagen IV(-1.5), Tropomyosin-1(-1.35), 14-3-3, IGFBP7/8, HEPH(1.28), M6B(1.26)

2. Single-multicellular organism process

	7650	3.822E-19	3.822E-19	175	OC-2, Annexin II, Ephrin-B3, COL1A1, SOCS2, XRCC2, DR6(TNFRSF21), IGSF3, ProSAAS, IRX2, NAV2, LIPA, NEBL, JNK(MAPK8-10), G-protein alpha-11, LRRN3, Optineurin, Catalase, WNT5A, KIDINS220, Plastin, Pleiotrophin (OSF1), G-protein gamma 2, MKP-3, Annexin I, FOXC1/2, VCAM1, EID2B, TOP2 beta, IRF7, BMP5, Fatty acid-binding protein, LBH, NEFM, COL4A1, hnRNP U, CREB1, Prdm8, WNT3A, ODZ2, CEL, FZD1, TMEM46, C/EBP, Tau (MAPT), DAN, Homer, COL11A1, LIG-1, TPI1, Adrenomedullin, ID2, TLE1, Multimerin, RPA1, nAChR alpha-5, Mab21l2, Transgelin, TFPI-2, Irx6, LIPE, Galpha(q)-specific peptide GPCRs, FOXC1, Synaptotagmin IV, DPPA4, Tubulin alpha, REDD1, WNT3, Synaptotagmin, MRLC2, NEDD4, MRCL3, Doublecortin, Caspr2, B-FABP, ODZ1, Thrombospondin 1, PTPR-zeta, IBP, AMELY, FGF10, ATF/CREB, SEZ6L2, COL1A2, Secretogranin III, FN14(TNFRSF12A), CRP3 (MLP), ID1, MAP-1B, Neurogenin 2, AFP, FLRT3, PTK7, p57,	4.226E-12	74	PTK7, EBF3, CYP26A1, MRLC, ATP1B3, Ephrin-B3, Gamma-sarcoglycan, Beta-2-microglobulin, HLA-A2, SOCS2, HMG1,2, LIG-1, RDC1, HLA-A, LLGL1, XRCC2, APH-1A, Multimerin, STMN2, HES5, nAChR alpha-5, LRP4, Mab21l2, G-protein alpha-11, LRRN3, Semaphorin 3E, BAT3, USF2, Wee1, Carbonic anhydrase II, PTB2, Synaptotagmin IV, SFRP4, MYT1, TDRD1, REDD1, WNT3, Synaptotagmin, SLC4A2, MKP-3, MRLC2, MRCL3, HMG2, Semaphorin 3A, APH-1, ODZ1, G-protein alpha-q/11, VCAM1, Thrombospondin 1, ODZ3, EBF4, HLA-A68, EID2B, HLA-A11, PTPR-zeta, CORIN, Ephrin-B, WNT, 14-3-3 epsilon, TISB, BMP5, CALP, ATF/CREB, Secretogranin III, 14-3-3, CREB1, Fibulin-1, Neurogenin 2, nAChR alpha, ODZ2, AMYS, HLA-A3, MHC class I, SFRP1

Carboxypeptidase H, MRLC, ZIC2, ACTA2, Contactin 2, Frizzled, METRN, BAT3, ENC1, SMAD6, Collagen I, Metallothionein-I, HuD (ELAVL-4), MAP4, MYT1, LPL, Menin, Semaphorin 3A, UNC45A, SHRM, ODZ3, EBF4, SIX5, Actin muscle, DNER, STAT1, 14-3-3 epsilon, FGFR3, CTGF, SCN7A, TOP2, TLE, Tropomyosin, nAChR alpha, EGR2 (Krox20), SFRP1, EBF3, CYP26A1, ATP1B3, K-cadherin (CDH6), Homer 2, Gamma-sarcoglycan, SFRP2, G-protein gamma, EMP1 (Tmp), MIP26, NAP1, STMN2, ASSY, Secretogranin II, HES5, PREX1, Ca-ATPase2, BETA3, IBP3, Raftlin, LMO4, CRABP1, SOX3, SNAP-25, G-protein alpha-q/11, DLL3, SLC7A5, PCDH8, Ephrin-B, Sp8, WNT, CD166, CALP, C/EBPdelta, Actin, Osteopontin, Alpha-internexin, Uronyl-2-sulfotransferase, NHLH2, Collagen IV, Tropomyosin-1, 14-3-3, IGFBP7/8, HEPH, M6B

3. Nervous system development

	3366	9.991E-19	9.991E-19	105	OC-2, Ephrin-B3, SOCS2, XRCC2, DR6(TNFRSF21), ProSAAS, NAV2, JNK(MAPK8-10), LRRN3, WNT5A, KIDINS220, Pleiotrophin (OSF1), MKP-3, Annexin I, FOXC1/2, TOP2 beta, BMP5, Fatty acid-binding protein, NEFM, COL4A1, CREB1, Prdm8, WNT3A, ODZ2, CEL, FZD1, C/EBP, Tau (MAPT), DAN, LIG-1, Adrenomedullin, ID2, Mab21l2, Irx6, Galpha(q)-specific peptide GPCRs, FOXC1, Synaptotagmin IV, REDD1, WNT3, Synaptotagmin, MRLC2, NEDD4, MRCL3, Doublecortin, Caspr2, B-FABP, ODZ1, PTPR-zeta, FGF10, ATF/CREB, SEZ6L2, FN14(TNFRSF12A), ID1, MAP-1B, Neurogenin 2, FLRT3, PTK7, p57, MRLC, ZIC2, Contactin 2, Frizzled, METRN, BAT3, ENC1, HuD (ELAVL-4), MAP4, MYT1, Menin, Semaphorin 3A, SHRM, ODZ3, DNER, 14-3-3 epsilon, FGFR3, TOP2, Tropomyosin, nAChR alpha, EGR2 (Krox20), SFRP1, CYP26A1, SFRP2, G-protein gamma, NAP1, STMN2, HES5, PREX1, BETA3, LMO4, SOX3, SNAP-25, G-protein alpha-q/11, DLL3, SLC7A5, Ephrin-B, WNT, CD166, Actin, Osteopontin, Alpha-	8.461E-17	54	PTK7, CYP26A1, MRLC, Ephrin-B3, Beta-2-microglobulin, HLA-A2, SOCS2, HMG1,2, LIG-1, HLA-A, LLGL1, XRCC2, APH-1A, STMN2, HES5, LRP4, Mab21l2, LRRN3, Semaphorin 3E, BAT3, Wee1, PTB2, Synaptotagmin IV, MYT1, REDD1, WNT3, Synaptotagmin, MKP-3, MRLC2, MRCL3, HMG2, Semaphorin 3A, APH-1, ODZ1, G-protein alpha-q/11, ODZ3, HLA-A68, HLA-A11, PTPR-zeta, CORIN, Ephrin-B, WNT, 14-3-3 epsilon, TISB, BMP5, ATF/CREB, 14-3-3, CREB1, Neurogenin 2, nAChR alpha, ODZ2, HLA-A3, MHC class I, SFRP1

					internexin, Uronyl-2-sulfotransferase, NHLH2, Collagen IV, 14-3-3, M6B			
4. Multicellular organism development								
	6641	3.992E-18	3.992E-18	158	OC-2, Annexin II, Ephrin-B3, COL1A1, SOCS2, XRCC2, DR6(TNFRSF21), IGSF3, ProSAAS, IRX2, NAV2, LIPA, NEBL, JNK(MAPK8-10), G-protein alpha-11, LRRN3, Catalase, WNT5A, KIDINS220, Plastin, Pleiotrophin (OSF1), MKP-3, Annexin I, FOXC1/2, VCAM1, EID2B, TOP2 beta, BMP5, Fatty acid-binding protein, LBH, NEFM, COL4A1, hnRNP U, CREB1, Prdm8, WNT3A, ODZ2, CEL, FZD1, TMEM46, C/EBP, Tau (MAPT), DAN, Homer, COL11A1, LIG-1, TPI1, Adrenomedullin, ID2, TLE1, RPA1, Mab21l2, Transgelin, Irx6, Galpha(q)-specific peptide GPCRs, FOXC1, Synaptotagmin IV, DPPA4, REDD1, WNT3, Synaptotagmin, MRLC2, NEDD4, MRCL3, Doublecortin, Caspr2, B-FABP, ODZ1, Thrombospondin 1, PTPR-zeta, IBP, AMELY, FGF10, ATF/CREB, SEZ6L2, COL1A2, FN14(TNFRSF12A), CRP3 (MLP), ID1, MAP-1B, Neurogenin 2, AFP, FLRT3, PTK7, p57, Carboxypeptidase H, MRLC, ZIC2, ACTA2, Contactin 2, Frizzled,	7.126E-12	68	PTK7, EBF3, CYP26A1, MRLC, Ephrin-B3, Gamma-sarcoglycan, Beta-2-microglobulin, HLA-A2, SOCS2, HMG1,2, LIG-1, RDC1, HLA-A, LLGL1, XRCC2, APH-1A, STMN2, HES5, LRP4, Mab21l2, G-protein alpha-11, LRRN3, Semaphorin 3E, BAT3, USF2, Wee1, Carbonic anhydrase II, PTB2, Synaptotagmin IV, SFRP4, MYT1, TDRD1, REDD1, WNT3, Synaptotagmin, SLC4A2, MKP-3, MRLC2, MRCL3, HMG2, Semaphorin 3A, APH-1, ODZ1, G-protein alpha-q/11, VCAM1, Thrombospondin 1, ODZ3, EBF4, HLA-A68, EID2B, HLA-A11, PTPR-zeta, CORIN, Ephrin-B, WNT, 14-3-3 epsilon, TISB, BMP5, ATF/CREB, 14-3-3, CREB1, Fibulin-1, Neurogenin 2, nAChR alpha, ODZ2, HLA-A3, MHC class I, SFRP1

				METRN, BAT3, ENC1, SMAD6, Collagen I, Metallothionein-I, HuD (ELAVL-4), MAP4, MYT1, Menin, Semaphorin 3A, UNC45A, SHRM, ODZ3, EBF4, SIX5, Actin muscle, DNER, STAT1, 14-3-3 epsilon, FGFR3, CTGF, TOP2, TLE, Tropomyosin, nAChR alpha, EGR2 (Krox20), SFRP1, EBF3, CYP26A1, K-cadherin (CDH6), Gamma-sarcoglycan, SFRP2, G-protein gamma, EMP1 (Tmp), MIP26, NAP1, STMN2, ASSY, Secretogranin II, HES5, PREX1, BETA3, LMO4, CRABP1, SOX3, SNAP-25, G-protein alpha-q/11, DLL3, SLC7A5, PCDH8, Ephrin-B, Sp8, WNT, CD166, C/EBPdelta, Actin, Osteopontin, Alpha-internexin, Uronyl-2-sulfotransferase, NHLH2, Collagen IV, Tropomyosin-1, 14-3-3, IGFBP7/8, HEPH, M6B			
5. Neurogenesis								
	2479	8.807E-18	8.807E-18	86	OC-2, Ephrin-B3, SOCS2, XRCC2, DR6(TNFRSF21), JNK(MAPK8-10), LRRN3, WNT5A, KIDINS220, Pleiotrophin (OSF1), MKP-3, Annexin I, TOP2 beta, BMP5, Fatty acid-binding protein, NEFM, COL4A1, CREB1, Prdm8, WNT3A, ODZ2, FZD1, C/EBP, Tau (MAPT), DAN, Adrenomedullin, ID2, Irx6, Galpha(q)-specific peptide GPCRs, Synaptotagmin IV, REDD1, WNT3,	2.494E-16	46	PTK7, MRLC, Ephrin-B3, Beta-2-microglobulin, HLA-A2, SOCS2, HMG1,2, HLA-A, LLGL1, XRCC2, APH-1A, STMN2, HES5, LRP4, LRRN3, Semaphorin 3E, Wee1, Synaptotagmin IV, REDD1, WNT3, Synaptotagmin, MKP-3, MRLC2, MRCL3, HMG2, Semaphorin 3A, APH-1, G-protein alpha-q/11, ODZ3,

					Synaptotagmin, MRLC2, NEDD4, MRCL3, Doublecortin, Caspr2, B-FABP, PTPR-zeta, FGF10, ATF/CREB, FN14(TNFRSF12A), ID1, MAP-1B, Neurogenin 2, FLRT3, PTK7, p57, MRLC, ZIC2, Contactin 2, Frizzled, METRN, ENC1, HuD (ELAVL-4), MAP4, Semaphorin 3A, ODZ3, DNER, 14-3-3 epsilon, FGFR3, TOP2, nAChR alpha, EGR2 (Krox20), SFRP1, SFRP2, G-protein gamma, NAP1, STMN2, HES5, PREX1, BETA3, LMO4, SOX3, SNAP-25, G-protein alpha-q/11, DLL3, Ephrin-B, WNT, CD166, Actin, Osteopontin, Uronyl-2-sulfotransferase, Collagen IV, 14-3-3			HLA-A68, HLA-A11, PTPR-zeta, CORIN, Ephrin-B, WNT, 14-3-3 epsilon, BMP5, ATF/CREB, 14-3-3, CREB1, Neurogenin 2, nAChR alpha, ODZ2, HLA-A3, MHC class I, SFRP1
6. Animal organ development								
	4305	2.193E-17	2.193E-17	119	OC-2, Annexin II, COL1A1, SOCS2, IGSF3, ProSAAS, IRX2, LIPA, NEBL, G-protein alpha-11, Catalase, WNT5A, Plastin, Pleiotrophin (OSF1), Annexin I, FOXC1/2, VCAM1, EID2B, TOP2 beta, BMP5, Fatty acid-binding protein, NEFM, COL4A1, hnRNP U, CREB1, Prdm8, WNT3A, FZD1, C/EBP, Tau (MAPT), Homer, COL11A1, LIG-1, Adrenomedullin, ID2, TLE1, RPA1, Mab21l2, Transgelin, Irx6, Galpha(q)-specific peptide GPCRs, FOXC1, Synaptotagmin IV, DPPA4, REDD1, WNT3, Synaptotagmin, NEDD4, Doublecortin, Caspr2, B-	1.756 E-10	51	PTK7, CYP26A1, MRLC, Gamma-sarcoglycan, Beta-2-microglobulin, HLA-A2, SOCS2, HMG1,2, LIG-1, HLA-A, LLGL1, APH-1A, HES5, LRP4, Mab21l2, G-protein alpha-11, Semaphorin 3E, BAT3, USF2, Carbonic anhydrase II, PTB2, Synaptotagmin IV, SFRP4, MYT1, REDD1, WNT3, Synaptotagmin, HMG2, Semaphorin 3A, APH-1, G-protein alpha-q/11, VCAM1, Thrombospondin 1, ODZ3, HLA-A68, EID2B, HLA-A11,

FABP, Thrombospondin 1, PTPR-zeta, IBP, AMELY, FGF10, ATF/CREB, SEZ6L2, COL1A2, CRP3 (MLP), ID1, Neurogenin 2, AFP, FLRT3, PTK7, p57, Carboxypeptidase H, MRLC, ZIC2, ACTA2, Contactin 2, Frizzled, BAT3, SMAD6, Collagen I, Metallothionein-I, MYT1, Menin, Semaphorin 3A, UNC45A, ODZ3, SIX5, Actin muscle, DNER, STAT1, 14-3-3 epsilon, FGFR3, CTGF, TOP2, TLE, Tropomyosin, nAChR alpha, EGR2 (Krox20), SFRP1, CYP26A1, K-cadherin (CDH6), Gamma-sarcoglycan, SFRP2, G-protein gamma, MIP26, ASSY, HES5, PREX1, BETA3, LMO4, SOX3, G-protein alpha-q/11, DLL3, Ephrin-B, WNT, C/EBPdelta, Actin, Osteopontin, Alpha-internexin, Collagen IV, Tropomyosin-1, 14-3-3, IGFBP7/8, HEPH

PTPR-zeta, Ephrin-B, WNT, 14-3-3 epsilon, TISB, BMP5, ATF/CREB, 14-3-3, CREB1, Neurogenin 2, nAChR alpha, HLA-A3, MHC class I, SFRP1

7. Anatomical structure morphogenesis

3251	2.731E-17	2.73 1E-17	100	OC-2, Annexin II, Ephrin-B3, COL1A1, XRCC2, IRX2, LIPA, NEBL, JNK(MAPK8-10), LRRN3, WNT5A, KIDINS220, Plastin, Pleiotrophin (OSF1), MKP-3, FOXC1/2, VCAM1, TMEFF2, TOP2 beta, COL4A1, CREB1, Prdm8, WNT3A, ODZ2, IEX1, FZD1, C/EBP, Tau (MAPT), DAN, COL11A1, LIG-1, Adrenomedullin, ID2, TLE1, Mab21l2, Irx6, Galpha(q)-specific peptide GPCRs, FOXC1, WNT3, MRLC2, NEDD4, MRCL3, Doublecortin, Thrombospondin 1, PTPR-zeta, IBP, AMELY, FGF10, ATF/CREB, COL1A2, FN14(TNFRSF12A), CRP3 (MLP), ID1, MAP-1B, Neurogenin 2, FLRT3, PTK7, p57, Carboxypeptidase H, MRLC, ZIC2, ACTA2, Contactin 2, Frizzled, SMAD6, Collagen I, HuD (ELAVL-4), Menin, Semaphorin 3A, SHRM, ODZ3, Actin muscle, FGFR3, CTGF, TOP2, TLE, Tropomyosin, nAChR alpha, EGR2 (Krox20), SFRP1, SFRP2, G-protein gamma, Secretogranin II, HES5, BETA3, LMO4, SOX3, SNAP-25, G-protein alpha-q/11, DLL3, PCDH8, Ephrin-B, Sp8, WNT, CD166, Actin, Collagen IV, Tropomyosin-1, 14-3-3, IGFBP7/8	5.617 E-08	39	PTK7, MRLC, Ephrin-B3, HMG1,2, LIG-1, RDC1, LLGL1, XRCC2, APH-1A, HES5, LRP4, Mab21l2, LRRN3, Semaphorin 3E, TSGA14, Wee1, Carbonic anhydrase II, SFRP4, WNT3, MKP-3, MRLC2, MRCL3, Semaphorin 3A, APH-1, G-protein alpha-q/11, VCAM1, Thrombospondin 1, ODZ3, PTPR-zeta, Ephrin-B, WNT, TISB, ATF/CREB, 14-3-3, CREB1, Neurogenin 2, nAChR alpha, ODZ2, SFRP1

8. Generation of neurons							
	2361	3.781E-17	6.332E-17	82	OC-2, Ephrin-B3, SOCS2, XRCC2, DR6(TNFRSF21), JNK(MAPK8-10), LRRN3, WNT5A, KIDINS220, Pleiotrophin (OSF1), MKP-3, TOP2 beta, BMP5, NEFM, COL4A1, CREB1, Prdm8, WNT3A, ODZ2, FZD1, C/EBP, Tau (MAPT), DAN, Adrenomedullin, ID2, Irx6, Galpha(q)-specific peptide GPCRs, Synaptotagmin IV, REDD1, WNT3, Synaptotagmin, MRLC2, NEDD4, MRCL3, Doublecortin, Caspr2, PTPR-zeta, FGF10, ATF/CREB, FN14(TNFRSF12A), ID1, MAP-1B, Neurogenin 2, FLRT3, PTK7, p57, MRLC, ZIC2, Contactin 2, Frizzled, METRN, ENC1, HuD (ELAVL-4), MAP4, Semaphorin 3A, ODZ3, DNER, 14-3-3 epsilon, FGFR3, TOP2, nAChR alpha, EGR2 (Krox20), SFRP1, SFRP2, G-protein gamma, STMN2, HES5, PREX1, BETA3, LMO4, SOX3, SNAP-25, G-protein alpha-q/11, DLL3, Ephrin-B, WNT, CD166, Actin, Osteopontin, Uronyl-2-sulfotransferase, Collagen IV, 14-3-3	3.781E-17	46	PTK7, MRLC, Ephrin-B3, Beta-2-microglobulin, HLA-A2, SOCS2, HMG1,2, HLA-A, LLGL1, XRCC2, APH-1A, STMN2, HES5, LRP4, LRRN3, Semaphorin 3E, Wee1, Synaptotagmin IV, REDD1, WNT3, Synaptotagmin, MKP-3, MRLC2, MRCL3, HMG2, Semaphorin 3A, APH-1, G-protein alpha-q/11, ODZ3, HLA-A68, HLA-A11, PTPR-zeta, CORIN, Ephrin-B, WNT, 14-3-3 epsilon, BMP5, ATF/CREB, 14-3-3, CREB1, Neurogenin 2, nAChR alpha, ODZ2, HLA-A3, MHC class I, SFRP1

9. Cell differentiation								
	4817	4.254E-17	4.254E-17	127	OC-2, Annexin II, Ephrin-B3, COL1A1, SOCS2, XRCC2, DR6(TNFRSF21), NEBL, JNK(MAPK8-10), LRRN3, Catalase, WNT5A, KIDINS220, Pleiotrophin (OSF1), MKP-3, Annexin I, FOXC1/2, VCAM1, EID2B, TOP2 beta, BMP5, Fatty acid-binding protein, NEFM, COL4A1, hnRNP U, CREB1, Prdm8, WNT3A, ODZ2, FZD1, C/EBP, Tau (MAPT), DAN, Homer, COL11A1, Adrenomedullin, ID2, Transgelin, Irx6, Galpha(q)-specific peptide GPCRs, FOXC1, Synaptotagmin IV, REDD1, WNT3, Synaptotagmin, MRLC2, NEDD4, MRCL3, Doublecortin, Caspr2, B-FABP, PTPR-zeta, IBP, FGF10, ATF/CREB, FN14(TNFRSF12A), CRP3 (MLP), ID1, MAP-1B, Neurogenin 2, FLRT3, PTK7, p57, MRLC, ZIC2, ACTA2, Contactin 2, Frizzled, METRN, BAT3, ENC1, SMAD6, Collagen I, Metallothionein-I, HuD (ELAVL-4), MAP4, MYT1, Menin, Semaphorin 3A, UNC45A, SHRM, ODZ3, SIX5, Actin muscle, DNER, STAT1, 14-3-3 epsilon, FGFR3, CTGF, TOP2, TLE, Tropomyosin, nAChR alpha, EGR2 (Krox20), SFRP1, CYP26A1, Gamma-sarcoglycan, SFRP2, G-protein gamma, NAP1, STMN2, HES5, PREX1, Ca-ATPase2, BETA3, IBP3,	8.357E-14	60	PTK7, CYP26A1, MRLC, Ephrin-B3, Gamma-sarcoglycan, Beta-2-microglobulin, HLA-A2, SOCS2, HMG1,2, RDC1, HLA-A, LLGL1, XRCC2, APH-1A, STMN2, HES5, LRP4, LRRN3, Semaphorin 3E, BAT3, Wee1, Carbonic anhydrase II, Synaptotagmin IV, SFRP4, MYT1, TDRD1, REDD1, WNT3, Synaptotagmin, MKP-3, MRLC2, MRCL3, HMG2, Semaphorin 3A, APH-1, ZAK, G-protein alpha-q/11, VCAM1, RGS2, ODZ3, TCP1-alpha, HLA-A68, EID2B, HLA-A11, PTPR-zeta, CORIN, Ephrin-B, WNT, 14-3-3 epsilon, TISB, BMP5, ATF/CREB, 14-3-3, CREB1, Neurogenin 2, nAChR alpha, ODZ2, HLA-A3, MHC class I, SFRP1

					LMO4, SOX3, SNAP-25, G-protein alpha-q/11, DLL3, SLC7A5, Ephrin-B, WNT, CD166, C/EBPdelta, Actin, Osteopontin, Alpha-internexin, Uronyl-2-sulfotransferase, NHLH2, Collagen IV, Tropomyosin-1, 14-3-3, IGFBP7/8, HEPH, M6B			
10. Head development								
	1162	8.112E-17	8.112E-17	55	COL1A1, ProSAAS, WNT5A, Pleiotrophin (OSF1), FOXC1/2, TOP2 beta, Fatty acid-binding protein, NEFM, COL4A1, CREB1, Prdm8, WNT3A, FZD1, Tau (MAPT), ID2, Galpha(q)-specific peptide GPCRs, FOXC1, Synaptotagmin IV, REDD1, WNT3, Synaptotagmin, Doublecortin, Caspr2, B-FABP, PTPR-zeta, FGF10, ATF/CREB, SEZ6L2, ID1, Neurogenin 2, FLRT3, ZIC2, Contactin 2, Frizzled, BAT3, Collagen I, Menin, Semaphorin 3A, 14-3-3 epsilon, FGFR3, TOP2, Tropomyosin, EGR2 (Krox20), SFRP1, SFRP2, G-protein gamma, HES5, BETA3, SOX3, G-protein alpha-q/11, WNT, Actin, Alpha-internexin, Collagen IV, 14-3-3	1.709E-05	18	LLGL1, HES5, BAT3, PTB2, Synaptotagmin IV, REDD1, WNT3, Synaptotagmin, Semaphorin 3A, G-protein alpha-q/11, PTPR-zeta, WNT, 14-3-3 epsilon, ATF/CREB, 14-3-3, CREB1, Neurogenin 2, SFRP1

Enrichment by GO Processes				HIV(SF2) +rApoE4/no rApoE			HIV(SF2) +rApoE3/ no rApoE		
#	Processes	Total	Min (p-value)	p-value	In Data	Network Objects from Active Data	p-value	In Data	Network Objects from Active Data
1. Nervous system development									
		3366	2.241E-34	2.241 E-34	138	Ephrin-B3 (1.53), Alpha-N-catenin, Alpha-actinin, SOCS2 (-1.66), HMG1,2, AK3L1 (-1.2), PRICKLE2(-1.27), DR6(TNFRSF21)(-1.5), Paralemmin(1.44), PDE5A, COL5A2, Ephrin-A receptors, JNK(MAPK8-10), LRRN3(-1.3), MYH9(-1.3), MEGF10 (-1.29), BDNF (-1.35), LEC2, LRFN5 (-1.27), Pleiotrophin (OSF1)(-1.41), MKP-3(-1.34), Lamin B (-1.27), FOXC1/2, T-plastin (-1.4), Collagen V(-1.28), APOE(-1.3), ARS2(1.3), BMP5(1.32), NMP200(1.34), NEFM (-1.61), COL4A1(-1.64), WNT3A(1.27), SLIT3, CEL, FZD1(1.29), C/EBP(-1.4), Tau (MAPT)(-1.45), RhoC(-1.22), LIG-1, Adrenomedullin (-1.78), ID2(1.34), BChE(1.28), Mab21l2(-1.73), Irx6(1.38), Galpha(q)-specific peptide GPCRs, ID3(1.29), FOXC1(-167), G6PD (-127), UCHL1(-1.25), Synaptotagmin IV(-2.7), DEC1 (Stra13), MAP6(1.3), REDD1(1.38), WNT3(1.38), Synaptotagmin, NEFL(-1.48), Doublecortin (-1.76), Caspr2(-1.4), HMG2(1.32), Calcineurin B1(-1.29), PDE, PTPR-zeta, ENO (1.25), Alpha-catenin, FN14(TNFRSF12A)(-1.53), ID1(1.4), MAP-1B(1.28), Neurogenin 2(-1.93), CHD8(-1.26), FLRT3(-1.45), PTK7 (1.41), Atlastin,	5.813E-06	26	CEL, CYP26A1(1.41), Ephrin-B3 (1.32), SNAPs, LIG-1, ProSAAS, STMN2(-1.54), Nova1(-1.25), Mab21l2 (-1.39), LRRN3, REDD1(1.33), Pleiotrophin (OSF1)(-1.49), Menin, MKP-3 (-1.28), Semaphorin 3A(1.29), Annexin I (-1.41), Ephrin-B, CD166 (-1.41), 14-3-3 epsilon(1.43), LAMG1, Osteopontin (-1.43), FEZ2, COL4A1 (-1.42), FN14(TNFRSF12A)(-1.28), Collagen IV, 14-3-3

Tissue kallikreins, SNAPs, Stathmin, ZIC2(1.46), Nova1(-1.26), EGR1(-1.38), SLIT2(-1.26), PAFAH gamma(1.26), Contactin 2(-1.38), Frizzled(-1.56), WDR5(1.34), ENC1, CX3CL1(-1.33), Shootin1 (KIAA1598)(-1.32), Galpha(i)-specific peptide GPCRs, MAP4 (-1.46), MYT1(-1.3), DNA-PK, Menin, Semaphorin 3A(1.5), Connexin 43(-1.3), HDL proteins, DNER(-1.37), AK3, FGFR3(1.32), LAMB1(-1.32), Galpha(i)-specific EDG GPCRs, MyHC, Tropomyosin(-1.35), CAPZ beta(-1.38), EGR2 (Krox20)(-1.39), SFRP1(-1.56), Carbohydrate sulfotransferases, CYP26A1(1.23), BMP receptor 2(-1.27), Ephrin-A receptor 4(-1.39), STMN2(-2.67), NAP1(1.26), HES5 (-1.97), CDK1 (p34), MMD, ERM, BETA3, POU class III, RILP (REST-interacting LIM domain protein), POU3F2 (BRN2)(-1.27), Calcineurin B (regulatory)(-1.27), SOX3(-1.78), SNAP-25(-1.31), FGF14(1.39), DLL3(-1.27), SLC7A5 (-1.7), Ephrin-B, EZH2(1.25), WNT, CD166(-1.74), LAMG1, Beta-adaptin 2(-1.53), APP(-1.41), Actin, Osteopontin (-1.58), FEZ2(-1.33), Alpha-internexin(-1.58), Collagen IV, ENO3(1.25), Galpha(q)-specific EDG GPCRs

2. System development

5860	1.283E-32	1.283 E-32	183	Ephrin-B3, Alpha-N-catenin, Alpha-actinin, c-Maf, COL1A1, SOCS2, HMG1,2, AK3L1, PRICKLE2, DR6(TNFRSF21), HSP47, Paralemmin, IRX2, PDE5A, COL5A2, LIPA, Ephrin-A receptors, NEBL, JNK(MAPK8-10), LRRN3, MYH9, MEGF10, Plastin, BDNF, HAS, LEC2, LRFN5, Pleiotrophin (OSF1), MKP-3, Lamin B, FOXC1/2, T-plastin, VCAM1, Collagen V, APOE, ARS2, BMP5, FANCG, NMP200, NEFM, COL4A1, SPRY1, WNT3A, SLIT3, Podocalyxin-like 1, CEL, FZD1, C/EBP, Tau (MAPT), Fibrillin 2, COL11A1, RhoC, LIG-1, Adrenomedullin, ID2, TLE1, TIM, BChE, Mab21l2, Transgelin, Irx6, Galpha(q)-specific peptide GPCRs, ID3, FOXC1, G6PD, UCHL1, Synaptotagmin IV, DPPA4, DEC1 (Stra13), MAP6, REDD1, WNT3, Synaptotagmin, NEFL, Doublecortin, Caspr2, HMG2, Calcineurin B1, PDE, PTPR-zeta, IBP, ASNS, ENO, COL1A2, Alpha-catenin, FN14(TNFRSF12A), FAM65B, ID1, MAP-1B, Neurogenin 2, CHD8, FLRT3, PTK7, Carboxypeptidase H, Atlastin, Tissue kallikreins, SNAPs, A2M, Stathmin, ZIC2, NPY5R, Nova1, EGR1, ACTA2, SLIT2, PAFAH gamma, Contactin 2, Frizzled, WDR5, ENC1, CX3CL1, Shootin1 (KIAA1598), Galpha(i)-specific peptide GPCRs, Collagen I, TAFs, MAP4, MYT1, DNA-PK, Menin, Semaphorin 3A, Antigen KI-67, Connexin 43, HDL proteins, SIX5, Actin muscle, DNER, Osteonectin,	6.17 1E-08	40	Carboxypeptidase H, CEL, CYP26A1, Ephrin-B3, SNAPs, FKBP12, Fibrillin 2, LIG-1, ProSAAS, STMN2, Nova1, Secretogranin II, Mab21l2, LIPA, LRRN3, Fibrillin, Plastin, SFRP4, REDD1, Pleiotrophin (OSF1), SERP1, Menin, MKP-3, Semaphorin 3A, Annexin I, Ephrin-B, IBP, CD166, 14-3-3 epsilon, Osteonectin, LAMG1, CTGF, ARGBP2, Osteopontin, FEZ2, COL4A1, FN14(TNFRSF12A), Collagen IV, 14-3-3, IGFBP7/8

AK3, FGFR3, CTGF, LAMB1, IDH1, Galpha(i)-specific EDG GPCRs, MyHC, TLE, Tropomyosin, CAPZ beta, EGR2 (Krox20), SFRP1, Carbohydrate sulfotransferases, CYP26A1, BMP receptor 2, K-cadherin (CDH6), Ephrin-A receptor 4, STMN2, Alpha-actinin 1, NAP1, ASSY, Secretogranin II, HES5, Fibrillin, CDK1 (p34), ACTG2, MMD, ERM, BETA3, POU class III, RILP (REST-interacting LIM domain protein), POU3F2 (BRN2), Calcineurin B (regulatory), SOX3, SNAP-25, FGF14, DLL3, SLC7A5, Ephrin-B, EZH2, WNT, CD166, LAMG1, Beta-adaptin 2, C/EBPdelta, APP, Actin, Osteopontin, FEZ2, Alpha-internexin, Collagen IV, ENO3, Tropomyosin-1, IGFBP7/8, VEGF-B, Galpha(q)-specific EDG GPCRs

3. Multicellular organism development

6641	8.401E-31	8.401E-31	193	Ephrin-B3, Alpha-N-catenin, Alpha-actinin, c-Maf, COL1A1, SOCS2, HMG1,2, AK3L1, PRICKLE2, DR6(TNFRSF21), HSP47, Paralemmin, IRX2, PDE5A, Aurora-B, COL5A2, LIPA, Ephrin-A receptors, NEBL, JNK(MAPK8-10), LRRN3, MYH9, MEGF10, Plastin, BDNF, HAS, LEC2, LRFN5, Pleiotrophin (OSF1), MKP-3, Lamin B, FOXC1/2, T-plastin, VCAM1, Collagen V, APOE, ARS2, BMP5, FANCG, WDR18, NMP200, NEFM, COL4A1, SPRY1, WNT3A, SLIT3, Podocalyxin-like 1, CEL, FZD1, C/EBP, Tau (MAPT), Fibrillin 2, COL11A1, RhoC, LIG-1, Adrenomedullin, ID2, TLE1, TIM, BChE, Mab21l2, Transgelin, Irx6, Galpha(q)-specific peptide GPCRs, ID3, FOXC1, G6PD, UCHL1, Synaptotagmin IV, DPPA4, DEC1 (Stra13), MAP6, REDD1, WNT3, Synaptotagmin, NEFL, Doublecortin, Caspr2, HMG2, Calcineurin B1, PDE, PTPR-zeta, IBP, ASNS, HOOK1, ENO, COL1A2, Alpha-catenin, FN14(TNFRSF12A), FAM65B, ID1, MAP-1B, Neurogenin 2, CHD8, FLRT3, PTK7, Carboxypeptidase H, Atlastin, Tissue kallikreins, SNAPs, A2M, Stathmin, ZIC2, NPY5R, Nova1, EGR1, ACTA2, SLIT2, PAFAH gamma, Contactin 2, Frizzled, WDR5, ENC1, CX3CL1, Shootin1 (KIAA1598), Galpha(i)-specific peptide GPCRs, Collagen I, TAFs, MAP4, MYT1, DNA-PK, Menin, Semaphorin 3A, Antigen KI-67, Connexin 43, EBF4, HDL proteins,	8.250E-10	46	EBF3, Carboxypeptidase H, CEL, CYP26A1, Ephrin-B3, SNAPs, EMP1 (Tmp), FKBP12, Fibrillin 2, LIG-1, Osteocrin, ProSAAS, STMN2, Nova1, Secretogranin II, Mab21l2, LIPA, LRRN3, Fibrillin, Plastin, SFRP4, CRABP1, REDD1, Pleiotrophin (OSF1), SERP1, Menin, MKP-3, Semaphorin 3A, Annexin I, EBF4, Ephrin-B, IBP, CD166, 14-3-3 epsilon, Osteonectin, LAMG1, CTGF, ARGBP2, Osteopontin, FEZ2, COL4A1, FN14(TNFRSF12A), Collagen IV, 14-3-3, IGFBP7/8, Fibulin-1

SIX5, Actin muscle, DNER, Osteonectin, AK3, FGFR3, CTGF, LAMB1, IDH1, Galpha(i)-specific EDG GPCRs, MyHC, TLE, Tropomyosin, CAPZ beta, EGR2 (Krox20), SFRP1, EBF3, Carbohydrate sulfotransferases, CYP26A1, BMP receptor 2, K-cadherin (CDH6), EMP1 (Tmp), LAF4, Ephrin-A receptor 4, STMN2, Alpha-actinin 1, NAP1, ASSY, Secretogranin II, HES5, Fibrillin, CDK1 (p34), ACTG2, Mcl-1, MMD, ERM, BETA3, POU class III, RILP (REST-interacting LIM domain protein), POU3F2 (BRN2), CRABP1, Calcineurin B (regulatory), SOX3, SNAP-25, FGF14, DLL3, SLC7A5, PCDH8, Ephrin-B, EZH2, WNT, CD166, LAMG1, Beta-adaptin 2, C/EBPdelta, APP, Actin, Osteopontin, FEZ2, Alpha-internexin, Collagen IV, ENO3, Tropomyosin-1, IGFBP7/8, VEGF-B, Galpha(q)-specific EDG GPCRs

4. Neurogenesis

	2479	1.727E-30	1.727 E-30	112	Ephrin-B3, Alpha-N-catenin, Alpha-actinin, SOCS2, HMG1,2, PRICKLE2, DR6(TNFRSF21), Paralemmin, COL5A2, Ephrin-A receptors, JNK(MAPK8-10), LRRN3, MYH9, BDNF, LRFN5, Pleiotrophin (OSF1), MKP-3, Lamin B, T-plastin, Collagen V, APOE, ARS2, BMP5, NMP200, NEFM, COL4A1, WNT3A, SLIT3, FZD1, C/EBP, Tau (MAPT), RhoC, Adrenomedullin, ID2, BChE, Irx6, Galpha(q)-specific peptide GPCRs, ID3, UCHL1, Synaptotagmin IV, MAP6, REDD1, WNT3, Synaptotagmin, NEFL, Doublecortin, Caspr2, HMG2, Calcineurin B1, PDE, PTPR-zeta, Alpha-catenin, FN14(TNFRSF12A), ID1, MAP-1B, Neurogenin 2, FLRT3, PTK7, Atlastin, Tissue kallikreins, SNAPs, Stathmin, ZIC2, EGR1, SLIT2, Contactin 2, Frizzled, WDR5, ENC1, CX3CL1, Shootin1 (KIAA1598), Galpha(i)-specific peptide GPCRs, MAP4, Semaphorin 3A, Connexin 43, HDL proteins, DNER, FGFR3, LAMB1, Galpha(i)-specific EDG GPCRs, MyHC, CAPZ beta, EGR2 (Krox20), SFRP1, Carbohydrate sulfotransferases, BMP receptor 2, Ephrin-A receptor 4, STMN2, NAP1, HES5, CDK1 (p34), MMD, ERM, BETA3, POU class III, POU3F2 (BRN2), Calcineurin B (regulatory), SOX3, SNAP-25, DLL3, Ephrin-B, EZH2, WNT, CD166, LAMG1, Beta-adaptin 2, APP, Actin, Osteopontin, FEZ2, Collagen IV,	1.811E-04	19	Ephrin-B3, SNAPs, STMN2, LRRN3, REDD1, Pleiotrophin (OSF1), MKP-3, Semaphorin 3A, Annexin I, Ephrin-B, CD166, 14-3-3 epsilon, LAMG1, Osteopontin, FEZ2, COL4A1, FN14(TNFRSF12A), Collagen IV, 14-3-3

					Galpha(q)-specific EDG GPCRs			
5. Anatomical structure development								
	7180	7.365E-30	7.365E-30	200	Ephrin-B3, Alpha-N-catenin, Alpha-actinin, c-Maf, COL1A1, SOCS2, HMG1,2, AK3L1, PRICKLE2, DR6(TNFRSF21), HSP47, Paralemmin, IRX2, PDE5A, Aurora-B, COL5A2, LIPA, Ephrin-A receptors, NEBL, JNK(MAPK8-10), LRRN3, MYH9, MEGF10, Plastin, BDNF, HAS, LEC2, LRFN5, Pleiotrophin (OSF1), MKP-3, Lamin B, FOXC1/2, T-plastin, VCAM1, TMEFF2, Collagen V, APOE, ARS2, BMP5, FANCG, WDR18, NMP200, NEFM, COL4A1, SPRY1, WNT3A, SLIT3, Podocalyxin-like 1, CEL, IEX1, FZD1, C/EBP, Tau (MAPT), Fibrillin 2, COL11A1, RhoC, LIG-1, Adrenomedullin, ID2, TLE1, TIM, BChE, Mab21l2, Transgelin, Irx6, Galpha(q)-specific peptide GPCRs, ID3, FOXC1, G6PD, UCHL1, Synaptotagmin IV, DPPA4, DEC1 (Stra13), MAP6, REDD1, WNT3, Synaptotagmin, NEFL, Doublecortin, Caspr2, HMG2, Calcineurin B1, PDE, PTPR-zeta, IBP, ASNS, HOOK1, ENO, COL1A2, Alpha-catenin, FN14(TNFRSF12A), FAM65B, ID1, MAP-1B, Neurogenin 2, CHD8, FLRT3, PTK7, Carboxypeptidase H, Atlastin, Tissue kallikreins, SNAPs, A2M, Stathmin, ZIC2, MEST, NPY5R, Nova1, EGR1, ACTA2, SLIT2, PAFAH gamma, Contactin 2, Frizzled, WDR5, ENC1, CX3CL1, Shootin1 (KIAA1598), Galpha(i)-specific peptide	3.204E-09	47	EBF3, Carboxypeptidase H, CEL, CYP26A1, Ephrin-B3, SNAPs, EMP1 (Tmp), FKBP12, Fibrillin 2, LIG-1, Osteocrin, ProSAAS, STMN2, Nova1, Secretogranin II, Mab21l2, LIPA, LRRN3, Fibrillin, Plastin, SFRP4, CRABP1, REDD1, Pleiotrophin (OSF1), SERP1, Menin, MKP-3, Semaphorin 3A, Annexin I, EBF4, CENPJ, Ephrin-B, IBP, CD166, 14-3-3 epsilon, Osteonectin, LAMG1, CTGF, ARGBP2, Osteopontin, FEZ2, COL4A1, FN14(TNFRSF12A), Collagen IV, 14-3-3, IGFBP7/8, Fibulin-1

GPCRs, Collagen I, TAFs, MAP4, MYT1, DNA-PK, Menin, Semaphorin 3A, Antigen KI-67, Connexin 43, EBF4, HDL proteins, SIX5, Actin muscle, DNER, Osteonectin, AK3, FGFR3, CTGF, LAMB1, IDH1, S1P3 receptor, UGCG, Galpha(i)-specific EDG GPCRs, MyHC, TLE, Tropomyosin, CAPZ beta, EGR2 (Krox20), SFRP1, EBF3, Carbohydrate sulfotransferases, CYP26A1, BMP receptor 2, K-cadherin (CDH6), EMP1 (Tmp), LAF4, Ephrin-A receptor 4, STMN2, Alpha-actinin 1, NAP1, ASSY, Secretogranin II, HES5, Fibrillin, CDK1 (p34), ACTG2, Mcl-1, Ca-ATPase2, MMD, ERM, BETA3, POU class III, RILP (REST-interacting LIM domain protein), POU3F2 (BRN2), CRABP1, Calcineurin B (regulatory), SOX3, UBC6, SNAP-25, FGF14, DLL3, SLC7A5, PCDH8, Ephrin-B, EZH2, WNT, CD166, LAMG1, Beta-adaptin 2, C/EBPdelta, APP, Actin, Osteopontin, FEZ2, Alpha-internexin, Collagen IV, ENO3, Tropomyosin-1, IGFBP7/8, VEGF-B, Galpha(q)-specific EDG GPCRs

6. Cell development

	2496	1.423E-29	1.423 E-29	111	Ephrin-B3, Alpha-N-catenin, Alpha-actinin, c-Maf, HMG1,2, PRICKLE2, HSP47, COL5A2, Ephrin-A receptors, NEBL, JNK(MAPK8-10), LRRN3, MYH9, MEGF10, BDNF, HAS, LRFN5, MKP-3, FOXC1/2, Collagen V, APOE, BMP5, FANCG, NEFM, COL4A1, WNT3A, SLIT3, Podocalyxin-like 1, C/EBP, Tau (MAPT), COL11A1, RhoC, Adrenomedullin, ID2, Galpha(q)-specific peptide GPCRs, FOXC1, G6PD, UCHL1, MAP6, WNT3, NEFL, Doublecortin, Caspr2, HMG2, Calcineurin B1, PDE, PTPR-zeta, HOOK1, Alpha-catenin, FAM65B, ID1, MAP-1B, Neurogenin 2, FLRT3, Atlastin, Tissue kallikreins, Stathmin, ZIC2, ACTA2, SLIT2, Contactin 2, Frizzled, WDR5, Shootin1 (KIAA1598), Galpha(i)-specific peptide GPCRs, TAFs, MAP4, DNA-PK, Menin, Semaphorin 3A, Connexin 43, HDL proteins, SIX5, Actin muscle, DNER, FGFR3, LAMB1, Galpha(i)-specific EDG GPCRs, MyHC, Tropomyosin, CAPZ beta, EGR2 (Krox20), SFRP1, Carbohydrate sulfotransferases, CYP26A1, BMP receptor 2, Ephrin-A receptor 4, STMN2, Alpha-actinin 1, HES5, CDK1 (p34), Ca-ATPase2, ERM, BETA3, POU class III, POU3F2 (BRN2), Calcineurin B (regulatory), SOX3, UBC6, SNAP-25, Ephrin-B, WNT, CD166, LAMG1, Beta-adaptin 2, APP, Actin, FEZ2, Collagen IV, Tropomyosin-1, Galpha(q)-specific EDG GPCRs	1.023E-02	15	CYP26A1, Ephrin-B3, STMN2, LRRN3, Menin, MKP-3, Semaphorin 3A, Ephrin-B, CD166, LAMG1, ARGBP2, FEZ2, COL4A1, Collagen IV, 14-3-3

7. Single-multicellular organism process

7650	4.031E-29	4.031 E-29	206	Ephrin-B3, Alpha-N-catenin, Alpha-actinin, c-Maf, COL1A1, SOCS2, HMG1,2, AK3L1, PRICKLE2, DR6(TNFRSF21), HSP47, Paralemmin, IRX2, PDE5A, ABCA1, Aurora-B, COL5A2, IRF9, LIPA, Ephrin-A receptors, NEBL, JNK(MAPK8-10), LRRN3, Optineurin, MYH9, MEGF10, Plastin, BDNF, HAS, LEC2, LRFN5, Pleiotrophin (OSF1), MKP-3, Lamin B, FOXC1/2, T-plastin, Kid, VCAM1, Collagen V, APOE, ARS2, IRF7, BMP5, FANCG, WDR18, NMP200, NEFM, COL4A1, SPRY1, WNT3A, SLIT3, Podocalyxin-like 1, CEL, FZD1, C/EBP, Tau (MAPT), Fibrillin 2, COL11A1, RhoC, LIG-1, Adrenomedullin, ID2, TLE1, TIM, BChE, Multimerin, Mab21l2, Transgelin, TFPI-2, Irx6, Galpha(q)-specific peptide GPCRs, ID3, FOXC1, G6PD, UCHL1, Synaptotagmin IV, DPPA4, Tubulin alpha, DEC1 (Stra13), MAP6, REDD1, WNT3, Synaptotagmin, NEFL, Doublecortin, Caspr2, HMG2, Calcineurin B1, PDE, PTPR-zeta, IBP, ASNS, HOOK1, ENO, COL1A2, Alpha-catenin, FN14(TNFRSF12A), FAM65B, ID1, MAP-1B, Neurogenin 2, CHD8, FLRT3, PTK7, Carboxypeptidase H, Atlastin, Tissue kallikreins, SNAPs, A2M, Stathmin, ZIC2, NPY5R, Nova1, EGR1, ACTA2, SLIT2, PAFAH gamma, Contactin 2, Frizzled, WDR5, ENC1, CX3CL1, Shootin1 (KIAA1598), Galpha(i)-specific peptide GPCRs, Collagen I, TAFs, MAP4, MYT1,	1.65 1E-11	52	EBF3, Carboxypeptidase H, CEL, CYP26A1, Ephrin-B3, SNAPs, EMP1 (Tmp), FKBP12, Fibrillin 2, LIG-1, Osteocrin, ProSAAS, Multimerin, STMN2, Nova1, Secretogranin II, Mab21l2, LIPA, LRRN3, TFPI-2, Fibrillin, IBP3, Plastin, Raftlin, SFRP4, CRABP1, LPL, REDD1, Pleiotrophin (OSF1), SERP1, Menin, MKP-3, Semaphorin 3A, Annexin I, EBF4, Ephrin-B, IBP, CD166, 14-3-3 epsilon, Osteonectin, LAMG1, CTGF, ARGBP2, Osteopontin, FEZ2, COL4A1, FN14(TNFRSF12A), Collagen IV, 14-3-3, IGFBP7/8, Fibulin-1, AMYS

DNA-PK, LPL, Menin, Semaphorin 3A, Antigen KI-67, Connexin 43, EBF4, HDL proteins, SIX5, Actin muscle, DNER, Osteonectin, AK3, FGFR3, CTGF, LAMB1, IDH1, S1P3 receptor, Galpha(i)-specific EDG GPCRs, MyHC, TLE, Tropomyosin, CAPZ beta, EGR2 (Krox20), SFRP1, EBF3, Carbohydrate sulfotransferases, CYP26A1, BMP receptor 2, K-cadherin (CDH6), EMP1 (Tmp), LAF4, Ephrin-A receptor 4, STMN2, Alpha-actinin 1, NAP1, ASSY, Secretogranin II, HES5, Fibrillin, CDK1 (p34), ACTG2, Mcl-1, Ca-ATPase2, MMD, ERM, BETA3, POU class III, RILP (REST-interacting LIM domain protein), IBP3, Raftlin, POU3F2 (BRN2), CRABP1, Calcineurin B (regulatory), SOX3, SNAP-25, FGF14, DLL3, SLC7A5, PCDH8, Ephrin-B, EZH2, WNT, CD166, LAMG1, Beta-adaptin 2, C/EBPdelta, APP, Actin, Osteopontin, FEZ2, Alpha-internexin, Collagen IV, ENO3, Tropomyosin-1, IGFBP7/8, VEGF-B, Galpha(q)-specific EDG GPCRs

8. Generation of neurons

	2361	4.682E-29	4.682 E-29	107	Ephrin-B3, Alpha-N-catenin, Alpha-actinin, SOCS2, HMG1,2, PRICKLE2, DR6(TNFRSF21), Paralemmin, COL5A2, Ephrin-A receptors, JNK(MAPK8-10), LRRN3, MYH9, BDNF, LRFN5, Pleiotrophin (OSF1), MKP-3, T-plastin, Collagen V, APOE, ARS2, BMP5, NMP200, NEFM, COL4A1, WNT3A, SLIT3, FZD1, C/EBP, Tau (MAPT), RhoC, Adrenomedullin, ID2, BChE, Irx6, Galpha(q)-specific peptide GPCRs, ID3, UCHL1, Synaptotagmin IV, MAP6, REDD1, WNT3, Synaptotagmin, NEFL, Doublecortin, Caspr2, HMG2, PDE, PTPR-zeta, Alpha-catenin, FN14(TNFRSF12A), ID1, MAP-1B, Neurogenin 2, FLRT3, PTK7, Atlastin, Tissue kallikreins, SNAPs, Stathmin, ZIC2, SLIT2, Contactin 2, Frizzled, WDR5, ENC1, CX3CL1, Shootin1 (KIAA1598), Galpha(i)-specific peptide GPCRs, MAP4, Semaphorin 3A, Connexin 43, HDL proteins, DNER, FGFR3, LAMB1, Galpha(i)-specific EDG GPCRs, MyHC, CAPZ beta, EGR2 (Krox20), SFRP1, Carbohydrate sulfotransferases, BMP receptor 2, Ephrin-A receptor 4, STMN2, HES5, CDK1 (p34), MMD, ERM, BETA3, POU class III, POU3F2 (BRN2), SOX3, SNAP-25, DLL3, Ephrin-B, EZH2, WNT, CD166, LAMG1, Beta-adaptin 2, APP, Actin, Osteopontin, FEZ2, Collagen IV, Galpha(q)-specific EDG GPCRs	3.00 0E-04	18	Ephrin-B3, SNAPs, STMN2, LRRN3, REDD1, Pleiotrophin (OSF1), MKP-3, Semaphorin 3A, Ephrin-B, CD166, 14-3-3 epsilon, LAMG1, Osteopontin, FEZ2, COL4A1, FN14(TNFRSF12A), Collagen IV, 14-3-3

9. Developmental process

7560	3.151E-28	3.151 E-28	203	Ephrin-B3, Alpha-N-catenin, Alpha-actinin, c-Maf, COL1A1, SOCS2, HMG1,2, AK3L1, PRICKLE2, DR6(TNFRSF21), HSP47, Paralemmin, IRX2, PDE5A, Aurora-B, COL5A2, LIPA, Ephrin-A receptors, NEBL, JNK(MAPK8-10), LRRN3, MYH9, MEGF10, Plastin, BDNF, HAS, LEC2, LRFN5, Pleiotrophin (OSF1), MKP-3, Lamin B, FOXC1/2, T-plastin, VCAM1, TMEFF2, Collagen V, APOE, ARS2, ELF4, BMP5, FANCG, WDR18, NMP200, NEFM, COL4A1, SPRY1, WNT3A, SLIT3, Podocalyxin-like 1, CEL, IEX1, FZD1, C/EBP, Tau (MAPT), Fibrillin 2, COL11A1, RhoC, LIG-1, Adrenomedullin, ID2, TLE1, TIM, BChE, Mab21l2, Transgelin, Irx6, Galpha(q)-specific peptide GPCRs, ID3, FOXC1, G6PD, UCHL1, Synaptotagmin IV, DPPA4, DEC1 (Stra13), MAP6, REDD1, WNT3, Synaptotagmin, NEFL, Doublecortin, Caspr2, HMG2, Calcineurin B1, PDE, PTPR-zeta, IBP, ASNS, HOOK1, ENO, COL1A2, Alpha-catenin, FN14(TNFRSF12A), FAM65B, ID1, MAP-1B, Neurogenin 2, CHD8, FLRT3, PTK7, Carboxypeptidase H, Atlastin, Tissue kallikreins, SNAPs, A2M, Stathmin, ZIC2, MEST, NPY5R, Nova1, EGR1, ACTA2, SLIT2, PAFAH gamma, Contactin 2, Frizzled, WDR5, ENC1, CX3CL1, Shootin1 (KIAA1598), Galpha(i)-specific peptide GPCRs, Collagen I, TAFs, MAP4, MYT1, DNA-PK, Menin, Semaphorin 3A, Antigen	5.04 4E-09	48	EBF3, Carboxypeptidase H, CEL, CYP26A1, Ephrin-B3, SNAPs, EMP1 (Tmp), FKBP12, Fibrillin 2, LIG-1, Osteocrin, ProSAAS, STMN2, Nova1, Secretogranin II, Mab21l2, LIPA, LRRN3, Fibrillin, IBP3, Plastin, SFRP4, CRABP1, REDD1, Pleiotrophin (OSF1), SERP1, Menin, MKP-3, Semaphorin 3A, Annexin I, EBF4, CENPJ, Ephrin-B, IBP, CD166, 14-3-3 epsilon, Osteonectin, LAMG1, CTGF, ARGBP2, Osteopontin, FEZ2, COL4A1, FN14(TNFRSF12A), Collagen IV, 14-3-3, IGFBP7/8, Fibulin-1

KI-67, Connexin 43, EBF4, HDL proteins, SIX5, Actin muscle, DNER, Osteonectin, AK3, FGFR3, CTGF, LAMB1, IDH1, S1P3 receptor, UGCG, Galpha(i)-specific EDG GPCRs, MyHC, TLE, Tropomyosin, CAPZ beta, EGR2 (Krox20), SFRP1, EBF3, Carbohydrate sulfotransferases, CYP26A1, BMP receptor 2, K-cadherin (CDH6), EMP1 (Tmp), LAF4, Ephrin-A receptor 4, STMN2, Alpha-actinin 1, NAP1, ASSY, Secretogranin II, HES5, NQO1, Fibrillin, CDK1 (p34), ACTG2, Mcl-1, Ca-ATPase2, MMD, ERM, BETA3, POU class III, RILP (REST-interacting LIM domain protein), IBP3, POU3F2 (BRN2), CRABP1, Calcineurin B (regulatory), SOX3, UBC6, SNAP-25, FGF14, DLL3, SLC7A5, PCDH8, Ephrin-B, EZH2, WNT, CD166, LAMG1, Beta-adaptin 2, C/EBPdelta, APP, Actin, Osteopontin, FEZ2, Alpha-internexin, Collagen IV, ENO3, Tropomyosin-1, IGFBP7/8, VEGF-B, Galpha(q)-specific EDG GPCRs

10. Regulation of developmental process								
	3134	4.216E-28	4.216E-28	123	Alpha-N-catenin, Alpha-actinin, c-Maf, COL1A1, SOCS2, HMG1,2, PRICKLE2, DR6(TNFRSF21), Paralemmin, Septin 9, PDE5A, ABCA1, TMEM90B, COL5A2, Sts-1, Ephrin-A receptors, JNK(MAPK8-10), LRRN3, MYH9, MEGF10, BDNF, HAS, LEC2, Pleiotrophin (OSF1), MKP-3, FOXC1/2, Collagen V, APOE, ARS2, IRF7, BMP5, NMP200, NEFM, SPRY1, WNT3A, SLIT3, FZD1, NEK6, DIO3, C/EBP, Tau (MAPT), Fibrillin 2, Adrenomedullin, ID2, Galpha(q)-specific peptide GPCRs, ID3, FOXC1, G6PD, Synaptotagmin IV, WNT3, Synaptotagmin, NEFL, DAAM1, HMG2, PDE, DDAH1, PTPR-zeta, IBP, Vasorin, Alpha-catenin, FN14(TNFRSF12A), ID1, MAP-1B, Neurogenin 2, FLRT3, PTK7, Carboxypeptidase H, Tissue kallikreins, ZIC2, EGR1, SLIT2, Contactin 2, Frizzled, PALMD, ENC1, CX3CL1, Shootin1 (KIAA1598), Galpha(i)-specific peptide GPCRs, Collagen I, TAFs, MAP4, DNA-PK, LPL, Menin, Semaphorin 3A, Connexin 43, HDL proteins, Osteonectin, FGFR3, CTGF, S1P3 receptor, Galpha(i)-specific EDG GPCRs, MyHC, Tropomyosin, CAPZ beta, TD-60, SFRP1, BMP receptor 2, Ephrin-A receptor 4, STMN2, HES5, Fibrillin, CDK1 (p34), MMD, ERM, POU class III, RILP (REST-interacting LIM domain protein), IBP3, POU3F2 (BRN2), SOX3, SNAP-25, DLL3, Ephrin-B, EZH2, WNT, C/EBPdelta, APP, Osteopontin, Collagen IV,	2.308E-08	29	Carboxypeptidase H, Fibrillin 2, Osteocrin, Septin 9, STMN2, LRRN3, Fibrillin, CLEC2D, IBP3, SFRP4, LPL, Pleiotrophin (OSF1), SERP1, Menin, MKP-3, Semaphorin 3A, Annexin I, CENPJ, Ephrin-B, IBP, Osteonectin, Vasorin, CTGF, Osteopontin, FN14(TNFRSF12A), Collagen IV, 14-3-3, IGFBP7/8, Fibulin-1

Tropomyosin-1, IGFBP7/8, VEGF-B,
Galpha(q)-specific EDG GPCRs

GO processes enriched by rApoE4 were compared to those compared to rApoE3 were analyzed using the MetaCoreTM software on GeneGo from Thomson Reuters for the three culture treatments. Input data for this analysis includes only genes for which the absolute FC values of +rApoE4 treatment/no rApoE treatment was 1.2 or more with a p value of $p \leq 0.05$. For the top process enriched for each culture treatment, the FC values of rApoE vs no rApoE with absolute values higher than 1.2 are listed
